# Mitochondrial Alterations by PARKIN in Dopaminergic Neurons Using PARK2 Patient-Specific and *PARK2* Knockout Isogenic iPSC Lines

**DOI:** 10.1016/j.stemcr.2015.02.019

**Published:** 2015-04-02

**Authors:** Atossa Shaltouki, Renuka Sivapatham, Ying Pei, Akos A. Gerencser, Olga Momčilović, Mahendra S. Rao, Xianmin Zeng

**Affiliations:** 1 Buck Institute, Novato, CA 94945, USA; 2 NxCell Science, Novato, CA 94947, USA; 3 XCell Science, Novato, CA 94947, USA

## Abstract

In this study, we used patient-specific and isogenic PARK2-induced pluripotent stem cells (iPSCs) to show that mutations in PARK2 alter neuronal proliferation. The percentage of TH^+^ neurons was decreased in Parkinson’s disease (PD) patient-derived neurons carrying various mutations in *PARK2* compared with an age-matched control subject. This reduction was accompanied by alterations in mitochondrial:cell volume fraction (mitochondrial volume fraction). The same phenotype was confirmed in isogenic *PARK2* null lines. The mitochondrial phenotype was also seen in non-midbrain neurons differentiated from the *PARK2* null line, as was the functional phenotype of reduced proliferation in culture. Whole genome expression profiling at various stages of differentiation confirmed the mitochondrial phenotype and identified pathways altered by PARK2 dysfunction that include PD-related genes. Our results are consistent with current model of PARK2 function where damaged mitochondria are targeted for degradation via a PARK2/PINK1-mediated mechanism.

## Highlights

• Human iPSC-based in vitro model for familial PD
• Mitochondrial deficit and accumulation of SNCA in dopaminergic neurons
• Isogenic controls recapitulate phenotype
• Microarray analysis identifies cell death and mitochondrial processing defects

## Teaser

PARKIN (*PARK2*) mutations are the most common cause of familial PD. In this article, Zeng and colleagues identify a mitochondrial deficit that confirms the role of PARK2 in mitochondrial function using four patient-derived iPSC lines carrying different *PARK2* mutations and isogenic controls. This study provides a human model system to assess PD therapy.

## Introduction

*PARKIN* (*PARK2*), an E3 ubiquitin ligase, is the most frequently mutated gene that has casually been linked to autosomal recessive early onset familial Parkinson’s disease (PD) (Abbas et al., 1999; Kitada et al., 1998). Abnormalities of *PARK2* have also been described in sporadic PD (Dawson, 2006). The exact mechanism by which *PARK2* causes PD-like syndromes and why dopaminergic neurons are primarily affected by a ubiquitously expressed mutation remain unknown (Sulzer, 2007; Tanaka et al., 2004). Several studies, however, suggest that *PARK2* interacts with *PINK1*, another gene mutated in autosomal recessive familial form of PD (Geisler et al., 2010) to regulate mitochondrial biology, and alters mitochondrial dynamics (Chen and Chan, 2009; Clark et al., 2006; Lee et al., 2004).

A link between PARK2 and mitochondrial biology was first established in *Drosophila*, which displayed impairment in mitochondrial function and neuronal loss in an age-dependent manner when rendered deficient for PARK2 (Greene et al., 2003). Likewise, similar mitochondrial defects exhibited in *Park2* knockout (KO) mouse models, although only mice with conditional KO of *Park2* recapitulate parkinsonian phenotype and striatonigral degeneration (Dawson et al., 2010; Goldberg et al., 2003). Analysis of single and double mutants in mice and flies also suggests that *Pink1* is upstream of *Park2* and that overexpression of PARK2 alone or directing PARK2 to mitochondria is sufficient to introduce mitochondrial fragmentation (Akundi et al., 2013; Clark et al., 2006; Kim et al., 2008; Shiba-Fukushima et al., 2012). Thus, both gain or loss of function can affect mitochondrial dynamics. More recently, post-mortem brain tissues of PD patients also confirmed the involvement of altered mitochondrial pathologies in disease process (Henchcliffe and Beal, 2008; Schapira et al., 1989; Vila et al., 2008).

The emerging hypothesis is that in normal cells PARK2 is cytoplasmic and PINK1 levels are low. However, when mitochondrial potential is lost, PINK1 accumulates on depolarized membranes and recruits PARK2 to mitochondria and are then targeted for degradation via mitophagy. Loss or damaged mitochondria stimulate mitochondrial fission and/or inhibit fusion by negatively regulating MFN and OPA1 function and/or positively regulating DRP1 (van der Bliek et al., 2013).

Despite these advances, differences between species in displaying neurodegenerative phenotypes have made it difficult to extrapolate the results obtained from animal models to human. The discovery of induced pluripotent stem cells (iPSCs) has for the first time enabled us to reproduce dopaminergic neurons from individuals who suffer from familial or sporadic PD. Indeed, a recent iPSC-based study showed that PARK2 controlled dopamine utilization in iPSC-derived dopaminergic neurons (Jiang et al., 2012). Likewise, advances in gene targeting (Cathomen and Joung, 2008; Urnov et al., 2010; Zeng et al., 2014) allow us to develop the corresponding models in an isogenic background.

To enable us to study the role of PARK2 in human PD, we made integration-free iPSC lines from four PD patients carrying different *PARK2* mutations (NINDS collection; Table S1). We showed a deficiency in dopaminergic differentiation and a reduction in mitochondrial volume fraction in all four PARK2 lines compared with an age-matched control subject. To confirm the results from the patient-specific disease model and to overcome the genetic variation among patient lines that could mask the PARK2 phenotype, we generated *PARK2* isogenic controls using a KO strategy in a well-characterized integration-free iPSC line. We found similar phenotypes in the *PARK2* KO isogenic line as seen from the familial PARK2 lines. We showed that loss-of-function mutations in PARK2 impaired dopaminergic development by reducing the percentage of Tyrosine hydroxylase-positive (TH^+^) neurons and accumulation of α-synuclein (SNCA) in dopaminergic neurons. These results were supported by whole genome expression profiling in which alterations in expression of mitochondria and cell death-related genes were observed in the dopaminergic neuron stage but not in earlier stages of differentiation. In addition, we showed that similar changes were detected in a pure population of forebrain neurons derived from the isogenic model. Our results suggest that PARK2 is involved in mitochondrial regulation in neurons.

## Results

### Generation of Integration-free iPSC Lines from Four Patients with Various *PARK2* Mutations

To investigate why mutations in PARK2 cause selective degeneration of dopaminergic neurons in humans, we first used a patient-specific-based-iPSC approach. Fibroblasts from four patients (I, P, B, S) with various mutations in *PARK2* and an aged-matched control subject (Y) were used to generate iPSC lines. Table S1 lists the clinical and demographic data associated with each cell line. Whole genome expression analysis was performed on the fibroblasts to obtain baseline data on the samples. No significant difference in overall gene expression in major PD genes was observed (Table S2).

**Figure 1:**
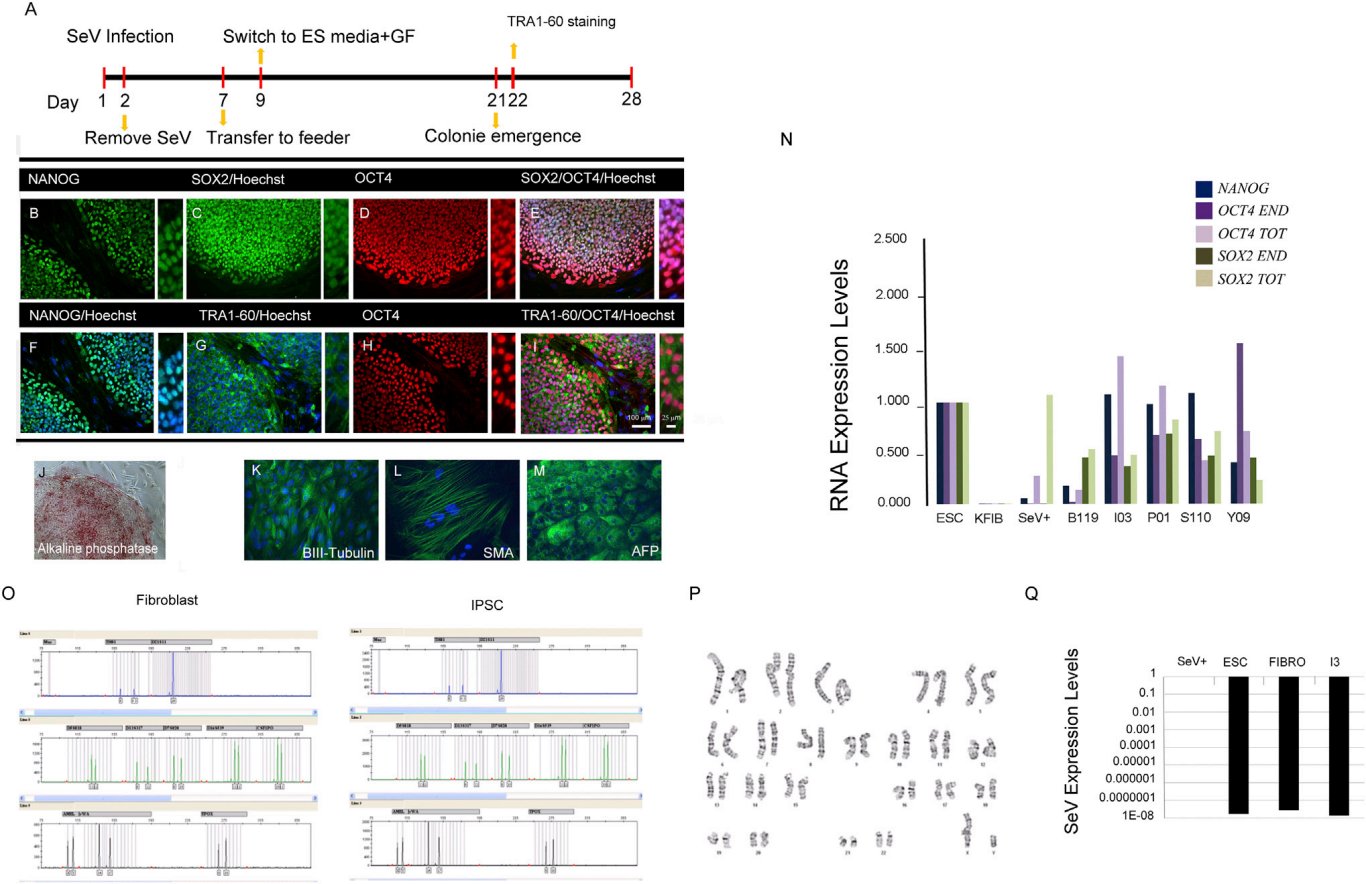
Generation and Characterization of Parkinson’s Disease-Derived iPSC Using Sendai Virus Vector (A) The workflow outlined here demonstrated the generation of human iPSC with Sendai virus vector encoding OCT4, KLF4, SOX2, and cMYC. Approximately 5 × 10^5^ human fibroblasts were plated onto a 35-mm dish 1 day before transduction. Day 1 denotes a day of transduction. About 6 to 7 days after transduction, cells were collected and transferred onto inactivated mouse feeders at the density of 5 × 10^5^ cells per 10-cm dish. (B–J) Approximately 3-weeks posttransduction, TRA1-60-positive colonies were picked manually and transferred to a fresh feeder-coated well. This shows representative colonies of PARK2 patient iPSCs stained for pluripotent markers NANOG, SOX2, OCT4, and TRA-1-60 (B–I), as well as colonies positive for alkaline phosphatase activity (J). (K–M) Immunofluorescence analysis of PARK2-PD-iPSC differentiated in vitro show the potential to generate cell derivatives of all three primary germ cell layers, including ectoderm (stained for TUJ-1, green), mesoderm (stained for smooth muscle actin [SMA], green), and endoderm (stained for a-fetoprotein, green). (N) qPCR analyses of the endogenous (genomic) and exogenous (Sendai virus vector) expression levels of the indicated genes in PARK2 patient iPSCs. (O) Short tandem repeat (STR) analysis of genomic DNA from PARK2-PD-iPSC matched the identity of iPSCs to their parent fibroblasts. (P) Normal karyotype of PARK2 patient iPSC at passage 20. (Q) The absence of persistent Sendai virus in I3 PARK2 patient fibroblasts and iPSC was confirmed as determined by RT-PCR analysis. Results are representative of three biological replicates (individual clones in case of iPSC) from three independent experiments. Scale bars are 100 μM.

Integration-free iPSC lines were generated by Sendai technology (Cathomen and Joung, 2008; Pavletich and Pabo, 1991; Wang et al., 2013; Yang et al., 2008). Multiple clones from each subject were isolated and expanded and validated for pluripotency ability to differentiate to three germ layers absence of vector integration in iPSC lines and identity by STR analysis and normal karyotype over long-term culture (>20 passages) in vitro (Figures 1A–1P). One clone of each line referred as Y09 (control), I3, P1, S110, and B119 (PARK2 patients) was chosen for this study.

At this stage, no difference was observed between the control line and the four PARK2 patient lines by growth rate, morphology, and whole genome analysis. Thus, neither the fibroblasts nor the iPSC derived from them displayed an obvious phenotype. PARK2 levels were low or undetectable in normal fibroblasts and normal iPSC lines, suggesting a possible explanation.

### Neural and Neuronal Differentiation of PARK2 and Control iPSC Lines

**Figure 2:**
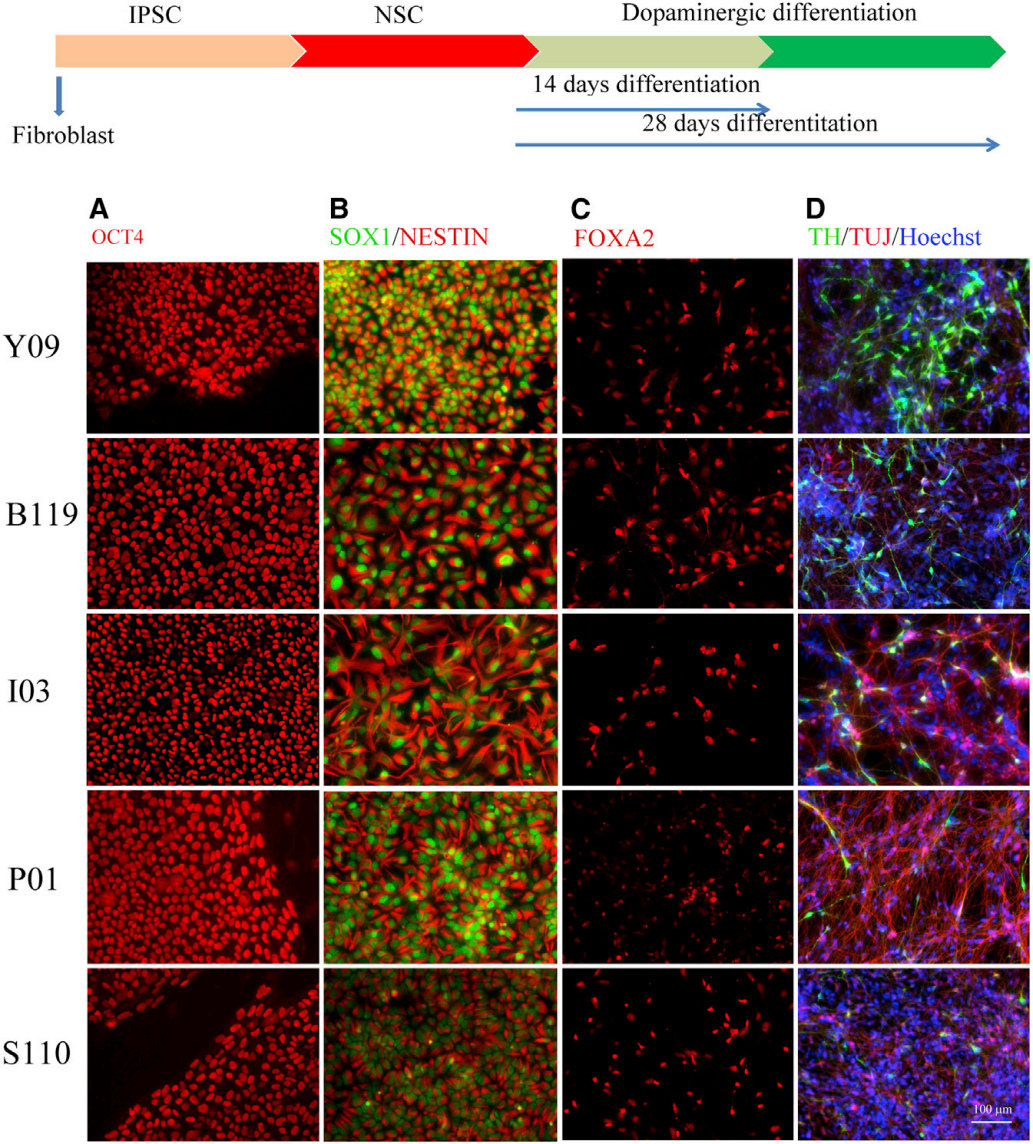
Dopaminergic Differentiation of Integration-free iPSC Lines Generated from Four PARK2 Patients and One Control (A) A scheme of differentiation of dopaminergic neurons from patient-derived iPSC. (B) Patient-derived iPSC and iPSC- from the age-matched control subject were directed to neuroepithelial cells using the previously described protocol (Swistowski et al., 2009). (C) These neuroepithelial cells were exposed to PA6-CM to produce FOXA2 expressing dopaminergic progenitors in the subsequent 2 weeks. (D) Finally, the progenitors were further differentiated to post-mitotic dopaminergic neurons by the addition of neurotrophic factors to PA6-CM, as described previously. Control and patient-specific iPSC-derived neurons were analyzed by immunofluorescence for expression of TH (green) and TUJ-1 (red) at the end of the 28-day differentiation protocol. Shown are representative images of three independent differentiation experiments from Control-iPSC, PARK2 patient iPSC lines carrying different *PARK2* mutations. Scale bars = 100 μM.

We next determined whether the PARK2 iPSC lines could differentiate to neuronal lineage. We observed no different NSC formation between the patient and control lines, and all lines differentiated into dopaminergic neurons (Figures 2A–2D). The experiment was repeated several times (n = 4), and no difference was observed in any biological replicates. Whole genome profiling of each line at various stages of differentiation could not distinguish the patient samples from the control (Figure S1). Instead, samples were clustered by cell types as expected: hierarchial clustering of NSC and dopaminergic neurons revealed a similar gene expression pattern for all of the iPSC generated. The overall correlation coefficiency between each population showed highest similarities (higher R^2^ value) among each population (e.g., iPSC versus iPSC, R^2^ > 0.98) (Table S3). Examining the dataset at a higher resolution also showed appropriate temporal expression of stage specific markers such as LMX1A, FOXA2, and AADC, which were expressed in all the generated lines at the dopaminergic stage (data not shown), similar to what we had previously observed in our experiments (Liu et al., 2013; Momčilović et al., 2014).

### Impaired Dopaminergic Differentiation of PARK2 Lines

**Figure 3:**
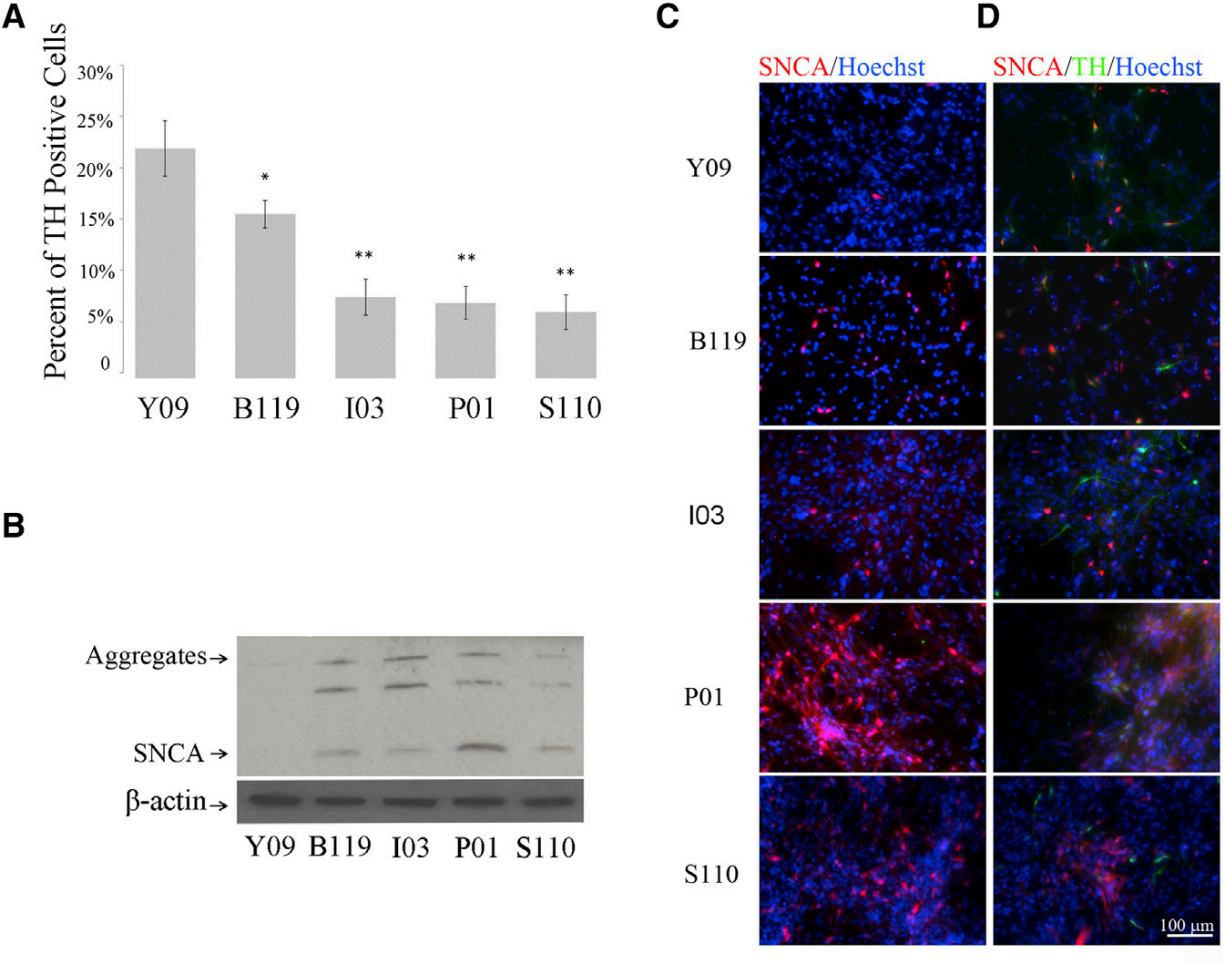
Decreased TH-Positive Neurons and Increased SNCA Expression in iPSC Lines Carrying *PARK2* Mutations (A) To determine differentiation efficiency, the number TH-positive cells is represented as the percentage of total number of cells (stained with Hoechst). Error bars represent mean SEM of triplicates from four independent experiments. Significant differences were found in the ability of iPSCs from different patients to generate dopaminergic neurons after 28 days of differentiation. (B) Western blot analysis of extracts from patient-derived neurons probed with SNCA antibodies. The arrow marks the 19-kDa SNCA present in patient lines and absent in control healthy subject. The band just above (present in only patient lines) may represent an alternatively spliced form of SNCA or aggregate form of SNCA. (C) Immunofluorescence for expression of SNCA in dopaminergic neurons from patient lines. Increased SNCA expression is observed in PARK2 patient lines. (D) Double stain of SNCA and TH expression in dopaminergic neurons from patient lines. SNCA is expressed in both TH-positive and non-TH-expressing neurons. Shown are representative of four independent experiments. Scale bars represent 100 μM.

Although the ability of the normal and PARK2 patient NSC differentiated in a qualitatively similar fashion, we did note that the efficacy of dopaminergic differentiation appeared to be reduced in the patient lines. To obtain a more quantitative measure, we counted TH^+^ cells in each line by immunocytochemistry and observed a significant decrease in TH^+^ neurons from the patient lines: about 22% of total cells expressed TH in the control line, whereas only 15%, 7%, 7% and 7% of total cells were TH^+^ dopaminergic neurons from B119, I3, P1, and S110 PARK2 lines, respectively (Figure 3A). We repeated the experiment several times (n = 4), and although the actual percentages varied in all cases, the number of TH^+^ cells in the PARK2 lines was always less than in the control. Given the temporally appropriate onset of dopaminergic precursor markers in the array analysis of patient and control lines, we interpret this result to suggest that the changes we observed were likely the result of death of differentiating or fully differentiated cells in the patient-derived cultures rather than fewer dopaminergic neurons being born in the patient lines.

Since death of dopaminergic neurons in PD is often accompanied by changes in SNCA protein, we examined SNCA expression in dopaminergic neurons of the patient and control lines. As seen in Figure 3B, SNCA or its aggregates were not detected in dopaminergic neurons derived from the control line. However, SNCA and two forms of SNCA aggregates were all elevated in all patient lines by western blot (Figure 3B). The increased expression of SNCA in PARK2 patient lines was also observed by immunostaining of SNCA: approximately 4.6% of total cells were SNCA^+^ in the control Y09 line, whereas approximately 12.1%, 7.4%, 8.5% and 9.0% of total cells were SNCA positive in the patient lines I3, P1, B119, and S110, respectively (Figures 3C and 3D). Double immunostaining of TH and SNCA revealed that enhanced SNCA expression was not limited to TH^+^ cells, as only a small percentage of the SNCA-positive cells were TH^+^ neurons for all lines (1.3% for the control Y09 line and 2.4%, 0.8%, 3.2%, and 0.6% for the patient lines I3, P1, B119, and S110, respectively) (Figure 3D). These results suggested that mutations in *PARK2* gene may contribute to stress in neuronal cultures that leads to the accumulation of SNCA in dopaminergic neurons. The accumulation of aggregates may contribute to the reduction in the number of TH^+^ neurons observed in the differentiating cultures form *PARK2* mutated patient lines.

### Reduced Mitochondrial Volume Fraction in PARK2 Patient Lines

**Figure 4:**
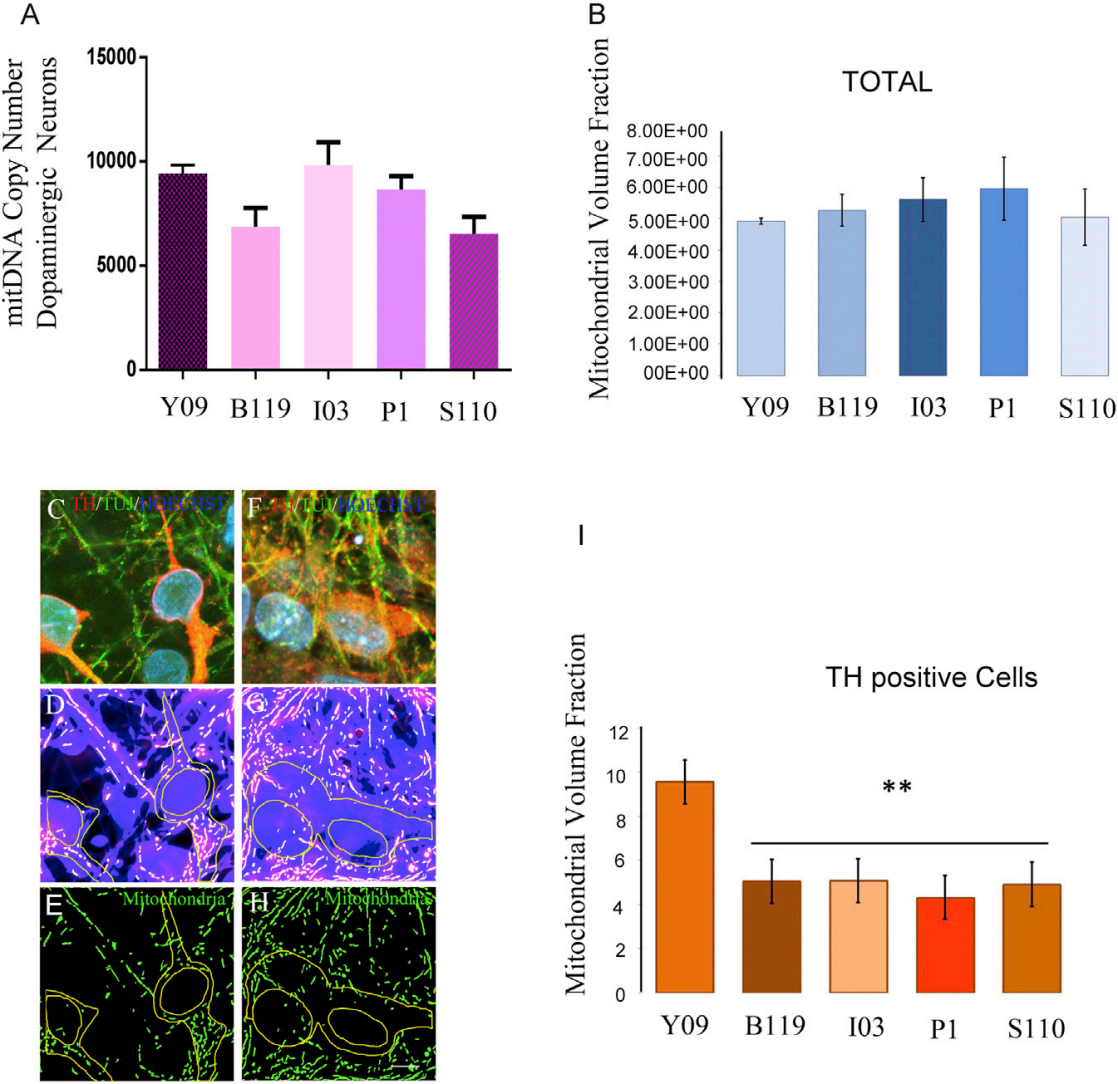
Changes in Mitochondrial Content in PARK2 Patient-Derived Dopaminergic Neurons (A) A variation of mtDNA copy number in patient-derived dopaminergic neuron mixed culture. Bars represent average with SEM as error bars. Results are representative of three independent experiments. (B–H) Mitochondrial volume fraction determination using confocal microscopy. The ratio of mitochondria (MitoTracker Red; green) to total cellular volume (Calcein-AM; gray) was calculated from confocal images for TH-positive cells. The upper two panels show the TH, TUJ-1-positive cells in control subject (C) and patient-derived neurons (F). The middle two panels show the binary processed images (D and G), which were used as input for the calculation. The lower two panels show mitotracker staining in TH-positive cells (E and H). Mitochondrial volume fraction quantification from confocal imaging. Bars represent means SEM of triplicates from three independent experiments (I). The statistical significance was calculated from a one-way ANOVA; ^∗∗^p < 0.001. The scale bar in (C)–(H) represents 75 μM.

Since we observed a phenotype in all four patient lines, which in PARK2 may be related to mitochondrial dysfunction, we examined mitochondrial biology in more detail. We first determined mtDNA copy number in dopaminergic cultures from the PARK2 and control lines. No significant changes were seen in the amount of mitochondria DNA measured by qPCR against the nuclear DNA between the patient lines and the control or among the PARK2 lines (Figure 4A). Nor did we find an alteration in mitochondrial volume fraction, as determined by the ratio of mitochondria to cell volume in total cells (Figure 4B).

However, since our cultures consist of a mixed population of cells, we reexamined the cultures focusing solely on TH^+^ cells. This was done post hoc by double labeling cells for TH and TUJ-1 after live imaging of at least 600 cells from each control and patient line (Figures 4C–4H). MitoTracker staining (live) in TH^+^ (post hoc) neurons from the control line and a representative PARK2 line are shown in Figures 4E and 4H. As seen in Figure 4I, the fraction of mitochondria (MitoTracker; green) to cell volume (Calcein-AM; gray) calculated from confocal images constrained to TH^+^ neurons in all four PARK2 lines was significantly lower than in the control line. These results indicate that mutations in PARK2 contribute to changes in mitochondrial content in dopaminergic neurons in these cultures, but at this stage, no changes are seen in the other cell types present in the culture including astrocytes, NSC, and other midbrain neurons.

### Decreased Dopaminergic Differentiation and Mitochondrial Volume Ratio in Isogenic *PARK2* KO iPSC Lines

**Figure 5:**
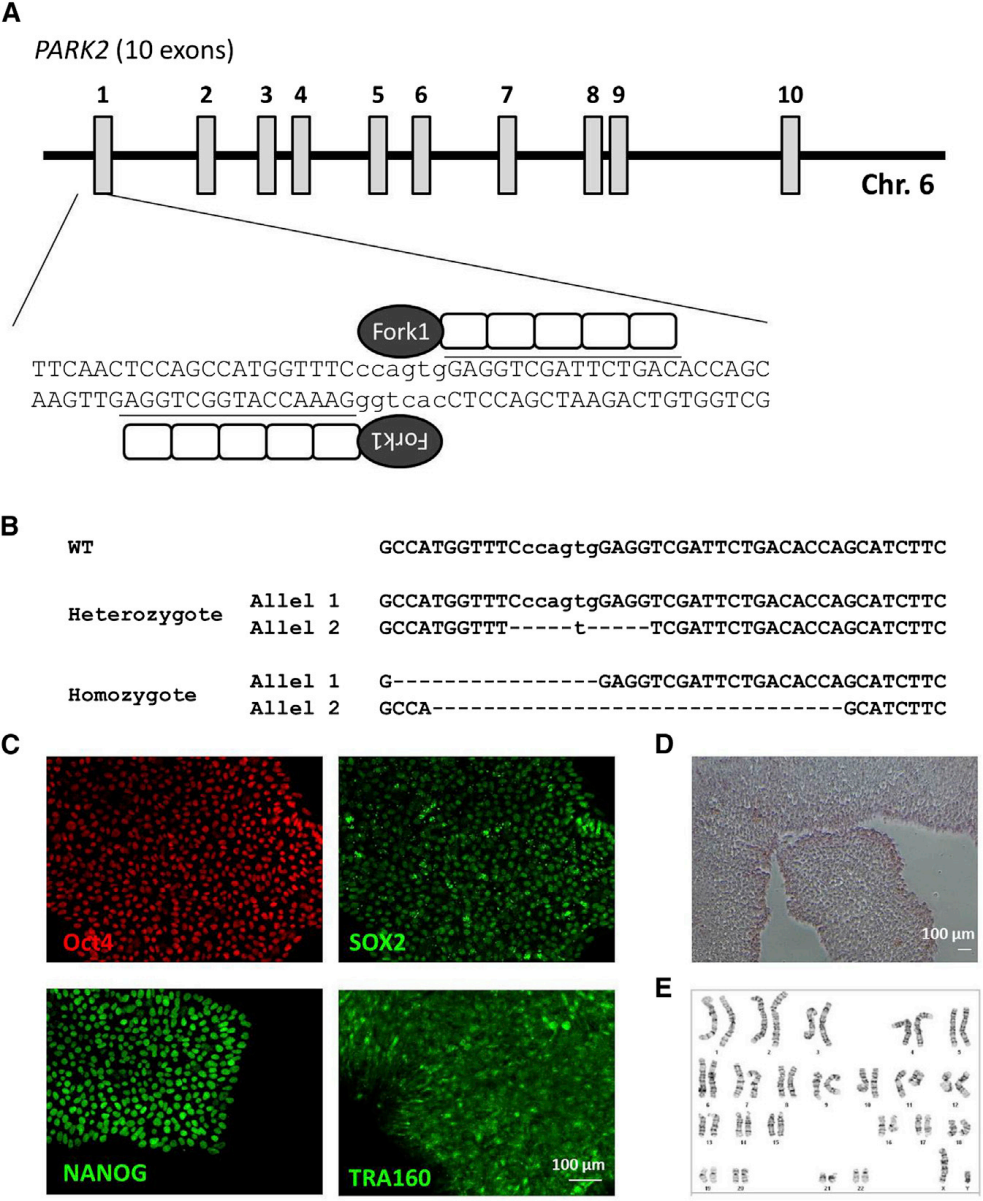
Generation of Isogenic *PARK2*^*−/−*^ iPSC Lines (A) Schematic representation of ZFNs binding to human *PARK2*. Each ZFN polypeptide consists of two functional domains: DNA binding domain (the recognition sequences of each ZFN are underlined) and the cleavage domain (FokI nuclease). (B) Mutations details of *PARK2* heterozygote (*PARK2*^*+/−*^) and homozygote (*PARK2*^*−/−*^) generated by ZFNs technology. WT sequences were shown in the top lane as reference. (C) *PARK2*^*−/−*^ iPSC maintained expression of pluripotency markers such as OCT4, SOX2, NANOG, and TRA1-60. (D) *PARK2*^*−/−*^ iPSC showed normal alkaline phosphatase staining. (E) *PARK2*^*−/−*^ iPSC showed a normal karyotype (46, XY).

**Figure 6:**
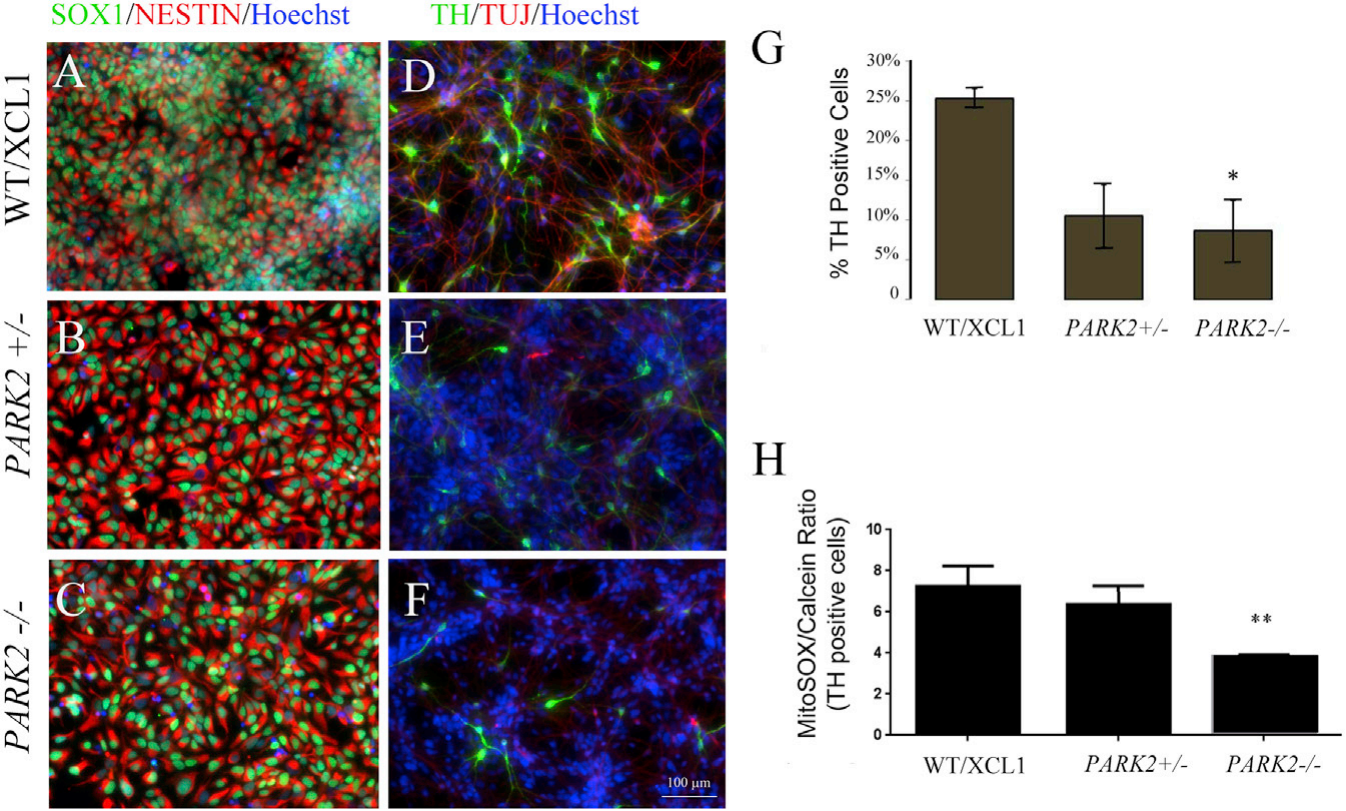
Decreased Dopaminergic Differentiation and Mitochondrial Abundance in Isogenic *PARK2*^*−/−*^ iPSC Lines (A–C) No detectable difference was observed in NSC uniformly expressing Nestin and Sox1 derived from WT, *PARK2*^*+/−*^ and *PARK2*^*−/−*^ iPSC lines. (D–F) Loss of dopaminergic neurons in *PARK2*^*+/−*^ and *PARK2*^*−/−*^ lines compared with the WT. Representative immunohistochemistry images of dopaminergic differentiated culture of *PARK2*^*+/−*^ and *PARK2*^*−/−*^ iPSC and their parental isogenic control line were stained for dopaminergic marker TH and TUJ-1. (G) Percent of TH-positive dopaminergic neurons *PARK2*^*+/−*^ and *PARK2*^*−/−*^ cells compared with their parental isogenic control WT line at day 28. (H) A significantly reduced mitochondrial volume fraction in TH-positive dopaminergic neurons was observed in the *PARK2*^*−/−*^ line. Bars represent means and SEM of four independent experiments. ^∗∗^p < 0.001 (Student’s t test). Scale bars represent 100 μM.

Although the results were compelling and consistent, the size of the cohort examined (four) is small, and the data do not allow us to conclude that PARK2 is sufficient to cause the observed phenotype. To address this issue, we obtained a set of isogenic iPSC lines mimicking the loss of function of *PARK2* gene created by Zinc Finger Nuclease (ZFN) in a well-characterized integration-free iPSC line XCL1 (XCell Science). Figures 5A and 5B showed a schematic representation of ZFN binding to *PARK2* and the frame-shift mutations introduced in the heterozygotes (*PARK2*^*+/−*^) and homozygote (*PARK2*^*−/−*^) used in this study. Both lines were validated for pluripotency and normal karyotypes (Figures 5C–5E). As expected, gene expression profiling did not show a phenotype at the iPSC stage. These data confirmed that the targeting process did not alter the line, and consistent with the patient line data, loss of PARK2 did not affect iPSC behavior. We next generated NSC from these isogenic lines (Figures 6A–6C). Consistent with earlier results, no difference in early neural differentiation was observed between the lines. Both NSC lines could differentiate into TH^+^ dopaminergic neurons (Figures 6D–6F). Quantification of the percentage of TH^+^ dopaminergic neurons revealed a significantly lower percentage in the *PARK2*^*−/−*^ line than in the WT control line (Figure 6G). We then examined the mtDNA copy number and mitochondrial volume fraction in TH^+^ dopaminergic neurons. No difference in DNA copy number or mitochondrial volume fraction in total cells in the mixed culture was found between the *PARK2*^*−/−*^ and its isogenic control (data not shown). However, similar to what we discovered in the patient-derived lines, mitochondrial volume fraction in TH^+^ neurons was significantly reduced in the *PARK2*^*−/−*^ line, but not in the *PARK2*^*−/+*^ line (Figure 6H).

This loss of *PARK2* was both necessary and sufficient to mimic the phenotype observed in the patient lines. To assess whether there were changes in mitochondrial biology that were large enough to be detected by microarray analysis, we prepared dopaminergic neurons from the WT and *PARK2*^*−/−*^ KO line and assessed the expression of ∼600 mitochondrial genes, mitophagy-related genes, genes known to be involved in cell death, and mitochondrial fission and fusion (Table S4). We reasoned that sensitivity should be higher in isogenic line comparison, and >2-fold changes may be enriched in genes that are biologically relevant genes. Consistent with our observation of a change in mitochondrial volume fraction in dopaminergic neurons, expression of several mitophagy-related genes was altered in the *PARK2*^*−/−*^-derived dopaminergic population. Twenty-five genes were seen to be 2-fold or higher expressed in the *PARK2*^*−/−*^ line, whereas 20 genes were expressed 2-fold or lower in the *PARK2*^*−/−*^ line (Table S5).

Overall, these data are consistent with our observation that *PARK2* null is a sensitive model of familial PARK2 patient-specific lines and can be used to assess the role of PARK2 in disease.

### Mitochondrial Ultrastructural Abnormalities in *PARK2*^*−/−*^ KO Line

To provide a more morphological assessment of the phenotype, we examined change of mitochondria in *PARK2*^*−/−*^ KO cells by electron microscopy. Cells from the isogenic control and the *PARK2*^*−/−*^ line were grown in parallel and differentiated into dopaminergic neurons. The mitochondrial morphology was examined in neurites and in cell soma using thin-cut electron microscopy sections. As a control, the normal cells were treated with rotenone at 100 μM for 24 hr, a well-characterized mitochondrial toxin, which induces characteristic changes in mitochondria that include changes in mitochondrial volume (Chauvin et al., 2001; Panov et al., 2005; Sherer et al., 2003a).

**Figure 7:**
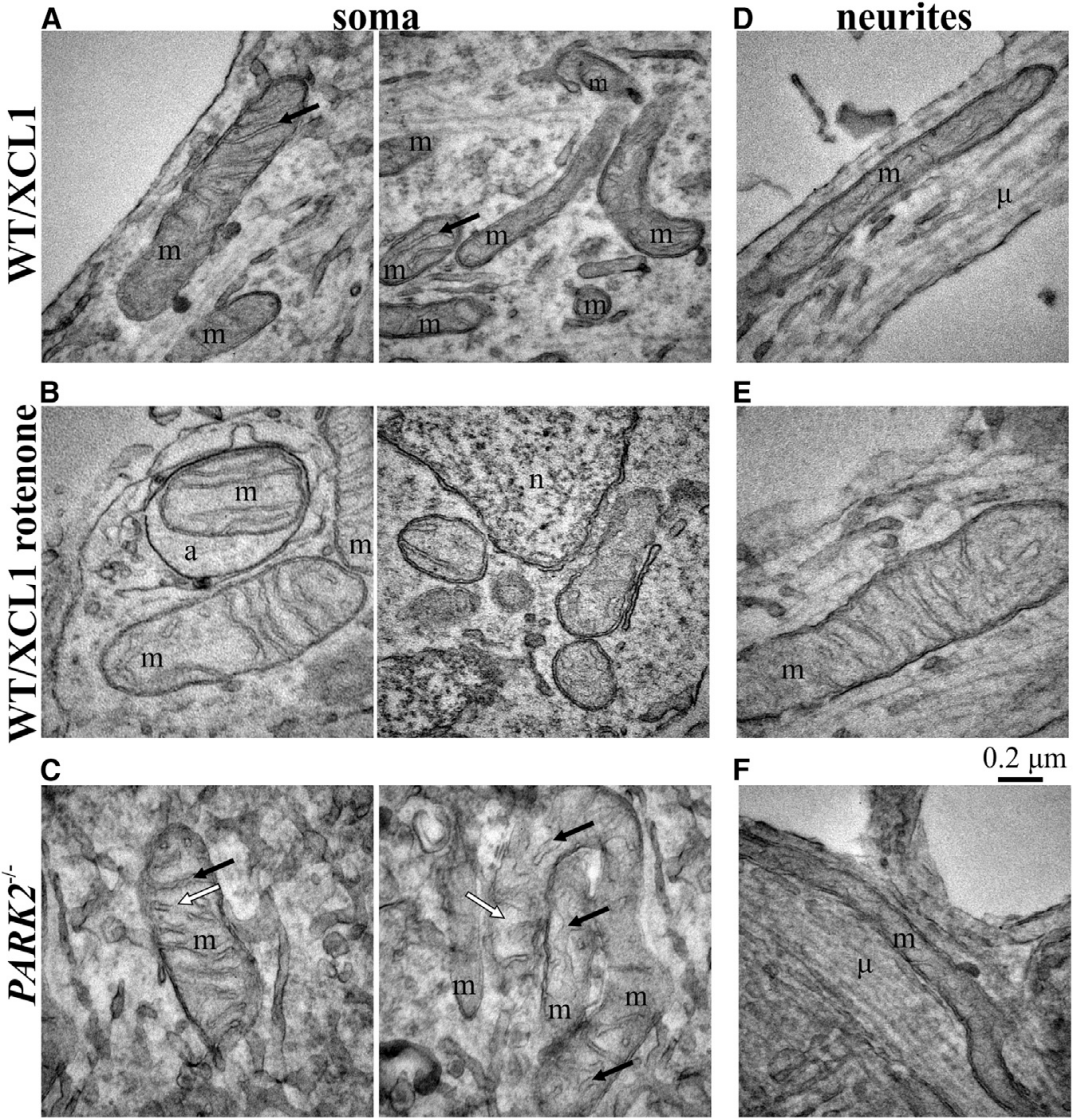
Mitochondrial Ultrastructure in Dopaminergic Neurons Transmission electronmicrographs of dopaminergic neuronal cultures were recorded at ×68,000 magnification in 70-nm thick sections. (A–C) Representative images of mitochondria (m) in neuronal somata. (D–F) Representative images of mitochondria in neurites identified by parallel organized microtubule tracts (μ) (A and D) WT dopaminergic neurons, (B and E) rotenone-treated (100 μM, 24 hr) dopaminergic neurons, and (C and F) *PARK2*^*−/−*^ neurons. Solid arrows point cristae of the inner mitochondrial membrane; open arrows mark the mitochondrial matrix. (E and F) (a), mitophagic vacuole; (n), nucleus. Results are representative of three independent experimental replicates.

As seen in Figures 7A and 7B, rotenone caused overt swelling and loss of matrix density in the WT control cultures. These ultrastructural alterations are similar to what has been previously described (Chauvin et al., 2001; Lin et al., 2012). As with WT cells treated with rotenone treatment, *PARK2*^*−/−*^ cells also showed similar changes in mitochondria present in the cell soma (Figure 7C). Mitochondria in *PARK2*^*−/−*^ neurons showed signs of swelling (decreased density of the mitochondrial matrix; Figure 7C, open arrows) and irregular, dilated cristae (arrows) as compared with the isogenic control. These differences were not observed in mitochondria present in neurites (Figures 7D–7F), suggesting a more limited mitochondrial damage. These data confirm and extend our observation that *PARK2* null neurons undergo stress in culture, which leads to mitochondrial damage and a slow progressive cell death.

### Altered Gene Expression of *PARK2*^*−/−*^ Neurons and Its Response to Stress

Since PARK2 is ubiquitously expressed, we reasoned that a subset of mitochondrial changes observed in dopaminergic cultures by microarray would likely be seen in other neuronal cell types. We therefore prepared a pure population of neurons from these isogenic lines using a protocol by which greater than 95% of the total cells at day 14 expressed TUJ-1 (Figure S2A) (Liu et al., 2013). We first examined whether the *PARK2*^*−/−*^ neurons were more stressed compared with the WT neurons. Morphologically, neuronal differentiation from both WT and *PARK2*^*−/−*^ lines is similar, and no alteration in cell lineage was detected (Figure S2B). Short-term survival in culture as assessed by 3-(4,5-dimethylthiazol-2-yl)-2,5-diphenyltetrazolium bromide (MTT) assay at days 8, 10, and 14 showed a gradual loss of cells in *PARK2*^*−/−*^ line as compared with the WT, as there was significant fewer neurons from the *PARK2*^*−/−*^ line by day 14 when an equal number of WT and *PARK2*^*−/−*^ cells were seeded at day 6 (Figure S2B), suggesting that loss of PARK2 function can cause cell death/stress.

We then performed a whole genome expression analysis on day 14 neurons of the isogenic lines and focused our analysis on alteration in expression of mitochondrial related genes. Ninety-five genes had altered expression by 2-fold; of them, 45 were upregulated, and 50 were downregulated in the mutant (Table S6). There was an increase in levels of a subset of mitochondrial genes and an upregulation of cell death genes. Of importance to note was the upregulation of SNCA in these cultures along with the upregulation of autophagy-related genes (Table S6). Of the *BCL2* family in the cell death gene dataset, *HARAKIRI* in particular appeared upregulated. Overall these results confirm the effect of *PARK2* mutations on mitochondria and are consistent with a mitochondrial abnormality inducing stress in cells, which leads to a BAD/BAX-mediated cell death.

## Discussion

Mutations in the *PARK2* gene are associated with PD, although the exact mechanism by which PARK2 contributes to the selective neuronal degeneration in PD is unknown. Different lines of evidence indicate that alterations in many aspects of mitochondrial biology such as complex I activity, fission and fusion, mitophagy, transport of mitochondria in neurons, and alterations mitochondrial membrane potential may contribute to PD (Dauer and Przedborski, 2003; Exner et al., 2012). Consistent with the mitochondrial hypothesis, it has been postulated that the role of PARK2 and PINK1 in mitochondrial quality control underlies the basis of PARK2-related PD. Our results showing an alteration in mitochondrial volume in *PARK2* mutants in a primary human dopaminergic cell model is consistent with this hypothesis. The deficits in mitochondrial volume were accompanied by a reduction in dopaminergic neurons in PARK2 patient lines, and these phenotypes were recapitulated in our isogenic *PARK2*^*−/−*^ lines. Whole genome expression profiling confirmed the phenotype and identified mitochondrial-associated cell death as a cause for the reduction in cell number.

Mitochondria play an important role in neuronal activity and survival. Neurons rely on oxidative phosphorylation for their energy supply, and the abundance of mitochondria is an important factor in determining survivability of neurons (Yadava and Nicholls, 2007). We have previously reported that differentiated neurons display higher mitochondrial biogenesis when compared with their early progenitors NSC (Birket et al., 2011). Misregulated biogenesis has been implicated to underlie pathological conditions in a number of neurodegenerative diseases. A host of proteins such as VDAC, cytochrome C, POLG, TFAM and PGC-1α, NRF-1 are known to regulate mitochondrial biogenesis, and differential expression of these proteins has been reported in various neurodegenerative disorders. Our array data did not show substantive differences in expression of these mitochondrial biogenesis genes between PARK2 patients and controls, indicating that the phenotype was not caused by mitochondrial biogenesis.

Deletion and overreplication of mtDNA are emerging as important factors underlying the selective loss of dopaminergic neurons during aging and in PD (Anderson et al., 1981; Ekstrand et al., 2007; Johns, 1995; Wei, 1998). Although we did not detect any changes in mtDNA copy numbers between healthy and diseased samples, the mitochondria-to-cell-volume fraction, an important parameter of mitochondrial membrane potential (Birket et al., 2011), was significantly reduced in PARK2 dopaminergic neurons. A decrease in mitochondrial membrane potential in PARK2-deficient cells could make a selective population of cells more vulnerable to stress stimuli. Consistent with this, PARK2 mutant *Drosophila* have been reported to accumulate depolarized mitochondria in dopaminergic neurons (Burman et al., 2012). Indeed, a decline mitochondrial membrane potential has been reported in PD patient derived fibroblasts, with PARK2 deficiency (Mortiboys et al., 2008).

Our results are consistent with previous reports of the action of PARK2 and reduction in TH-positive cells described in mouse *PARK2* KO model (Perier et al., 2013; Reeve et al., 2013; Rothfuss et al., 2009) and consistent with work done in the fly model. The mitochondrial phenotype was not seen when we examined the total cells in the culture; rather, it was only seen in TH-positive dopaminergic neurons, which represented only a small percentage of the total cells (<30%). This may explain the apparent discrepancy with an earlier report on two PARK2 iPSC lines when mtDNA copy number was determined in the mix culture (Jiang et al., 2012). These results also suggested that NSC, astrocytes, and other cell populations may not show a significant phenotype, and this was confirmed in our whole genome analysis of NSC and astrocyte samples (see Results; data not shown).

Of importance was our finding of changes in other PD-associated genes, including SNCA (Krüger et al., 1998; Polymeropoulos et al., 1997; Singleton et al., 2003; Zarranz et al., 2004). SNCA is a presynaptic protein and function in regulating synaptic vesicle and neurotransmitter release. Aggregates of SNCA have been identified within the cytoplasmic inclusions (Lewy bodies) along with PARK2 in the brains of PD patients. It has been suggested that PARK2-mediated ubiquitination regulates SNCA assembly into ubiquitin-positive cytosolic inclusions, lending support for absence of these inclusions in PD patients with *PARK2* mutation (Chung et al., 2001). Here, we report an increase in SNCA protein levels in patient-derived neurons concomitant with a decrease in TH-positive cells. Similar correlation was reported previously to be associated with the aging process (Chu and Kordower, 2007). Although, some cellular and tissue studies in PD patients argue against the excess of SNCA in pathogenesis of PD (Dächsel et al., 2007), recent studies reaffirm the increase in SNCA protein levels in iPSC-derived neurons from patient with *PARK2* mutation (Imaizumi et al., 2012). It is possible that this increase in SNCA expression is an early event in the disease process and that patients with a late stage of PD do not display this phenotype. We acknowledge that there are many differences between a cell culture model and what may be seen in a culture dish. One operating assumption is that loss of PARK2 may lead to reduced processing of SNCA-associated proteins in particular SYNPHILIN; other data suggest that it is a nonclassical pathway (Chung et al., 2001; Lim et al., 2005; Sherer et al., 2003b; Zhang et al., 2013). We believe that altered ratios of interacting proteins may lead to either an increase or decrease depending on the stage of the disease. However, it is difficult to mimic the exact disease stage in culture just as it has been hard to do so in rodents in vivo. Nevertheless, our data were consistent in all PARK2 patient lines in vitro.

We did not examine the presence of inclusion bodies or association of SNCA with these aggregates. In our culture, we did not observe a selective expression of SNCA in TH-positive cells, but rather, the expression was more random. In the absence of an appropriate reporter line, we could not infer from our data whether the TH-positive and SNCA-negative cells are surviving cells or that SNCA upregulation is delayed in these cells.

PARK2 is a ubiquitously expressed protein, and its ubiquitination of outer mitochondrial membrane is a prerequisite step in mitophagy-mediated removal of damaged mitochondria. However, PARK2 abnormalities in cells other than neurons fail to display the selective loss of a particular population of cells, suggesting that dysfunctional mitophagy could be compensated or delayed. Both PD patients and *PARK2* KO dopaminergic neurons display upregulation of several key mitophagy-associated proteins, as determined by our array data. Similarly, in our isogenic lines, the expression of these mitophagy-related genes displayed alleleic dependency and stage specificity. We identified a number cell death-inducing genes that were upregulated in dopaminergic neurons derived from PARK2 patients and *PARK2* KO lines; these include BID, BAX, BIM, BAK, PUMA, NOXA, BNIP3, and NIK (BCL-2 interacting killer). Although the mechanism by which dysfunctional mitophagy contribute to PD pathogenesis remain to be investigated, here we show that for the first time that PARK2 contributes to mitochondrial mass (volume) in dopaminergic neurons. We show that TH-positive neurons in PD patient and *PARK2* KO lines have a reduced mitochondrial mass compared with controls. A decrease in population of mitochondria within these TH-positive cells would shift the balance between healthy and defective mitochondrial and render these cells more vulnerable to accumulation of damaged mitochondria. We did not observe this alteration in the absence of *PARK2* mutations.

Given the ubiquitous expression of PARK2 and the changes we observed in our mixed dopaminergic neuron cultures, as well as previously published reports of observable phenotypes in cell lines unrelated to neurons (da Costa et al., 2009; Tsai et al., 2003), we reasoned that a subset of these changes may be seen in other neurons other than dopaminergic neurons. We took advantage of a neuronal differentiation system that we have developed (Liu et al., 2013) and examined a pure population of neurons of *PARK2* mutants. We focused our analysis on isogenic PARK2 lines as a more sensitive model of the PARK2 phenotype. Similar changes were seen, as with dopaminergic neurons. We saw a gradual decline in the number of surviving neurons in culture to approximately half of that in the isogenic control sample. This phenotypic change was consistent with previous observations in mouse models, which showed a decreased survival in response to stress (Sherer et al., 2003b; Testa et al., 2005). Comparison of the mitochondrial and cell death gene changes showed a similar but not identical profile. These results along with the lack of a phenotype in iPSC, NSC, and astrocytes highlight the importance of studying the effect in an appropriate cellular context. Our observation that the phenotype can be studied in generic neurons provides a feasible assay with additional stress using a purified population of cells that can be obtained 2- to 3-fold faster and with much less effort than authentic midbrain dopaminergic neurons.

Overall, our results provide a current model of PARK2 function where damaged mitochondria are targeted for degradation via a PARK2/PINK1 interaction. Loss of PARK2 results in an initial accumulation of damaged mitochondria, and stress in culture results in a slow reduction in cell number as internal repair process fail to compensate for loss of metabolic activity. Cells with a higher metabolic activity are more susceptible to suffer mitochondrial loss and display a phenotype earlier than more robust glial cells. Using multiple lines and generating isogenic controls and combining phenotypic changes with gene expression profiling provide a useful model for elucidating pathways underlying the disease process and provide important tools that are useful for the PD community. These results also suggest that the engineered PARK2 KO and it isogenic controls provide a valuable model to assess familial PD models and to construct single- and double-mutant models.

## Experimental Procedures

### Generation of Patient-Specific and Isogenic iPSC Lines

PD patient fibroblasts were obtained from Coriell. Fibroblasts growing conditions, reprogramming, and differentiation procedures are described in the Supplemental Information.

The isogenic *PARK2* KO lines were generated by ZFN technology and were obtained from XCell Science. The detailed method was described in the Supplemental Information.

### Neural and Dopaminergic Neuronal Differentiation

Generation of NSC and dopaminergic differentiation from NSC was described (Swistowski et al., 2009). The detailed procedures were seen in the Supplemental Information.

### Immunocytochemistry and Western Blot

Immunocytochemistry and western blot procedures were as described previously (Zeng et al., 2003). See Supplemental Information for used antibodies and further descriptions.

### Microarray and qPCR Analyses

Total RNA was isolated using the RNeasy Mini kit according to the manufacturer’s instructions (QIAGEN) and hybridized to Illumina Human HT-12 BeadChip (Illumina, performed by Microarray core facility at the Burnham Institute for Medical Research). All of the data processing and analysis were performed using the algorithms included with the Illumina BeadStudio software, and further description can be found in Supplemental Information. The qPCR procedure was described in the Supplemental Information. The GEO accession number for microarray data is GSE66241.

### MtDNA Copy Number Assessment

The mtDNA copy number was determined by comparing PCR amplification of a mitochondrial amplicon (human, NADH-ubiquinone oxidoreductase chain 5 [ND5]) with a nuclear amplicon (human, cystic fibrosis) (Wong and Cortopassi, 2002). The standard curves were generated for quantifications obtained by amplification curves of nuclear cystic fibrosis gene and ND5 gene amplified from 0- to 100-ng and 0- to 1-ng K562 DNA, respectively.

### Confocal Microscopic Stereology of Mitochondria Volume Fraction

Mitochondria: cell volume fractions (V_F_) were determined using a confocal microscopy and image processing-based stereologic approach according to (Gerencser et al., 2012) and are further described in Supplemental Information.

### Electron Microscopy

Transmission electron microscopy was performed as previously described (Birket et al., 2011), and the detail was described in the Supplemental Information.

### Statistical Analysis

Statistical analyses were performed using two-tailed paired or unpaired when analyzing isogenic lines. For patient-derived cell lines, the statistical significance was calculated from a one way ANOVA using Dunnett’s correction. ^∗^p < 0.05, ^∗∗^p < 0.01.

## Author Contributions

A.S. performed differentiation and mitochondrial function experiments. R.S. generated some patient iPSC lines and performed microarray analysis. Y.P. generated some isogenic Park2 iPSC lines.

## Accession Numbers

The microarray data utilized in this study were deposited under the GEO accession number GSE66241.

## Supplemental Information

Document S1. Supplemental Experimental Procedures, Figures S1 and S2, and Tables S1–S6

Document S2. Article plus Supplemental Information
