# Supplementary material for: Mitochondrial Alterations by PARKIN in Dopaminergic Neurons Using PARK2 Patient-Specific and PARK2 Knockout Isogenic iPSC Lines
Source: Stem Cell Reports. 2015 Apr 2;4(5):847–59. doi: 10.1016/j.stemcr.2015.02.019 (PMC4437475; doi:10.1016/j.stemcr.2015.02.019)
Supplement: Document S1. Supplemental Experimental Procedures, Figures S1 and S2, and Tables S1–S6 [file mmc1.pdf]

Stem Cell Reports, Volume *4*

Supplemental Information

Mitochondrial Alterations by PARKIN in Dopaminergic Neurons Using PARK2 Patient-Specific and *PARK2* Knockout Isogenic iPSC Lines

Atossa Shaltouki, Renuka Sivapatham, Ying Pei, Akos A. Gerencser, Olga Momčilović, Mahendra S. Rao, and Xianmin Zeng

Supplemental Figure 1.

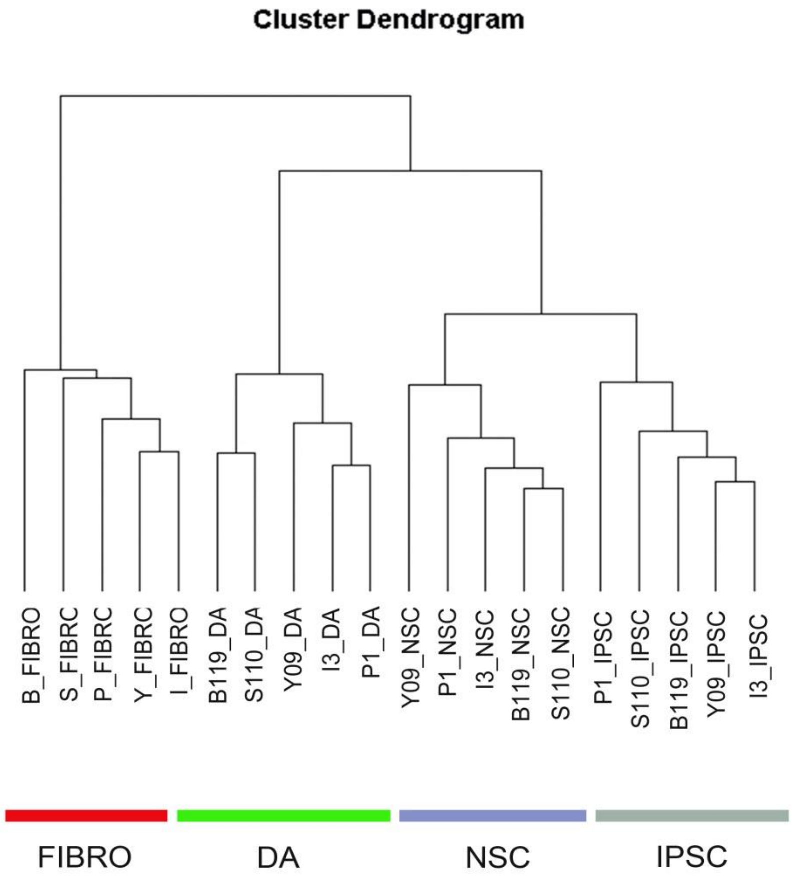

Sup Figure 1. Whole gene expression profiles of PARK2 and control lines at various stages of dopaminergic differentiation. The dendrogram confirms the clustering among iPSC lines, iPSC-derived NSC and iPSC-derived dopaminergic neurons. The horizontal axis of the dendrogram represents the dissimilarity between clusters in terms of gene expression pattern. The vertical axis represents clusters. Abbreviations: F: Fibroblast; iPSC: Induced pluripotent stem cells; NSC: neural stem cells; DA: dopaminergic neurons.

Supplemental Figure 2.

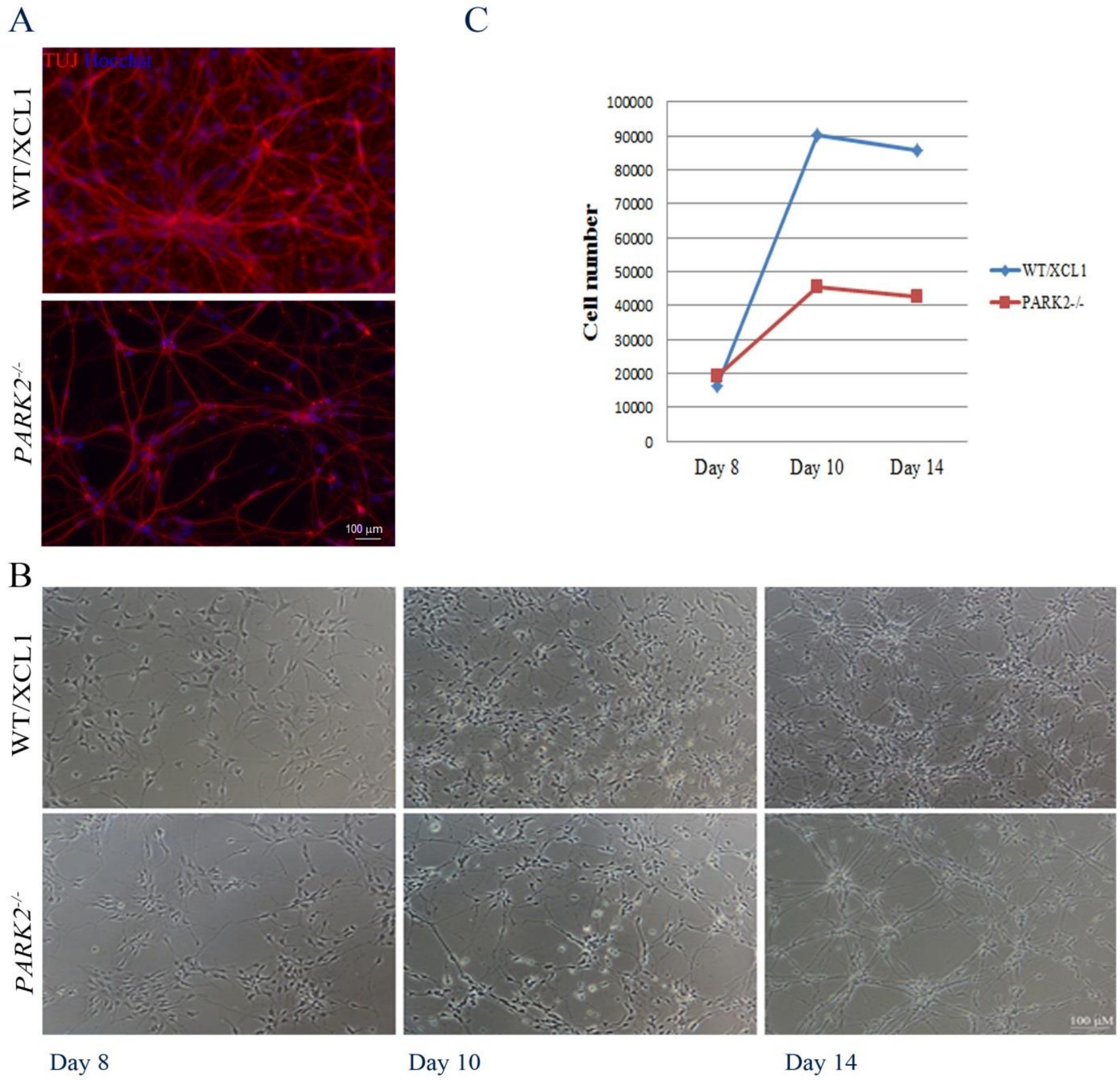

Sup Figure 2. *PARK2*$^{-/-}$ neurons appeared to be more stressed compared to their isogenic control neurons. (A) Generation of a pure population of neurons from the *PARK2* isogenic lines. More than 95% of total cells expressed TUJ-1 after 14 days of differentiation (time of assay) in both WT and *PARK2*$^{-/-}$ lines. (B) Morphology of neuronal cells at Day 8, 10 and 14 of Park2 KO and the control WT cells. (C) Neuronal cell count at various time points. WT cell showed a higher rate of cell proliferation when

compared to control *PARK2*$^{-/-}$ measured by the MTT assay. Results are representative of three independent experiments.

Supplemental Table 1. List of PARK2 patient and control cells used in the study.

Line	NINDS Catalog ID	Mutation	Gender	Race	Age of onset	Age of sample
PARK2	ND30171 (P)	Park2: R42P<br>Park2: EX3DEL	Male	Caucasian	42	54
	ND29543 (I)	Park2: EX3-4DEL<br>Park2: 1-BP DEL, 255A	Male	Hispanic	16	50
	ND29369 (B)	Park2: R275W	Female	Hispanic	43	61
	ND31618 (S)	Park2: R42P	Female	Caucasian	44	63
Control	ND34791 (Y)	Population control	Female	Caucasian	n/a	60

Supplemental Table 2. Expression of PD genes in Fibroblast and iPSC patient lines.

SYMBOL	P Fibro	I Fibro	B Fibro	S Fibro	Y Fibro	P1 iPSC	I3 iPSC	B119 iPSC	S110 iPSC	Y9 iPSC
ATP13A2	384	270	245	249	303	172	304	334	411	178
ATXN2	4175	3345	3041	3396	4208	2309	3897	3112	2584	3785
BST1	691	884	169	323	281	-2	25	30	5	-5
EIF4G1	2725	1331	1458	1188	2039	1534	1235	1389	1577	1212
FBXO7	479	602	709	734	516	305	459	465	490	458
FGF20	20	23	18	17	28	35	4	55	40	31
GAK	867	472	571	493	576	506	502	401	391	630
GBA	1738	1759	1465	2907	2013	361	533	271	540	380
GIGYF2	14	40	27	11	47	38	51	34	32	47
GPNMB	642	1030	790	1177	1312	80	6	71	20	33
HTRA2	666	620	786	780	581	309	296	430	300	348
MC1R	464	652	646	441	375	287	117	106	177	164
MCCC1	689	753	641	828	618	1163	967	989	998	1113
PARK7	15260	16223	16048	15681	15538	15594	16395	19195	16467	17639
PDXK	2332	1455	1645	1621	1709	2091	1386	1662	1323	1253
PINK1	1925	1789	1619	1967	1419	231	364	265	464	414
PM20D1	42	66	43	74	51	64	55	32	10	28
RAB25	-10	35	8	20	30	846	1508	1722	1825	2147
SETD1A	786	420	595	397	434	413	573	714	529	573
SNCA	74	96	37	115	76	676	617	1099	872	1129
STK39	1800	2220	2393	1542	1958	1455	1793	2208	2103	1750
TBP	758	794	948	762	795	1198	1174	1413	1190	1182
UCHL1	740	3420	2045	3740	3359	15075	19112	19483	17539	15232
VPS35	3434	3816	4161	4053	3465	2728	3083	2005	2271	2841

Supplemental Table 3. R^2 of all patient line.

R^2	Y FIBRO	Y9 IPSC	Y09 NSC	Y09 DA2	B FIBRO	B119 IPSC	B119 NSC	B119 DA2	I FIBRO	I3 IPSC	I3 NSC	I3 DA2	P FIBRO	P1 IPSC	P1 NSC	P1 DA2	S FIBRO	S110 IPSC	S110 NSC	S110 DA2
Y FIBRO	1	0.86	0.88	0.84	0.96	0.86	0.86	0.8	0.98	0.85	0.86	0.86	0.97	0.86	0.87	0.85	0.97	0.86	0.87	0.82
Y9 IPSC	0.86	1	0.95	0.91	0.88	0.98	0.95	0.86	0.86	0.99	0.95	0.92	0.83	0.97	0.95	0.92	0.86	0.98	0.95	0.89
Y09 NSC	0.88	0.95	1	0.94	0.9	0.95	0.97	0.89	0.89	0.94	0.97	0.94	0.86	0.93	0.96	0.94	0.88	0.94	0.97	0.92
Y09 DA2	0.84	0.91	0.94	1	0.85	0.91	0.93	0.96	0.85	0.9	0.94	0.98	0.8	0.89	0.93	0.97	0.84	0.9	0.93	0.97
B FIBRO	0.96	0.88	0.9	0.85	1	0.88	0.89	0.82	0.97	0.87	0.89	0.87	0.96	0.86	0.89	0.86	0.96	0.88	0.89	0.84
B119 IPSC	0.86	0.98	0.95	0.91	0.88	1	0.96	0.86	0.87	0.98	0.96	0.92	0.83	0.97	0.95	0.92	0.86	0.98	0.96	0.89
B119 NSC	0.86	0.95	0.97	0.93	0.89	0.96	1	0.9	0.87	0.94	0.99	0.95	0.83	0.93	0.98	0.95	0.86	0.95	0.99	0.93
B119 DA2	0.8	0.86	0.89	0.96	0.82	0.86	0.9	1	0.8	0.86	0.91	0.96	0.78	0.84	0.89	0.96	0.79	0.87	0.9	0.98
I FIBRO	0.98	0.86	0.89	0.85	0.97	0.87	0.87	0.8	1	0.86	0.87	0.86	0.98	0.85	0.87	0.85	0.98	0.86	0.87	0.82
I3 IPSC	0.85	0.99	0.94	0.9	0.87	0.98	0.94	0.86	0.86	1	0.95	0.92	0.83	0.98	0.94	0.91	0.85	0.98	0.95	0.89
I3 NSC	0.86	0.95	0.97	0.94	0.89	0.96	0.99	0.91	0.87	0.95	1	0.96	0.83	0.94	0.98	0.96	0.86	0.95	0.99	0.94
I3 DA2	0.86	0.92	0.94	0.98	0.87	0.92	0.95	0.96	0.86	0.92	0.96	1	0.83	0.91	0.95	0.99	0.85	0.92	0.95	0.98
P FIBRO	0.97	0.83	0.86	0.8	0.96	0.83	0.83	0.78	0.98	0.83	0.83	0.83	1	0.82	0.83	0.81	0.96	0.84	0.84	0.8
P1 IPSC	0.86	0.97	0.93	0.89	0.86	0.97	0.93	0.84	0.85	0.98	0.94	0.91	0.82	1	0.95	0.91	0.84	0.96	0.94	0.87
P1 NSC	0.87	0.95	0.96	0.93	0.89	0.95	0.98	0.89	0.87	0.94	0.98	0.95	0.83	0.95	1	0.95	0.86	0.94	0.98	0.92
P1 DA2	0.85	0.92	0.94	0.97	0.86	0.92	0.95	0.96	0.85	0.91	0.96	0.99	0.81	0.91	0.95	1	0.84	0.91	0.95	0.98
S FIBRO	0.97	0.86	0.88	0.84	0.96	0.86	0.86	0.79	0.98	0.85	0.86	0.85	0.96	0.84	0.86	0.84	1	0.85	0.86	0.82
S110 IPSC	0.86	0.98	0.94	0.9	0.88	0.98	0.95	0.87	0.86	0.98	0.95	0.92	0.84	0.96	0.94	0.91	0.85	1	0.96	0.9
S110 NSC	0.87	0.95	0.97	0.93	0.89	0.96	0.99	0.9	0.87	0.95	0.99	0.95	0.84	0.94	0.98	0.95	0.86	0.96	1	0.93
S110 DA2	0.82	0.89	0.92	0.97	0.84	0.89	0.93	0.98	0.82	0.89	0.94	0.98	0.8	0.87	0.92	0.98	0.82	0.9	0.93	1

Supplemental Table 4. Mitochondrial and cell death-related genes.

Mitochondria genes

ND1	*NDUFB1*	*CO2*	*COX7B2*	*ATP1B4*	*ATP5J*	*ATP6V1E1*	*UQCC*
ND2	*NDUFB10*	*C03*	*COX7C*	*ATP2A1*	*ATP5J2*	*ATP6V1E2*	*UQCR*
ND3	*NDUFB11*	*COX10*	*COX8A*	*ATP2A2*	*ATP5L*	*ATP6V1F*	*UQCRB*
ND4	*NDUFB2*	*COX11*	*COX8C*	*ATP2A3*	*ATP5O*	*ATP6V1G1*	*UQCRC1*
ND4L	*NDUFB3*	*COX11P*	*MT-ATP6*	*ATP2B1*	*ATP5S*	*ATP6V1G2*	*UQCRC2*
ND5	*NDUFB4*	*COX15*	*MT-ATP8*	*ATP2B2*	*ATP5SL*	*ATP6V1G3*	*UQCRFS1*
ND6	*NDUFB5*	*COX16*	*ATP10A*	*ATP2B3*	*ATP6AP1*	*ATP6V1H*	*UQCRH*
NDUFA1	*NDUFB6*	*COX17*	*ATP10B*	*ATP2B4*	*ATP6AP1L*	*ATP7A*	*UQCRHL*
NDUFA10	*NDUFB7*	*COX18*	*ATP10D*	*ATP2C1*	*ATP6AP2*	*ATP7B*	*UQCRQ*
NDUFA11	*NDUFB8*	*COX19*	*ATP11A*	*ATP2C2*	*ATP6V0A1*	*ATP8A1*	*CYC1*
NDUFA12	*NDUFB9*	*COX4I1*	*ATP11B*	*ATP4A*	*ATP6V0A2*	*ATP8A2*	*SDHA*
NDUFA13	*NDUFC1*	*COX4I2*	*ATP11C*	*ATP4B*	*ATP6V0A4*	*ATP8B1*	*SDHAF1*
NDUFA2	*NDUFC2*	*COX4NB*	*ATP12A*	*ATP5A1*	*ATP6V0B*	*ATP8B2*	*SDHAF2*
NDUFA3	*NDUFS1*	*COX5A*	*ATP13A1*	*ATP5B*	*ATP6V0C*	*ATP8B3*	*SDHALP1*
NDUFA4	*NDUFS2*	*COX5B*	*ATP13A2*	*ATP5C1*	*ATP6V0D1*	*ATP8B4*	*SDHAP2*
NDUFA4L2	*NDUFS3*	*COX6A1*	*ATP13A3*	*ATP5D*	*ATP6V0D2*	*ATP9A*	*SDHAP3*
NDUFA5	*NDUFS4*	*COX6A2*	*ATP13A4*	*ATP5E*	*ATP6V0E1*	*ATP9B*	*SDHB*
NDUFA6	*NDUFS5*	*COX6B1*	*ATP13A5*	*ATP5EP2*	*ATP6V0E2*	*ATPAF1*	*SDHC*
NDUFA7	*NDUFS6*	*COX6B2*	*ATP1A1*	*ATP5F1*	*ATP6V1A*	*ATPAF2*	*SDHD*
NDUFA8	*NDUFS7*	*COX6BP1*	*ATP1A2*	*ATP5G1*	*ATP6V1B1*	*ATPBD1B*	*GSK3A*
NDUFA9	*NDUFS8*	*COX6C*	*ATP1A3*	*ATP5G2*	*ATP6V1B2*	*ATPBD3*	*GSK3B*
NDUFAB1	*NDUFV1*	*COX7A1*	*ATP1A4*	*ATP5G3*	*ATP6V1C1*	*ATPBD4*	*UCP1*
NDUFAF1	*NDUFV2*	*COX7A2*	*ATP1B1*	*ATP5H*	*ATP6V1C2*	*ATPGD1*	*UCP2*
NDUFAF2	*NDUFV3*	*COX7A2L*	*ATP1B2*	*ATP5I*	*ATP6V1D*	*ATPIF1*	*UCP3*
NDUFAF3	*CO1*	*COX7B*	*ATP1B3*				

Death genes

SLC25A31	*BCL7B*	*PARK7*	*ATP6V0A4*	*ATG4B*	*GCM1*	*H2AFX*	*SNORD25*
SLC25A4	*BCL7C*	*PDDC1*	*ATP6V0B*	*LOC644284*	*SNORD48*	*LOC729057*	*SDK2*
SLC25A5	*BCL8*	*PACRG*	*ATP6V0C*	*NAMPT*	*LOC650034*	*RPL12P6*	*STT3B*
SLC25A6	*BCL9*	*PACRGL*	*ATP6V0D1*	*LOC654201*	*RNU1F1*	*LOC728787*	*SRCAP*
PPID	*BCL9L*	*PINK1*	*ATP6V0D2*	*LOC649841*	*ISLR2*	*LOC645195*	*LOC100129685*
VCY1B	*BCLAF1*	*SNCA*	*ATP6V0E1*	*LOC653383*	*CDK5R2*	*TBX19*	*SUV420H1*
VDAC1	*HRK*	*SNCAIP*	*ATP6V0E2*	*OXT*	*RN7SK*	*LOC729926*	*NSUN5B*
VDAC2	*BID*	*SNCB*	*ATP6V1A*	*SNORD95*	*GNAQ*	*SNORA80*	*NGFR*
VDAC3	*BAD*	*SNCG*	*ATP6V1B1*	*RNU1G2*	*CACYBP*	*RAB11B*	*DPF1*
AVEN	*BAG1*	*LRRK2*	*ATP6V1B2*	*RNU1-3*	*RNU4-2*	*HS.545589*	*SCAND1*
CARD10	*BAG2*	*UCHL1*	*ATP6V1C1*	*RNU1-5*	*INPP5D*	*IL12A*	*RNU6-1*
CARD11	*BAG3*	*NR4A2*	*ATP6V1C2*	*LOC100130562*	*LOC642255*	*SNORD12C*	*LOC440258*
CARD14	*BAG4*	*ATP13A2*	*ATP6V1D*	*HS.537779*	*HS.582113*	*LOC642661*	*LOC100134364*
CARD16	*BAG5*	*AMBRA1*	*ATP6V1E1*	*DKFZP547K054*	*FOXS1*	*LOC100134468*	*TBX2*
CARD17	*TFAM*	*BECN1*	*ATP6V1E2*	*KSR1*	*RN5S9*	*OSAP*	*PRKAR1B*
CARD18	*TFAMP1*	*BECN1L1*	*ATP6V1F*	*LOC442041*	*ELAVL2*	*FSD1L*	*SESN3*
CARD6	*PPARG*	*BLOC1S1*	*ATP6V1G1*	*PSMC4*	*DRD3*	*GADD45A*	*POGZ*
CARD8	*PPARGC1A*	*BLOC1S2*	*ATP6V1G2*	*LOC139116*	*WASH3P*	*CDKN1A*	*RNU6-15*
CARD9	*PPARGC1B*	*BLOC1S3*	*ATP6V1G3*	*CNTD2*	*FADS2*	*SNORD13*	*NOVA2*
CASKIN2	*POLRMT*	*LAMP1*	*ATP6V1H*	*HS.564389*	*PPDPF*	*HS.562219*	*LOC389672*
CASP1	*MTERF*	*LAMP2*	*MMP1*	*LOC399942*	*LSM11*	*C7ORF20*	*LOC642962*
CASP10	*MTERFD1*	*LAMP3*	*MMP10*	*SNORA84*	*C17ORF89*	*HS.133410*	*BLOC1S2*
CASP12	*MTERFD2*	*LAPTM4A*	*MMP11*	*LOC641901*	*CDC2L1*	*SFXN1*	*RBM18*
CASP14	*MTERFD3*	*LAPTM4B*	*MMP12*	*SNORA28*	*LOC646330*	*LGALS3*	*CDC42EP1*
CASP2	*GABPAP*	*LAPTM5*	*MMP13*	*LOC730167*	*ALOX5AP*	*SLC25A36*	*GUCY1A2*
CASP3	*GABPB1*	*LYST*	*MMP14*	*RNU1A3*	*SNORD3D*	*FOSB*	*ZBTB11*
CASP4	*GABPB2*	*NPC1*	*MMP15*	*LOC100132564*	*LOC652255*	*LOC158301*	*HIP1R*
CASP5	*KEAP1*	*NPC1L1*	*MMP16*	*LOC100132394*	*HRK*	*PI4KA*	*RPPH1*

CASP6	*NFE2*	*NPC2*	*MMP17*	*LOC652826*	*KCNJ4*	*HMOX1*	*LOC347544*
CASP7	*NFE2L1*	*EPM2A*	*MMP19*	*LOC389049*	*LOC440311*	*FLCN*	*TMEM8B*
CASP8	*NFE2L2*	*EPM2AIP1*	*MMP2*	*LOC441193*	*LOC653156*	*LOC730995*	*LOC100008588*
CASP8AP2	*NFE2L3*	*ATG10*	*MMP20*	*C1QTNF4*	*ISG20*	*CCRN4L*	*CDC34*
CASP9	*ATF1*	*ATG12*	*MMP21*	*RRAD*	*LOC643446*	*FAM46C*	*LOC100130276*
LOC650759	*ATF2*	*ATG16L1*	*MMP23A*	*LOC100131017*	*LOC728188*	*EPHA2*	*SNORD36A*
MCL1	*ATF3*	*ATG16L2*	*MMP23B*	*HIST1H2BJ*	*HERC5*	*CTSD*	*TCEB3*
BCL10	*ATF4*	*ATG2A*	*MMP24*	*LHX5*	*TNKS*	*LOC648931*	*C1ORF70*
BCL11A	*ATF5*	*ATG2B*	*MMP25*	*LOC100131323*	*PER1*	*LOC100130835*	*IER5L*
BCL11B	*ATF6*	*ATG3*	*MMP26*	*FAM108A3*	*UBTD1*	*PSMA7*	*WWP2*
BCL2	*ATF6B*	*ATG4A*	*MMP27*	*SPTBN1*	*PLK3*	*HIST3H2BB*	*RNU6ATAC*
BCL2A1	*ATF7*	*ATG4B*	*MMP28*	*TNFRSF12A*	*HSPBP1*	*RALGAPB*	*ITGA5*
BCL2L1	*ATF7IP*	*ATG4C*	*MMP3*	*GABPB1*	*RBM38*	*SH2B2*	*LOC727980*
BCL2L10	*ATF7IP2*	*ATG4D*	*MMP7*	*RNU11*	*SIRT7*	*HIST1H4E*	*NFKBIL1*
BCL2L11	*SIRT3*	*ATG5*	*MMP8*	*RELA*	*RAB30*	*LOC100134424*	*RARA*
BCL2L12	*PRKAA1*	*ATG7*	*MMP9*	*SNORD55*	*SDC4*	*UNCX*	*HSPA1B*
BCL2L13	*PRKAA2*	*ATG9A*	*MMPL1*	*LOC651149*	*RNU4ATAC*	*AKT1S1*	*SPIN1*
BCL2L14	*GCN1L1*	*ATG9B*	*LRRC4B*	*VTRNA1-2*	*PI4KAP1*	*TNRC4*	*C6ORF221*
BCL2L15	*MTFR1*	*NEU1*	*SDR39U1*	*DLG4*	*LOC345630*	*NSUN5C*	*ZNF787*
BCL2L2	*MFN1*	*SMPD1*	*BRAF*	*HSPA7*	*WASH5P*	*ZNF570*	*NOC2L*
BCL3	*MFN2*	*ATP6AP1*	*LOC100008589*	*SNORD3A*	*PMAIP1*	*NPAS4*	*NUCKS1*
BCL6	*OPA1*	*ATP6AP1L*	*LOC100133719*	*DCC*	*TRAF4*	*NACC2*	*BTG3*
BCL6B	*CRLS1*	*ATP6AP2*	*GIT2*	*KDM6B*	*CRYAB*	*CSNK2A1P*	*TCEA1*
BCL7A	*CRMP1*	*ATP6V0A1*	*SNORA63*	*RRN3P2*	*LOC100133950*	*RHOF*	*LOC346950*
	PARK2	*ATP6V0A2*					

Supplemental Table 5. Differential expression of mitochondria related genes in dopaminergic neurons derived from WT and *PARK2* $^{-/-}$ transgenic iPSC lines.

SYMBOL	*PARK2*$^{-/-}$ DA2	*PARK2-/+* DA2	WT DA2	*PARK2*$^{-/-}$ / WT DA2 Fold increase
NR4A2	984	205	172	5.71
NPAS4	202	217	39	5.24
COX11P	52	59	11	4.95
C1QTNF4	941	2267	218	4.31
NOVA2	550	858	135	4.09
ATF7IP	72	33	20	3.67
DPF1	2744	4382	874	3.14
LOC441193	42	13	14	2.95
TFAMP1	61	41	21	2.91
UCP3	94	67	36	2.62
LSM11	189	460	73	2.58
RARA	2218	1459	884	2.51
CDK5R2	455	1023	189	2.40
PDDC1	731	784	308	2.37
NDUFB1	224	331	97	2.31
SNCB	103	517	45	2.30
BLOC1S1	2388	1824	1043	2.29

SCAND1	6180	6422	2713	2.28
ISLR2	6080	1450	2742	2.22
WASH5P	305	363	139	2.20
ATP5D	9074	10479	4175	2.17
ATG16L2	151	121	70	2.17
BCL11B	2704	5161	1271	2.13
PRKAR1B	442	524	209	2.12
NDUFB7	12786	10745	6145	2.08

SYMBOL	***PARK2***$^{-/-}$ **DA2**	***PARK2***$^{-/+}$ **DA2**	**WT DA2**	***PARK2***$^{-/-}$ **/ WT DA2 Fold decrease**
CDKN1A	3956	4111	11598	0.34
ATF5	766	1037	3556	0.22
NEU1	779	959	2399	0.32
CRYAB	192	65	2005	0.10
ITGA5	185	247	1931	0.10
LAMP3	175	156	1559	0.11
HERC5	232	228	1262	0.18
TNFRSF12A	345	116	1201	0.29
CARD10	249	372	987	0.25
ATF3	91	190	893	0.10
EPHA2	146	116	818	0.18
ISG20	154	143	520	0.30
HMOX1	72	177	417	0.17
ATP10B	89	480	362	0.25
PMAIP1	44	8	350	0.13
VTRNA1-2	38	19	299	0.13
SNORA63	45	76	186	0.24
NAMPT	25	37	183	0.13
MMP10	18	29	151	0.12
IL12A	13	106	62	0.21

Supplemental Table 6. Differential expression of mitochondria related genes in *PARK2* KO neuron samples.

SYMBOL	***PARK2***$^{-/-}$	***PARK2*** $^{-/+}$	**WT**	***PARK2***$^{-/-}$ **/ WT Fold increase**
NR4A2	441	117	30	14.61

GNAQ	352	124	26	13.44
LOC100131323	253	90	22	11.35
LOC399942	1513	465	145	10.40
CACYBP	494	197	52	9.49
LOC728188	567	229	66	8.63
ATP5E	8745	4462	1047	8.35
RPL12P6	1988	1096	259	7.66
LOC346950	809	507	111	7.28
UQCRH	13944	9048	2060	6.77
BLOC1S2	200	103	30	6.75
BLOC1S2	200	103	30	6.75
LOC100130562	2758	1548	438	6.29
LOC653156	10483	10347	1952	5.37
LOC100129685	8345	8142	1639	5.09
ATP7A	93	50	19	4.97
COX17	8464	4974	1772	4.78
COX6B1	17121	9309	3851	4.45
NDUFA12	13530	9681	3243	4.17
PSMC4	863	287	207	4.16
STT3B	103	77	25	4.09
NFE2L2	500	292	123	4.07
NDUFB6	5219	3280	1334	3.91
PPID	90	70	26	3.49
SNCA	776	249	225	3.45
LOC347544	16556	8657	4840	3.42
LOC730167	631	445	185	3.41
LOC652826	380	165	115	3.30
COX7B	6393	2713	1953	3.27
PPARGC1A	393	193	123	3.20
ELAVL2	404	198	130	3.10
LOC651149	4251	2128	1371	3.10
ATF2	318	268	104	3.06
ATP1B1	2984	2193	1027	2.90
SNORD36A	218	97	76	2.87
UQCRB	1408	945	493	2.86
BCL11B	726	508	256	2.84
ATP5EP2	40591	30696	14508	2.80
HRK	158	60	58	2.75
ATP5C1	6577	4742	2404	2.74
ATG4C	124	156	46	2.72
SNORD55	106	73	40	2.64
NAMPT	96	41	37	2.63
SLC25A36	369	205	146	2.53

COX5B	19434	12240	7708	2.52

SYMBOL	*PARK2*$^{-/-}$	*PARK2* $^{-/+}$	WT	*PARK2*$^{-/-}$ / WT Fold decrease
DPF1	426	403	1058	0.40
SNORD3A	262	336	659	0.40
ATP9B	45	115	113	0.40
LOC100131017	37	39	94	0.40
NSUN5B	58	46	149	0.39
NDUFV1	1992	1598	5080	0.39
CDC34	704	627	1801	0.39
ATG9A	211	265	542	0.39
UQCRC1	2805	3803	7240	0.39
SH2B2	170	123	438	0.39
LOC100130276	120	215	312	0.38
LOC642661	35	37	92	0.38
LOC100134424	97	75	254	0.38
LOC642255	43	33	118	0.37
GSK3B	737	1189	2026	0.36
LOC100134364	2330	2757	6409	0.36
UBTD1	120	155	337	0.36
SDHA	440	681	1245	0.35
ATP13A2	190	274	546	0.35
ATP13A2	190	274	546	0.35
PI4KAP1	84	67	246	0.34
DKFZp547K054	24	46	74	0.32
IER5L	151	145	467	0.32
COX19	302	444	947	0.32
LOC100132564	197	297	626	0.31
C1QTNF4	138	130	443	0.31
LRRC4B	71	52	227	0.31
WASH3P	32	33	103	0.31
NACC2	39	91	127	0.31
HIP1R	29	9	98	0.30
LOC389049	68	100	236	0.29
ATP13A1	304	498	1071	0.28
LOC729057	37	36	138	0.27
LOC100008588	2328	2449	8759	0.27
WASH5P	57	66	216	0.26
LOC100132394	3932	4986	15396	0.26
RNU4-2	250	312	1004	0.25
MMP15	178	247	736	0.24
NOVA2	63	54	264	0.24

LOC644284	33	54	142	0.23
UCP2	69	153	296	0.23
ATG4D	78	122	337	0.23
POLRMT	289	520	1362	0.21
MMP23B	18	18	84	0.21
ISLR2	371	133	1812	0.20
ITGA5	32	136	158	0.20
ATPBD3	17	60	97	0.17
RNU4ATAC	165	221	1018	0.16
LOC441193	13	53	81	0.16

Experimental procedures

Generation of iPSC lines from PD patients and controls

Fibroblasts were grown in Minimum Essential Medium Alpha, supplemented with 10-15% (line-specific) fetal bovine serum (FBS), and 1% antibiotic/antimycotic (all from Life Tech., NJ) under 3% O_2, 5% CO_2, 37°C in humidified chamber, and passaged every 5-6 days using TrypLE™ (Life Tech., NJ).

Reprogramming using Sendai virus (SeV, CytoTune™ SeV kit, Life Tech., NJ) was carried out following manufacturer's recommendations and previously described (Sivapatham and Zeng, 2014).

For spontaneous in vitro differentiation, iPSC were detached using collagenase. Cells were cultured in a suspension in ultra-low-attachment plates containing the EB differentiation medium (DMEM/F12 supplemented with StemPro supplement, BSA and FGF2). After 8 days in suspension culture, the EB were transferred to a gelatin-coated plate and cultured in the same medium for another 14 days prior to immunostaining.

Generation of *PARK2* KO isogenic lines by ZFN

ZFN expression plasmids targeting exon1 of *PARK2* gene were purchased from Sigma. Each ZFN polypeptide consists of two functional domains: the DNA binding domain (the recognition sequences of each ZFN are underlined) and the cleavage domain (FokI nuclease). After nucleofection of the *PARK2* ZFN pair in NCRM1, clones expanded from single cells in a 96-well plate were analyzed by DNA sequencing at the junction followed by sequence confirmation and verification of heterozygotes or homozygotes. Several clones with frame shift mutations close to exon 1 were expanded. A heterozygote of *PARK2* (*PARK2*$^{+/-}$) and a homozygote of *PARK2* (*PARK2*$^{-/-}$) was chosen for further analysis, and the mutations details along with the wild type (WT) sequences were shown in Fig. 5B.

MTT Assay

Cell viability was measured using an MTT assay, as previously described. (Peng et al., 2013). Briefly, cells grown in 48-well plates were maintained as required. Five mg/mL MTT tetrazolium salt was

added to each well, and incubated for 4 h at 37 °C. Crystals resulting from mitochondrial enzymatic activity on MTT substrate were solubilized with DMSO in 37 °C for 5 min. Absorbance was measured at 590 nm using a microplate reader (Molecular Devices, Sunnyvale, CA). Cell survival was measured in absorbance difference between treated and untreated cells.

Antibodies

The following primary antibodies were used: NESTIN (611658, BD Transduction laboratories, 1:500), TUJ-1 (clone SDL.3D10, T8660, Sigma, 1:1000), GFAP (Z0334, DakoCytomation, 1:2000), TH (P40101, Pel-Freeze, 1:500), TH (Mouse, 22941, ImmunoStar, 1:500), NANOG (14-5768-82 , eBioscience , 1:100), SOX2 (Ab1125, Abcam, 1:1000), FOXA2 (Ab40874, Abcam, 1:1000). SMA (A2547, Sigma, 1:500), AFP (A8452, Sigma, 1:500), TRA 1-60 (14-8863-82, eBioscience, 1:60), OCT4 (ab19857, Abcam, 1:1250), SOX2 (MAB4343, Millipore, 1:250), and Alkaline phosphatase staining kit II purchased from Stemgent.

Confocal microscopic stereology of mitochondria volume fraction

Control and patient-derived neuron precursors (at day 14 of differentiation) were cultured in 24 or 96 well cover glass-bottomed microplates. Cells were differentiated for another 14 days in PA6-CM in presence of BDNF and GDNF as described previously. On day 28, cultures were loaded with MitoTracker Red CMXRos (75 nM), calcein-AM (1µM) and Hoechst 33342 (5µg/ml) for 30 min and imaged on a Zeiss LSM 780 laser scanning confocal microscope in differentiation medium at 37°C and 5% CO_2. Using a Plan-Apochromat 63×/1.4 oil lens 1024×1024 pixel single planes were recorded at 44 nm pixel size at 1 Airy unit pinhole at recommended spectral settings. Using the Multi Time Series PLUS module of the ZEN 2011 software (Carl Zeiss, Jena, Germany) and Definite Focus autofocusing, first live cell images were acquired automatically along a 10×10 grid while systematically cycling *z*-focus. After fixation and labeling immunofluorescence of Alexa488-labeled TUJ-1 and Alexa647-labeled TH were recorded using stored coordinates and the Hoechst nuclear staining to register images with the live cell micrographs. Recordings were analyzed in Image Analyst MKII (Image Analyst Software, CA). The volume fraction was

calculated using image binarization and summing the number of pixels in all planes corresponding to mitochondrial and cellular profiles. Their ratio multiplied by the stereological correction factor of 2/3 considering projection of mitochondria into the optical thickness of the imaged plane provided the volume fraction (Gerencser et al., 2012). To gate the detection to dopaminergic neurons, regions of binarized images corresponding to the TH staining were manually outlined and the total numbers of pixels corresponding to mitochondrial and cellular profiles were obtained within these shapes. Importantly, a bias in V_F because of altered mitochondrial membrane potential is unlikely. Firstly, fluorescence of MitoTracker Red CMXRos is only partially potential-sensitive (because of lipid partitioning and self-quenching) and it has been shown to stain mitochondria with deficient respiration (Kukat et al., 2008Minamikawa et al., 1999). Secondly, the image processing pipeline performing binarization of MitoTracker images was designed to be little affected by variations in staining intensity (Gerencser et al., 2012).

Electron microscopy

Cells grown on a Thermanox (Nalgene Nunc International) coverslip were fixed for 30 min in 2% (w/v) paraformaldehyde and 2.5% (w/v) glutaraldehyde in 0.1 M sodium cacodylate. Cells were post-fixed in 1% (w/v) osmium tetroxide and 0.8% (w/v) potassium ferrocyanide in 0.1 M sodium cacodylate for 60 min, then stained with 2% (w/v) uranyl acetate for 30 min. Dehydrated and EMbed-812 infiltrated samples were embedded in EMbed-filled BEEM (Electron Microscopy Sciences,Hatfield, PA, USA) capsules at 60◦C for 72 h. Using an MT-7000 ultramicrotome, 70 nm-thick sections were generated and imaged on a Phillips Technai 12 transmission electron microscope at 80 kV at 68,000x.

Immunocytochemistry

The quantification of immunoreactive cells in culture was performed by analyzing fluorescent images using Adobe Photoshop. Cell counts were expressed as a percentage of total cells in a field. Total

number of cells was represented by the number of Hoechst-labeled nuclei on each image. Four different randomly chosen fields from four independent experiments were counted by three different individuals. Values were obtained by evaluating at least 600-750 TH-positive cells per experiment. Statistical analysis was performed using the Student's t-test with two-tailed distribution and assuming equal variance.

Whole genome expression analysis

The background method was used for normalization. The maximum expression value of gene for probe set was used as the expression value of the gene. For the processed data, the dendogram was represented by global array clustering of genes across all the experimental samples using the complete linkage method and measuring the Euclidian distance. Expression of sample correlations was a measure of Pearson's coefficient, implemented in R System.

qPCR analysis

Quantitative PCR reactions were carried out on the CFX96™ Touch Bio-Rad instrument (Bio-Rad, CA) using iTaq™ Universal SYBR® Green supermix (Bio-Rad, CA) according to the manufacturers' instructions. PCR reactions were conducted in duplicate or triplicate for each sample. Genomic DNA contamination and RNA quality were assayed using PrimePCR™ control assays (Bio-Rad, CA). For microarray validation experiments samples included Y9 (control), A6, A23 (*SNCA* triplication), I3, P1, B119, S110 (*PARK2* mutants), K20, K25 (*LRRK2* mutants), and T101 (*GBA* mutant) at dopaminergic stage (28 days of differentiation). Human *TBP, GAPDH,* and *ACTB* were amplified as internal standards. Reported values were calculated using ΔΔCt method and normalized against endogenous *ACTB* (pluripotency and SeV genes) or *TBP* and *GAPDH* (NSC and DA gene expression). Primer sequences were previously described(Sivapatham and Zeng, 2014).

Western blot analysis

Following SDS-polyacrylamide gel electrophoretic separation, proteins were transferred to 0.22 mm PVDF membrane (Bio-Rad, CA). Blocking was done in TBS with 5% milk and 0.1% Tween (all from Bio-

Rad, CA) for 1 hour at room temperature. Membranes were incubated with the following antibodies at 4°C overnight: α-synuclein (BD Biosciences, 1:750), TH (Pel-Freeze, 1:1500), TH (Sigma, 1:1500), Horseradish peroxidase conjugated secondary antibodies (Life Tech., NJ) were diluted in blocking buffer and incubated for 1 hour at room temperature. Detection of bound antibodies was performed using ECL Advance Western Blotting Detection kit (Amersham Biosciences, NJ) and chemiluminescent signal was recorded on Hyperfilm (Amersham Biosciences, NJ).

References

Gerencser, A.A., Chinopoulos, C., Birket, M.J., Jastroch, M., Vitelli, C., Nicholls, D.G., and Brand, M.D. (2012). Quantitative measurement of mitochondrial membrane potential in cultured cells: calcium-induced de- and hyperpolarization of neuronal mitochondria. The Journal of physiology *590*, 2845-2871.

Peng, J., Liu, Q., Rao, M.S., and Zeng, X. (2013). Using human pluripotent stem cell-derived dopaminergic neurons to evaluate candidate Parkinson's disease therapeutic agents in MPP+ and rotenone models. Journal of biomolecular screening *18*, 522-533.

Sivapatham, R., and Zeng, X. (2014). Generation and Characterization of Patient-Specific Induced Pluripotent Stem Cell for Disease Modeling. Methods in molecular biology.
