# Supplementary material for: Mitochondrial Alterations by PARKIN in Dopaminergic Neurons Using PARK2 Patient-Specific and PARK2 Knockout Isogenic iPSC Lines
Source: Stem Cell Reports. 2015 Apr 2;4(5):847–59. doi: 10.1016/j.stemcr.2015.02.019 (PMC4437475; doi:10.1016/j.stemcr.2015.02.019)
Supplement: Document S2. Article plus Supplemental Information [file mmc2.pdf]

Stem Cell Reports
Article
OPEN ACCESS

Mitochondrial Alterations by PARKIN in Dopaminergic Neurons Using PARK2 Patient-Specific and *PARK2* Knockout Isogenic iPSC Lines

Atossa Shaltouki,[1,4] Renuka Sivapatham,[1,4] Ying Pei,[1,4] Akos A. Gerencser,[1] Olga Momčilović,[1] Mahendra S. Rao,[2] and Xianmin Zeng[1,3,*]
[1]Buck Institute, Novato, CA 94945, USA
[2]NxCell Science, Novato, CA 94947, USA
[3]XCell Science, Novato, CA 94947, USA
[4]Co-first author
*Correspondence: xzeng@buckinstitute.org
http://dx.doi.org/10.1016/j.stemcr.2015.02.019
This is an open access article under the CC BY license (http://creativecommons.org/licenses/by/4.0/).

SUMMARY

In this study, we used patient-specific and isogenic PARK2-induced pluripotent stem cells (iPSCs) to show that mutations in PARK2 alter neuronal proliferation. The percentage of TH^+ neurons was decreased in Parkinson's disease (PD) patient-derived neurons carrying various mutations in *PARK2* compared with an age-matched control subject. This reduction was accompanied by alterations in mitochondrial:cell volume fraction (mitochondrial volume fraction). The same phenotype was confirmed in isogenic *PARK2* null lines. The mitochondrial phenotype was also seen in non-midbrain neurons differentiated from the *PARK2* null line, as was the functional phenotype of reduced proliferation in culture. Whole genome expression profiling at various stages of differentiation confirmed the mitochondrial phenotype and identified pathways altered by PARK2 dysfunction that include PD-related genes. Our results are consistent with current model of PARK2 function where damaged mitochondria are targeted for degradation via a PARK2/PINK1-mediated mechanism.

INTRODUCTION

PARKIN (*PARK2*), an E3 ubiquitin ligase, is the most frequently mutated gene that has casually been linked to autosomal recessive early onset familial Parkinson's disease (PD) (Abbas et al., 1999; Kitada et al., 1998). Abnormalities of *PARK2* have also been described in sporadic PD (Dawson, 2006). The exact mechanism by which *PARK2* causes PD-like syndromes and why dopaminergic neurons are primarily affected by a ubiquitously expressed mutation remain unknown (Sulzer, 2007; Tanaka et al., 2004). Several studies, however, suggest that *PARK2* interacts with *PINK1*, another gene mutated in autosomal recessive familial form of PD (Geisler et al., 2010) to regulate mitochondrial biology, and alters mitochondrial dynamics (Chen and Chan, 2009; Clark et al., 2006; Lee et al., 2004).

A link between PARK2 and mitochondrial biology was first established in *Drosophila*, which displayed impairment in mitochondrial function and neuronal loss in an age-dependent manner when rendered deficient for PARK2 (Greene et al., 2003). Likewise, similar mitochondrial defects exhibited in *Park2* knockout (KO) mouse models, although only mice with conditional KO of *Park2* recapitulate parkinsonian phenotype and striatonigral degeneration (Dawson et al., 2010; Goldberg et al., 2003). Analysis of single and double mutants in mice and flies also suggests that *Pink1* is upstream of *Park2* and that overexpression of PARK2 alone or directing PARK2 to mitochondria is sufficient to introduce mitochondrial fragmentation (Akundi et al., 2013; Clark et al., 2006; Kim et al., 2008; Shiba-Fukushima et al., 2012). Thus, both gain or loss of function can affect mitochondrial dynamics. More recently, post-mortem brain tissues of PD patients also confirmed the involvement of altered mitochondrial pathologies in disease process (Henchcliffe and Beal, 2008; Schapira et al., 1989; Vila et al., 2008).

The emerging hypothesis is that in normal cells PARK2 is cytoplasmic and PINK1 levels are low. However, when mitochondrial potential is lost, PINK1 accumulates on depolarized membranes and recruits PARK2 to mitochondria and are then targeted for degradation via mitophagy. Loss or damaged mitochondria stimulate mitochondrial fission and/or inhibit fusion by negatively regulating MFN and OPA1 function and/or positively regulating DRP1 (van der Bliek et al., 2013).

Despite these advances, differences between species in displaying neurodegenerative phenotypes have made it difficult to extrapolate the results obtained from animal models to human. The discovery of induced pluripotent stem cells (iPSCs) has for the first time enabled us to reproduce dopaminergic neurons from individuals who suffer from familial or sporadic PD. Indeed, a recent iPSC-based study showed that PARK2 controlled dopamine utilization in iPSC-derived dopaminergic neurons (Jiang et al., 2012). Likewise, advances in gene targeting (Cathomen and Joung, 2008; Urnov et al., 2010; Zeng et al., 2014) allow us to develop the corresponding models in an isogenic background.

To enable us to study the role of PARK2 in human PD, we made integration-free iPSC lines from four PD patients carrying different *PARK2* mutations (NINDS collection; Table S1). We showed a deficiency in dopaminergic differentiation and a reduction in mitochondrial volume fraction in all four PARK2 lines compared with an age-matched control subject. To confirm the results from the patient-specific disease model and to overcome the genetic variation among patient lines that could mask the PARK2 phenotype, we generated *PARK2* isogenic controls using a KO strategy in a well-characterized integration-free iPSC line. We found similar phenotypes in the *PARK2* KO isogenic line as seen from the familial PARK2 lines. We showed that loss-of-function mutations in PARK2 impaired dopaminergic development by reducing the percentage of Tyrosine hydroxylase-positive (TH^+) neurons and accumulation of α-synuclein (SNCA) in dopaminergic neurons. These results were supported by whole genome expression profiling in which alterations in expression of mitochondria and cell death-related genes were observed in the dopaminergic neuron stage but not in earlier stages of differentiation. In addition, we showed that similar changes were detected in a pure population of forebrain neurons derived from the isogenic model. Our results suggest that PARK2 is involved in mitochondrial regulation in neurons.

RESULTS

Generation of Integration-free iPSC Lines from Four Patients with Various *PARK2* Mutations

To investigate why mutations in PARK2 cause selective degeneration of dopaminergic neurons in humans, we first used a patient-specific-based-iPSC approach. Fibroblasts from four patients (I, P, B, S) with various mutations in *PARK2* and an aged-matched control subject (Y) were used to generate iPSC lines. Table S1 lists the clinical and demographic data associated with each cell line. Whole genome expression analysis was performed on the fibroblasts to obtain baseline data on the samples. No significant difference in overall gene expression in major PD genes was observed (Table S2).

Integration-free iPSC lines were generated by Sendai technology (Cathomen and Joung, 2008; Pavletich and Pabo, 1991; Wang et al., 2013; Yang et al., 2008). Multiple clones from each subject were isolated and expanded and validated for pluripotency ability to differentiate to three germ layers absence of vector integration in iPSC lines and identity by STR analysis and normal karyotype over long-term culture (>20 passages) in vitro (Figures 1A–1P). One clone of each line referred as Y09 (control), I3, P1, S110, and B119 (PARK2 patients) was chosen for this study.

At this stage, no difference was observed between the control line and the four PARK2 patient lines by growth rate, morphology, and whole genome analysis. Thus, neither the fibroblasts nor the iPSC derived from them displayed an obvious phenotype. PARK2 levels were low or undetectable in normal fibroblasts and normal iPSC lines, suggesting a possible explanation.

Neural and Neuronal Differentiation of PARK2 and Control iPSC Lines

We next determined whether the PARK2 iPSC lines could differentiate to neuronal lineage. We observed no different NSC formation between the patient and control lines, and all lines differentiated into dopaminergic neurons (Figures 2A–2D). The experiment was repeated several times (n = 4), and no difference was observed in any biological replicates. Whole genome profiling of each line at various stages of differentiation could not distinguish the patient samples from the control (Figure S1). Instead, samples were clustered by cell types as expected: hierarchial clustering of NSC and dopaminergic neurons revealed a similar gene expression pattern for all of the iPSC generated. The overall correlation coefficiency between each population showed highest similarities (higher R^2 value) among each population (e.g., iPSC versus iPSC, $R^2 > 0.98$) (Table S3). Examining the dataset at a higher resolution also showed appropriate temporal expression of stage specific markers such as LMX1A, FOXA2, and AADC, which were expressed in all the generated lines at the dopaminergic stage (data not shown), similar to what we had previously observed in our experiments (Liu et al., 2013; Momčilović et al., 2014).

Impaired Dopaminergic Differentiation of PARK2 Lines

Although the ability of the normal and PARK2 patient NSC differentiated in a qualitatively similar fashion, we did note that the efficacy of dopaminergic differentiation appeared to be reduced in the patient lines. To obtain a more quantitative measure, we counted TH^+ cells in each line by immunocytochemistry and observed a significant decrease in TH^+ neurons from the patient lines: about 22% of total cells expressed TH in the control line, whereas only 15%, 7%, 7% and 7% of total cells were TH^+ dopaminergic neurons from B119, I3, P1, and S110 PARK2 lines, respectively (Figure 3A). We repeated the experiment several times (n = 4), and although the actual percentages varied in all cases, the number of TH^+ cells in the PARK2 lines was always less than in the control. Given the temporally appropriate onset of dopaminergic precursor markers in the array analysis of patient and control lines, we interpret this result to suggest that the changes we observed were likely the result of death of differentiating or fully differentiated cells in the

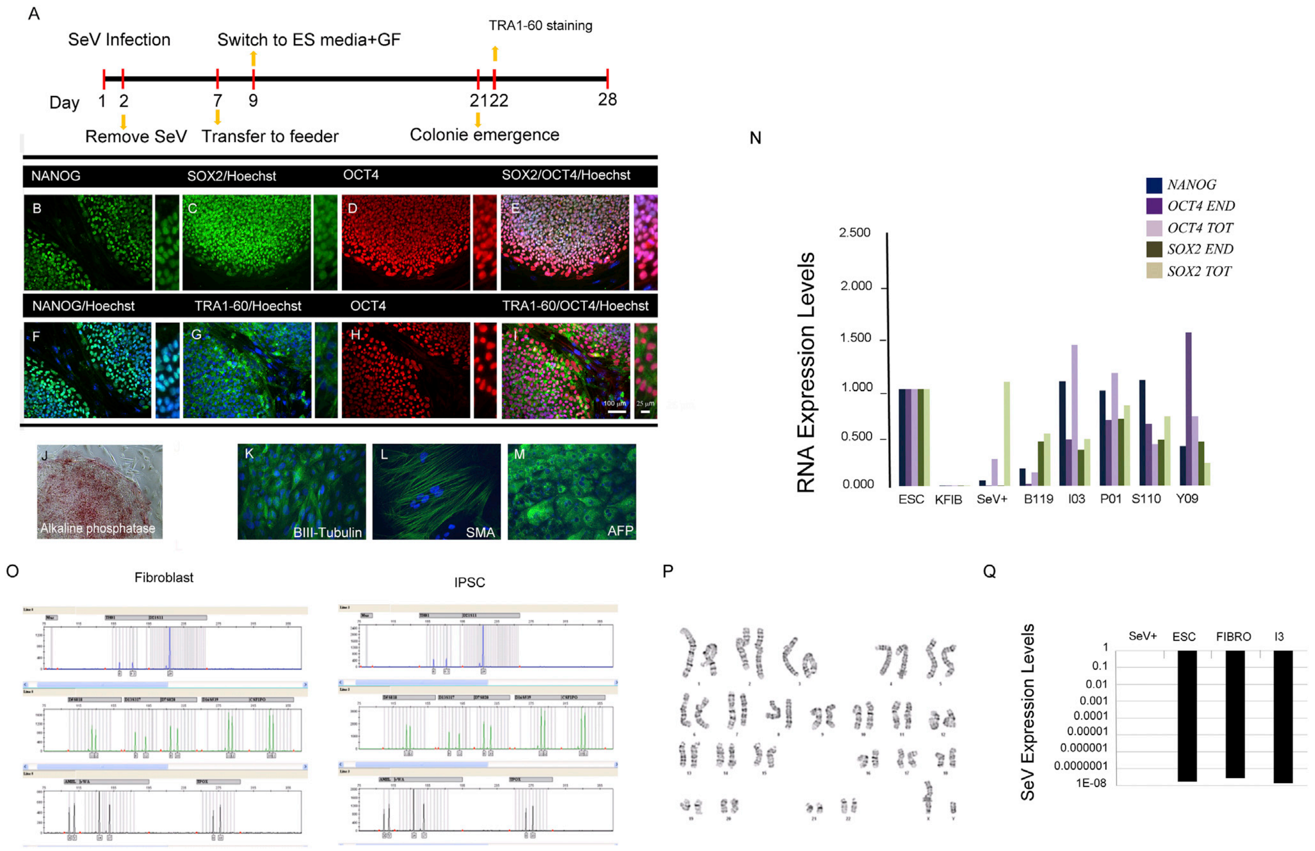

Figure 1. Generation and Characterization of Parkinson's Disease-Derived iPSC Using Sendai Virus Vector

(A) The workflow outlined here demonstrated the generation of human iPSC with Sendai virus vector encoding OCT4, KLF4, SOX2, and cMYC. Approximately 5 × 10^5 human fibroblasts were plated onto a 35-mm dish 1 day before transduction. Day 1 denotes a day of transduction. About 6 to 7 days after transduction, cells were collected and transferred onto inactivated mouse feeders at the density of 5 × 10^5 cells per 10-cm dish.

(B–J) Approximately 3-weeks posttransduction, TRA1-60-positive colonies were picked manually and transferred to a fresh feeder-coated well. This shows representative colonies of PARK2 patient iPSCs stained for pluripotent markers NANOG, SOX2, OCT4, and TRA-1-60 (B–I), as well as colonies positive for alkaline phosphatase activity (J).

(K–M) Immunofluorescence analysis of PARK2-PD-iPSC differentiated in vitro show the potential to generate cell derivatives of all three primary germ cell layers, including ectoderm (stained for TUJ-1, green), mesoderm (stained for smooth muscle actin [SMA], green), and endoderm (stained for a-fetoprotein, green).

(N) qPCR analyses of the endogenous (genomic) and exogenous (Sendai virus vector) expression levels of the indicated genes in PARK2 patient iPSCs.

(O) Short tandem repeat (STR) analysis of genomic DNA from PARK2-PD-iPSC matched the identity of iPSCs to their parent fibroblasts.

(P) Normal karyotype of PARK2 patient iPSC at passage 20.

(Q) The absence of persistent Sendai virus in I3 PARK2 patient fibroblasts and iPSC was confirmed as determined by RT-PCR analysis.

Results are representative of three biological replicates (individual clones in case of iPSC) from three independent experiments. Scale bars are 100 μM.

patient-derived cultures rather than fewer dopaminergic neurons being born in the patient lines.

Since death of dopaminergic neurons in PD is often accompanied by changes in SNCA protein, we examined SNCA expression in dopaminergic neurons of the patient and control lines. As seen in Figure 3B, SNCA or its aggregates were not detected in dopaminergic neurons derived from the control line. However, SNCA and two forms of SNCA aggregates were all elevated in all patient lines by western blot (Figure 3B). The increased expression of SNCA in PARK2 patient lines was also observed by immunostaining of SNCA: approximately 4.6% of total cells were $SNCA^+$ in the control Y09 line, whereas approximately 12.1%, 7.4%, 8.5% and 9.0% of total cells were

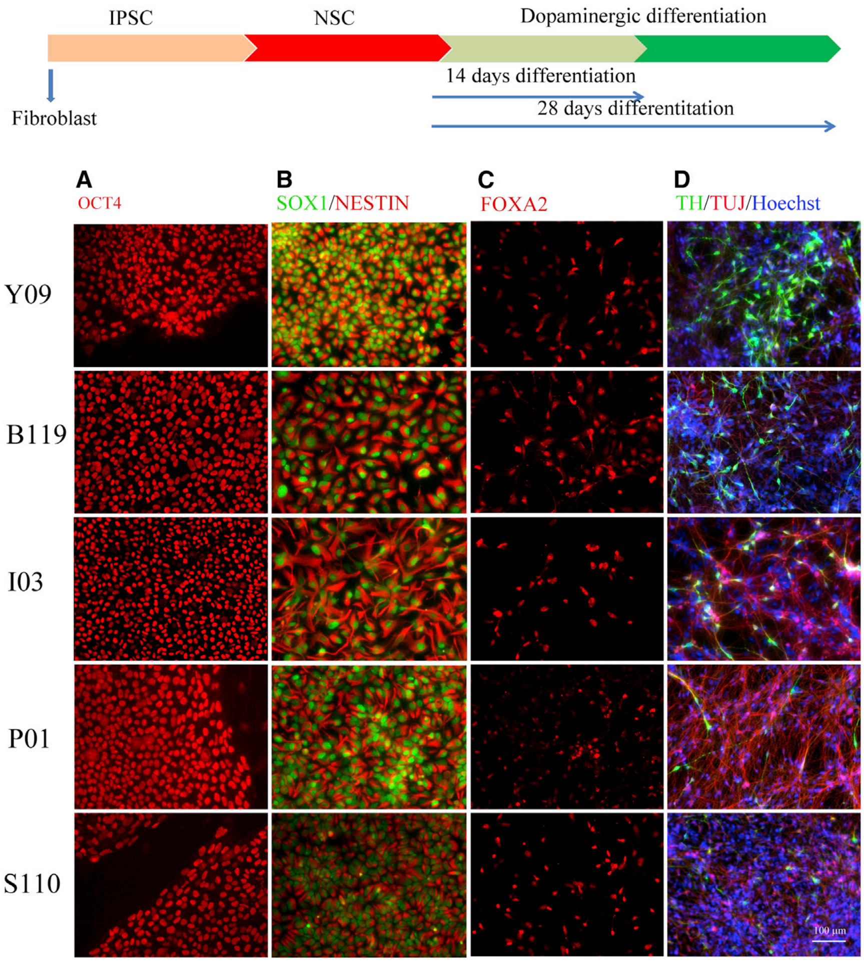

Figure 2. Dopaminergic Differentiation of Integration-free iPSC Lines Generated from Four PARK2 Patients and One Control

(A) A scheme of differentiation of dopaminergic neurons from patient-derived iPSC.

(B) Patient-derived iPSC and iPSC- from the age-matched control subject were directed to neuroepithelial cells using the previously described protocol (Swistowski et al., 2009).

(C) These neuroepithelial cells were exposed to PA6-CM to produce FOXA2 expressing dopaminergic progenitors in the subsequent 2 weeks.

(D) Finally, the progenitors were further differentiated to post-mitotic dopaminergic neurons by the addition of neurotrophic factors to PA6-CM, as described previously. Control and patient-specific iPSC-derived neurons were analyzed by immunofluorescence for expression of TH (green) and TUJ-1 (red) at the end of the 28-day differentiation protocol. Shown are representative images of three independent differentiation experiments from Control-iPSC, PARK2 patient iPSC lines carrying different *PARK2* mutations.

Scale bars = 100 μM.

SNCA positive in the patient lines I3, P1, B119, and S110, respectively (Figures 3C and 3D). Double immunostaining of TH and SNCA revealed that enhanced SNCA expression was not limited to TH^+ cells, as only a small percentage of the SNCA-positive cells were TH^+ neurons for all lines (1.3% for the control Y09 line and 2.4%, 0.8%, 3.2%, and 0.6% for the patient lines I3, P1, B119, and S110, respectively) (Figure 3D). These results suggested that mutations in *PARK2* gene may contribute to stress in neuronal cultures that leads to the accumulation of SNCA in dopaminergic neurons. The accumulation of aggregates may contribute to the reduction in the number of TH^+ neurons observed in the differentiating cultures form *PARK2* mutated patient lines.

Reduced Mitochondrial Volume Fraction in PARK2 Patient Lines

Since we observed a phenotype in all four patient lines, which in PARK2 may be related to mitochondrial dysfunction, we examined mitochondrial biology in more detail. We first determined mtDNA copy number in dopaminergic cultures from the PARK2 and control lines. No significant changes were seen in the amount of mitochondria DNA measured by qPCR against the nuclear DNA between the patient lines and the control or among the PARK2 lines (Figure 4A). Nor did we find an alteration in mitochondrial volume fraction, as determined by the ratio of mitochondria to cell volume in total cells (Figure 4B).

However, since our cultures consist of a mixed population of cells, we reexamined the cultures focusing solely on TH^+ cells. This was done post hoc by double labeling cells for TH and TUJ-1 after live imaging of at least 600 cells from each control and patient line (Figures 4C–4H). MitoTracker staining (live) in TH^+ (post hoc) neurons from the control line and a representative PARK2 line are shown in Figures 4E and 4H. As seen in Figure 4I, the fraction of mitochondria (MitoTracker; green) to cell volume (Calcein-AM; gray) calculated from confocal images constrained to TH^+ neurons in all four PARK2 lines was significantly lower than in the control line. These results indicate that mutations in PARK2 contribute to changes in mitochondrial content in dopaminergic neurons in these cultures, but at

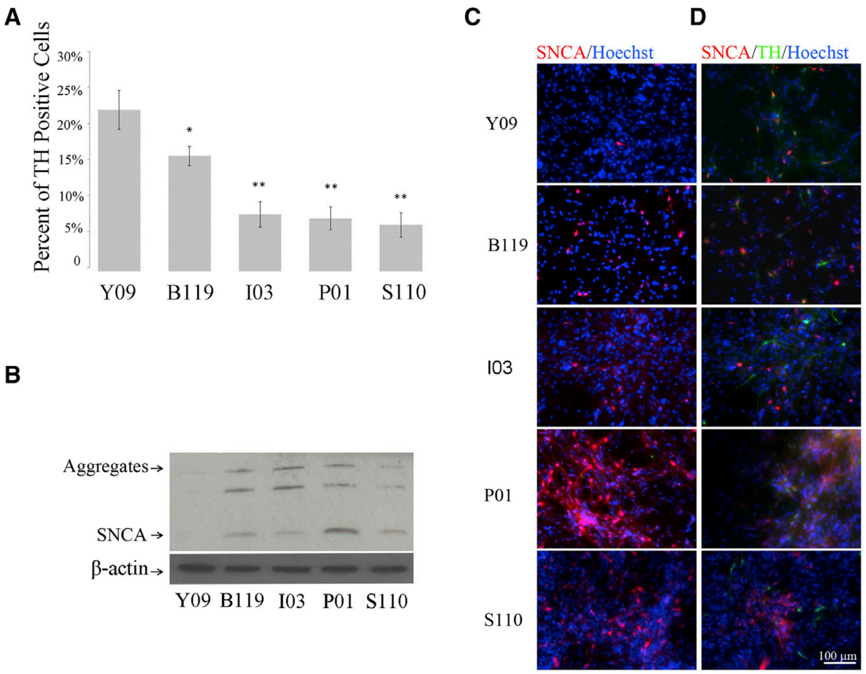

Figure 3. Decreased TH-Positive Neurons and Increased SNCA Expression in iPSC Lines Carrying *PARK2* Mutations

(A) To determine differentiation efficiency, the number TH-positive cells is represented as the percentage of total number of cells (stained with Hoechst). Error bars represent mean SEM of triplicates from four independent experiments. Significant differences were found in the ability of iPSCs from different patients to generate dopaminergic neurons after 28 days of differentiation.

(B) Western blot analysis of extracts from patient-derived neurons probed with SNCA antibodies. The arrow marks the 19-kDa SNCA present in patient lines and absent in control healthy subject. The band just above (present in only patient lines) may represent an alternatively spliced form of SNCA or aggregate form of SNCA.

(C) Immunofluorescence for expression of SNCA in dopaminergic neurons from patient lines. Increased SNCA expression is observed in PARK2 patient lines.

(D) Double stain of SNCA and TH expression in dopaminergic neurons from patient lines. SNCA is expressed in both TH-positive and non-TH-expressing neurons. Shown are representative of four independent experiments.

Scale bars represent 100 µM.

this stage, no changes are seen in the other cell types present in the culture including astrocytes, NSC, and other midbrain neurons.

Decreased Dopaminergic Differentiation and Mitochondrial Volume Ratio in Isogenic *PARK2* KO iPSC Lines

Although the results were compelling and consistent, the size of the cohort examined (four) is small, and the data do not allow us to conclude that PARK2 is sufficient to cause the observed phenotype. To address this issue, we obtained a set of isogenic iPSC lines mimicking the loss of function of *PARK2* gene created by Zinc Finger Nuclease (ZFN) in a well-characterized integration-free iPSC line XCL1 (XCell Science). Figures 5A and 5B showed a schematic representation of ZFN binding to *PARK2* and the frame-shift mutations introduced in the heterozygotes ($PARK2^{+/-}$) and homozygote ($PARK2^{-/-}$) used in this study. Both lines were validated for pluripotency and normal karyotypes (Figures 5C–5E). As expected, gene expression profiling did not show a phenotype at the iPSC stage. These data confirmed that the targeting process did not alter the line, and consistent with the patient line data, loss of PARK2 did not affect iPSC behavior. We next generated NSC from these isogenic lines (Figures 6A–6C). Consistent with earlier results, no difference in early neural differentiation was observed between the lines. Both NSC lines could differentiate into TH^{+} dopaminergic neurons (Figures 6D–6F). Quantification of the percentage of TH^{+} dopaminergic neurons revealed a significantly lower percentage in the $PARK2^{-/-}$ line than in the WT control line (Figure 6G). We then examined the mtDNA copy number and mitochondrial volume fraction in TH^{+} dopaminergic neurons. No difference in DNA copy number or mitochondrial volume fraction in total cells in the mixed culture was found between the $PARK2^{-/-}$ and its isogenic control (data not shown). However, similar to what we discovered in the patient-derived lines, mitochondrial volume fraction in TH^{+} neurons was significantly reduced in the $PARK2^{-/-}$ line, but not in the $PARK2^{-/+}$ line (Figure 6H).

This loss of *PARK2* was both necessary and sufficient to mimic the phenotype observed in the patient lines. To assess whether there were changes in mitochondrial biology that were large enough to be detected by microarray analysis, we prepared dopaminergic neurons from the WT and $PARK2^{-/-}$ KO line and assessed the expression of ~600 mitochondrial genes, mitophagy-related genes, genes known to be involved in cell death, and mitochondrial fission and fusion (Table S4). We reasoned that sensitivity should be higher in isogenic line comparison, and >2-fold changes may be enriched in genes that are biologically relevant genes. Consistent with our observation of a change in mitochondrial volume fraction in dopaminergic neurons, expression of several mitophagy-related genes

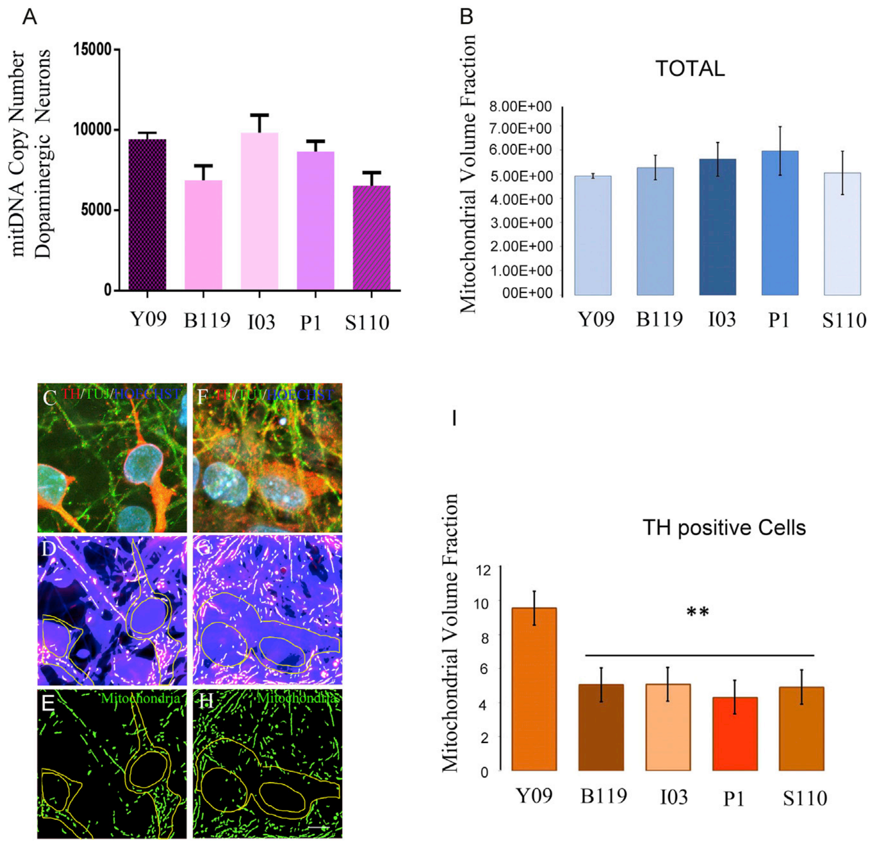

Figure 4. Changes in Mitochondrial Content in PARK2 Patient-Derived Dopaminergic Neurons

(A) A variation of mtDNA copy number in patient-derived dopaminergic neuron mixed culture. Bars represent average with SEM as error bars. Results are representative of three independent experiments.

(B–H) Mitochondrial volume fraction determination using confocal microscopy. The ratio of mitochondria (MitoTracker Red; green) to total cellular volume (Calcein-AM; gray) was calculated from confocal images for TH-positive cells. The upper two panels show the TH, TUJ-1-positive cells in control subject (C) and patient-derived neurons (F). The middle two panels show the binary processed images (D and G), which were used as input for the calculation. The lower two panels show mitotracker staining in TH-positive cells (E and H). Mitochondrial volume fraction quantification from confocal imaging. Bars represent means SEM of triplicates from three independent experiments (I). The statistical significance was calculated from a one-way ANOVA; $^{**}p < 0.001$. The scale bar in (C)–(H) represents 75 μM.

was altered in the $PARK2^{-/-}$-derived dopaminergic population. Twenty-five genes were seen to be 2-fold or higher expressed in the $PARK2^{-/-}$ line, whereas 20 genes were expressed 2-fold or lower in the $PARK2^{-/-}$ line (Table S5).

Overall, these data are consistent with our observation that *PARK2* null is a sensitive model of familial PARK2 patient-specific lines and can be used to assess the role of PARK2 in disease.

Mitochondrial Ultrastructural Abnormalities in $PARK2^{-/-}$ KO Line

To provide a more morphological assessment of the phenotype, we examined change of mitochondria in $PARK2^{-/-}$ KO cells by electron microscopy. Cells from the isogenic control and the $PARK2^{-/-}$ line were grown in parallel and differentiated into dopaminergic neurons. The mitochondrial morphology was examined in neurites and in cell soma using thin-cut electron microscopy sections. As a control, the normal cells were treated with rotenone at 100 μM for 24 hr, a well-characterized mitochondrial toxin, which induces characteristic changes in mitochondria that include changes in mitochondrial volume (Chauvin et al., 2001; Panov et al., 2005; Sherer et al., 2003a).

As seen in Figures 7A and 7B, rotenone caused overt swelling and loss of matrix density in the WT control cultures. These ultrastructural alterations are similar to what has been previously described (Chauvin et al., 2001; Lin et al., 2012). As with WT cells treated with rotenone treatment, $PARK2^{-/-}$ cells also showed similar changes in mitochondria present in the cell soma (Figure 7C). Mitochondria in $PARK2^{-/-}$ neurons showed signs of swelling (decreased density of the mitochondrial matrix; Figure 7C, open arrows) and irregular, dilated cristae (arrows) as compared with the isogenic control. These differences were not observed in mitochondria present in neurites (Figures 7D–7F), suggesting a more limited mitochondrial damage. These data confirm and extend our observation that *PARK2* null neurons undergo stress in culture, which leads to mitochondrial damage and a slow progressive cell death.

Altered Gene Expression of $PARK2^{-/-}$ Neurons and Its Response to Stress

Since PARK2 is ubiquitously expressed, we reasoned that a subset of mitochondrial changes observed in dopaminergic cultures by microarray would likely be seen in other neuronal cell types. We therefore prepared a pure population of neurons from these isogenic lines using a protocol

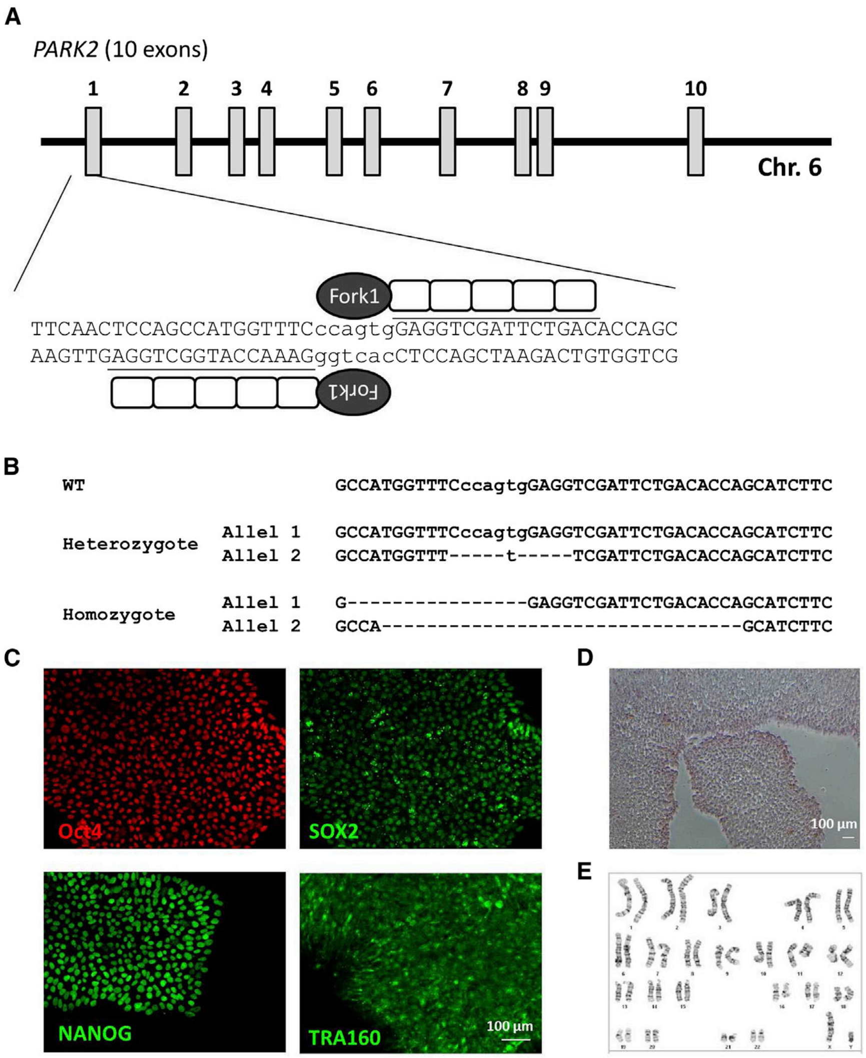

Figure 5. Generation of Isogenic *PARK2*$^{-/-}$ iPSC Lines

(A) Schematic representation of ZFNs binding to human *PARK2*. Each ZFN polypeptide consists of two functional domains: DNA binding domain (the recognition sequences of each ZFN are underlined) and the cleavage domain (FokI nuclease).

(B) Mutations details of *PARK2* heterozygote (*PARK2*$^{+/-}$) and homozygote (*PARK2*$^{-/-}$) generated by ZFNs technology. WT sequences were shown in the top lane as reference.

(C) *PARK2*$^{-/-}$ iPSC maintained expression of pluripotency markers such as OCT4, SOX2, NANOG, and TRA1-60.

(D) *PARK2*$^{-/-}$ iPSC showed normal alkaline phosphatase staining.

(E) *PARK2*$^{-/-}$ iPSC showed a normal karyotype (46, XY).

by which greater than 95% of the total cells at day 14 expressed TUJ-1 (Figure S2A) (Liu et al., 2013). We first examined whether the *PARK2*$^{-/-}$ neurons were more stressed compared with the WT neurons. Morphologically, neuronal differentiation from both WT and *PARK2*$^{-/-}$ lines is similar, and no alteration in cell lineage was detected (Figure S2B). Short-term survival in culture as assessed by 3-(4,5-dimethylthiazol-2-yl)-2,5-diphenyltetrazolium bromide (MTT) assay at days 8, 10, and 14 showed a gradual loss of cells in *PARK2*$^{-/-}$ line as compared with the WT, as there was significant fewer neurons from the *PARK2*$^{-/-}$ line by day 14 when an equal number of WT and *PARK2*$^{-/-}$ cells were seeded at day 6 (Figure S2B), suggesting that loss of PARK2 function can cause cell death/stress.

We then performed a whole genome expression analysis on day 14 neurons of the isogenic lines and focused our analysis on alteration in expression of mitochondrial related genes. Ninety-five genes had altered expression by 2-fold; of them, 45 were upregulated, and 50 were downregulated in the mutant (Table S6). There was an increase in levels of a subset of mitochondrial genes and an upregulation of cell death genes. Of importance to note was the upregulation of SNCA in these cultures along with the upregulation of autophagy-related genes (Table S6). Of the *BCL2* family in the cell death gene dataset, *HARAKIRI* in particular appeared upregulated. Overall these results confirm the effect of *PARK2* mutations on mitochondria and are consistent with a mitochondrial abnormality inducing stress in cells, which leads to a BAD/BAX-mediated cell death.

DISCUSSION

Mutations in the *PARK2* gene are associated with PD, although the exact mechanism by which PARK2

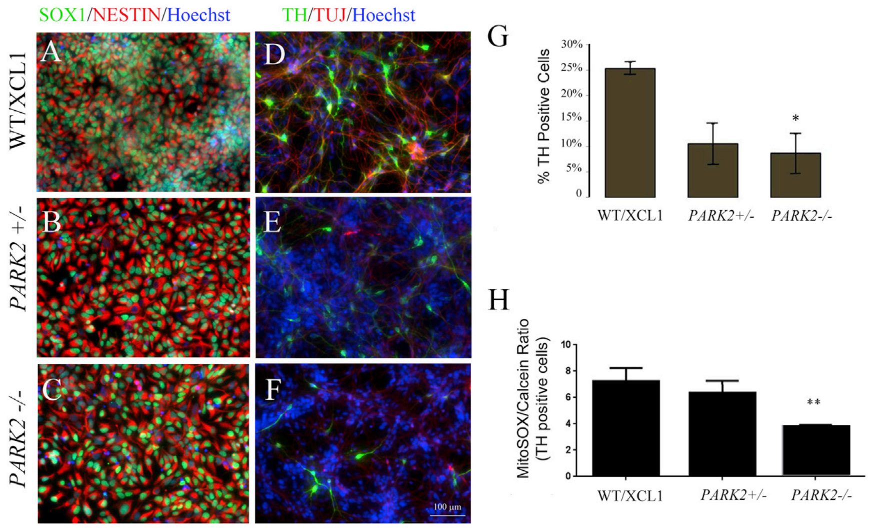

Figure 6. Decreased Dopaminergic Differentiation and Mitochondrial Abundance in Isogenic *PARK2*$^{-/-}$ iPSC Lines

(A–C) No detectable difference was observed in NSC uniformly expressing Nestin and Sox1 derived from WT, *PARK2*$^{+/-}$ and *PARK2*$^{-/-}$ iPSC lines.

(D–F) Loss of dopaminergic neurons in *PARK2*$^{+/-}$ and *PARK2*$^{-/-}$ lines compared with the WT. Representative immunohistochemistry images of dopaminergic differentiated culture of *PARK2*$^{+/-}$ and *PARK2*$^{-/-}$ iPSC and their parental isogenic control line were stained for dopaminergic marker TH and TUJ-1.

(G) Percent of TH-positive dopaminergic neurons *PARK2*$^{+/-}$ and *PARK2*$^{-/-}$ cells compared with their parental isogenic control WT line at day 28.

(H) A significantly reduced mitochondrial volume fraction in TH-positive dopaminergic neurons was observed in the *PARK2*$^{-/-}$ line. Bars represent means and SEM of four independent experiments. $^{**}p < 0.001$ (Student's t test).
Scale bars represent 100 μM.

contributes to the selective neuronal degeneration in PD is unknown. Different lines of evidence indicate that alterations in many aspects of mitochondrial biology such as complex I activity, fission and fusion, mitophagy, transport of mitochondria in neurons, and alterations mitochondrial membrane potential may contribute to PD (Dauer and Przedborski, 2003; Exner et al., 2012). Consistent with the mitochondrial hypothesis, it has been postulated that the role of PARK2 and PINK1 in mitochondrial quality control underlies the basis of PARK2-related PD. Our results showing an alteration in mitochondrial volume in *PARK2* mutants in a primary human dopaminergic cell model is consistent with this hypothesis. The deficits in mitochondrial volume were accompanied by a reduction in dopaminergic neurons in PARK2 patient lines, and these phenotypes were recapitulated in our isogenic *PARK2*$^{-/-}$ lines. Whole genome expression profiling confirmed the phenotype and identified mitochondrial-associated cell death as a cause for the reduction in cell number.

Mitochondria play an important role in neuronal activity and survival. Neurons rely on oxidative phosphorylation for their energy supply, and the abundance of mitochondria is an important factor in determining survivability of neurons (Yadava and Nicholls, 2007). We have previously reported that differentiated neurons display higher mitochondrial biogenesis when compared with their early progenitors NSC (Birket et al., 2011). Misregulated biogenesis has been implicated to underlie pathological conditions in a number of neurodegenerative diseases. A host of proteins such as VDAC, cytochrome C, POLG, TFAM and PGC-1α, NRF-1 are known to regulate mitochondrial biogenesis, and differential expression of these proteins has been reported in various neurodegenerative disorders. Our array data did not show substantive differences in expression of these mitochondrial biogenesis genes between PARK2 patients and controls, indicating that the phenotype was not caused by mitochondrial biogenesis.

Deletion and overreplication of mtDNA are emerging as important factors underlying the selective loss of dopaminergic neurons during aging and in PD (Anderson et al., 1981; Ekstrand et al., 2007; Johns, 1995; Wei, 1998). Although we did not detect any changes in mtDNA copy numbers between healthy and diseased samples, the mitochondria-to-cell-volume fraction, an important parameter of mitochondrial membrane potential (Birket et al., 2011), was significantly reduced in PARK2 dopaminergic neurons. A decrease in mitochondrial membrane potential in PARK2-deficient cells could make a selective population of cells more vulnerable to stress stimuli. Consistent with this, PARK2 mutant *Drosophila* have been reported to accumulate depolarized mitochondria in dopaminergic neurons (Burman et al., 2012). Indeed, a decline mitochondrial membrane potential has been reported in PD patient derived fibroblasts, with PARK2 deficiency (Mortiboys et al., 2008).

Our results are consistent with previous reports of the action of PARK2 and reduction in TH-positive cells described in mouse *PARK2* KO model (Perier et al., 2013; Reeve et al., 2013; Rothfuss et al., 2009) and consistent with work done in the fly model. The mitochondrial phenotype was not seen when we examined the total cells in the culture; rather, it was only seen in TH-positive dopaminergic

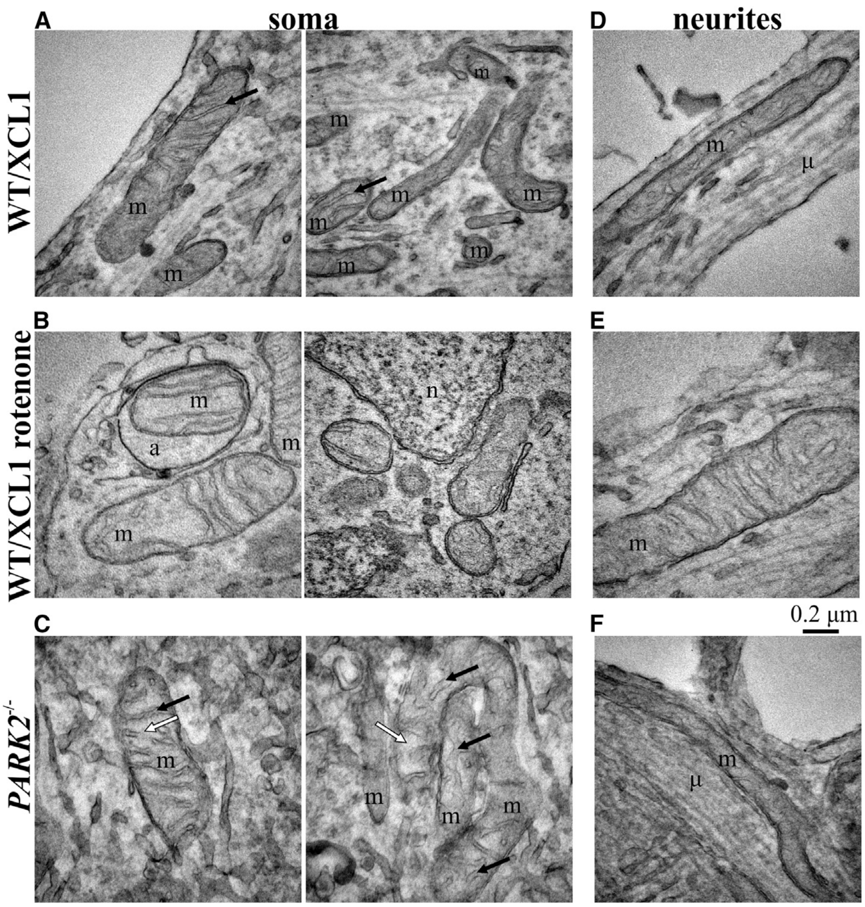

Figure 7. Mitochondrial Ultrastructure in Dopaminergic Neurons

Transmission electronmicrographs of dopaminergic neuronal cultures were recorded at ×68,000 magnification in 70-nm thick sections.

(A–C) Representative images of mitochondria (m) in neuronal somata.

(D–F) Representative images of mitochondria in neurites identified by parallel organized microtubule tracts (μ) (A and D) WT dopaminergic neurons, (B and E) rotenone-treated (100 μM, 24 hr) dopaminergic neurons, and (C and F) *PARK2*$^{-/-}$ neurons. Solid arrows point cristae of the inner mitochondrial membrane; open arrows mark the mitochondrial matrix. (E and F) (a), mitophagic vacuole; (n), nucleus. Results are representative of three independent experimental replicates.

neurons, which represented only a small percentage of the total cells (<30%). This may explain the apparent discrepancy with an earlier report on two PARK2 iPSC lines when mtDNA copy number was determined in the mix culture (Jiang et al., 2012). These results also suggested that NSC, astrocytes, and other cell populations may not show a significant phenotype, and this was confirmed in our whole genome analysis of NSC and astrocyte samples (see Results; data not shown).

Of importance was our finding of changes in other PD-associated genes, including SNCA (Krüger et al., 1998; Polymeropoulos et al., 1997; Singleton et al., 2003; Zarranz et al., 2004). SNCA is a presynaptic protein and function in regulating synaptic vesicle and neurotransmitter release. Aggregates of SNCA have been identified within the cytoplasmic inclusions (Lewy bodies) along with PARK2 in the brains of PD patients. It has been suggested that PARK2-mediated ubiquitination regulates SNCA assembly into ubiquitin-positive cytosolic inclusions, lending support for absence of these inclusions in PD patients with *PARK2* mutation (Chung et al., 2001). Here, we report an increase in SNCA protein levels in patient-derived neurons concomitant with a decrease in TH-positive cells. Similar correlation was reported previously to be associated with the aging process (Chu and Kordower, 2007). Although, some cellular and tissue studies in PD patients argue against the excess of SNCA in pathogenesis of PD (Dächsel et al., 2007), recent studies reaffirm the increase in SNCA protein levels in iPSC-derived neurons from patient with *PARK2* mutation (Imaizumi et al., 2012). It is possible that this increase in SNCA expression is an early event in the disease process and that patients with a late stage of PD do not display this phenotype. We acknowledge that there are many differences between a cell culture model and what may be seen in a culture dish. One operating assumption is that loss of PARK2 may lead to reduced processing of SNCA-associated proteins in particular SYNPHILIN; other data suggest that it is a nonclassical pathway (Chung et al., 2001; Lim et al., 2005; Sherer et al., 2003b; Zhang et al., 2013). We believe that altered ratios of interacting proteins may lead to either an increase or decrease depending on the stage of the disease. However, it is difficult to mimic the exact disease stage in culture just as it has been hard to

do so in rodents in vivo. Nevertheless, our data were consistent in all PARK2 patient lines in vitro.

We did not examine the presence of inclusion bodies or association of SNCA with these aggregates. In our culture, we did not observe a selective expression of SNCA in TH-positive cells, but rather, the expression was more random. In the absence of an appropriate reporter line, we could not infer from our data whether the TH-positive and SNCA-negative cells are surviving cells or that SNCA upregulation is delayed in these cells.

PARK2 is a ubiquitously expressed protein, and its ubiquitination of outer mitochondrial membrane is a prerequisite step in mitophagy-mediated removal of damaged mitochondria. However, PARK2 abnormalities in cells other than neurons fail to display the selective loss of a particular population of cells, suggesting that dysfunctional mitophagy could be compensated or delayed. Both PD patients and *PARK2* KO dopaminergic neurons display upregulation of several key mitophagy-associated proteins, as determined by our array data. Similarly, in our isogenic lines, the expression of these mitophagy-related genes displayed alleleic dependency and stage specificity. We identified a number cell death-inducing genes that were upregulated in dopaminergic neurons derived from PARK2 patients and *PARK2* KO lines; these include BID, BAX, BIM, BAK, PUMA, NOXA, BNIP3, and NIK (BCL-2 interacting killer). Although the mechanism by which dysfunctional mitophagy contribute to PD pathogenesis remain to be investigated, here we show that for the first time that PARK2 contributes to mitochondrial mass (volume) in dopaminergic neurons. We show that TH-positive neurons in PD patient and *PARK2* KO lines have a reduced mitochondrial mass compared with controls. A decrease in population of mitochondria within these TH-positive cells would shift the balance between healthy and defective mitochondrial and render these cells more vulnerable to accumulation of damaged mitochondria. We did not observe this alteration in the absence of *PARK2* mutations.

Given the ubiquitous expression of PARK2 and the changes we observed in our mixed dopaminergic neuron cultures, as well as previously published reports of observable phenotypes in cell lines unrelated to neurons (da Costa et al., 2009; Tsai et al., 2003), we reasoned that a subset of these changes may be seen in other neurons other than dopaminergic neurons. We took advantage of a neuronal differentiation system that we have developed (Liu et al., 2013) and examined a pure population of neurons of *PARK2* mutants. We focused our analysis on isogenic PARK2 lines as a more sensitive model of the PARK2 phenotype. Similar changes were seen, as with dopaminergic neurons. We saw a gradual decline in the number of surviving neurons in culture to approximately half of that in the isogenic control sample. This phenotypic change was consistent with previous observations in mouse models, which showed a decreased survival in response to stress (Sherer et al., 2003b; Testa et al., 2005). Comparison of the mitochondrial and cell death gene changes showed a similar but not identical profile. These results along with the lack of a phenotype in iPSC, NSC, and astrocytes highlight the importance of studying the effect in an appropriate cellular context. Our observation that the phenotype can be studied in generic neurons provides a feasible assay with additional stress using a purified population of cells that can be obtained 2- to 3-fold faster and with much less effort than authentic midbrain dopaminergic neurons.

Overall, our results provide a current model of PARK2 function where damaged mitochondria are targeted for degradation via a PARK2/PINK1 interaction. Loss of PARK2 results in an initial accumulation of damaged mitochondria, and stress in culture results in a slow reduction in cell number as internal repair process fail to compensate for loss of metabolic activity. Cells with a higher metabolic activity are more susceptible to suffer mitochondrial loss and display a phenotype earlier than more robust glial cells. Using multiple lines and generating isogenic controls and combining phenotypic changes with gene expression profiling provide a useful model for elucidating pathways underlying the disease process and provide important tools that are useful for the PD community. These results also suggest that the engineered PARK2 KO and it isogenic controls provide a valuable model to assess familial PD models and to construct single- and double-mutant models.

EXPERIMENTAL PROCEDURES

Generation of Patient-Specific and Isogenic iPSC Lines

PD patient fibroblasts were obtained from Coriell. Fibroblasts growing conditions, reprogramming, and differentiation procedures are described in the Supplemental Information.

The isogenic *PARK2* KO lines were generated by ZFN technology and were obtained from XCell Science. The detailed method was described in the Supplemental Information.

Neural and Dopaminergic Neuronal Differentiation

Generation of NSC and dopaminergic differentiation from NSC was described (Swistowski et al., 2009). The detailed procedures were seen in the Supplemental Information.

Immunocytochemistry and Western Blot

Immunocytochemistry and western blot procedures were as described previously (Zeng et al., 2003). See Supplemental Information for used antibodies and further descriptions.

Microarray and qPCR Analyses

Total RNA was isolated using the RNeasy Mini kit according to the manufacturer's instructions (QIAGEN) and hybridized to Illumina

Human HT-12 BeadChip (Illumina, performed by Microarray core facility at the Burnham Institute for Medical Research). All of the data processing and analysis were performed using the algorithms included with the Illumina BeadStudio software, and further description can be found in Supplemental Information. The qPCR procedure was described in the Supplemental Information. The GEO accession number for microarray data is GSE66241.

MtDNA Copy Number Assessment

The mtDNA copy number was determined by comparing PCR amplification of a mitochondrial amplicon (human, NADH-ubiquinone oxidoreductase chain 5 [ND5]) with a nuclear amplicon (human, cystic fibrosis) (Wong and Cortopassi, 2002). The standard curves were generated for quantifications obtained by amplification curves of nuclear cystic fibrosis gene and ND5 gene amplified from 0- to 100-ng and 0- to 1-ng K562 DNA, respectively.

Confocal Microscopic Stereology of Mitochondria Volume Fraction

Mitochondria: cell volume fractions (V_F) were determined using a confocal microscopy and image processing-based stereologic approach according to (Gerencser et al., 2012) and are further described in Supplemental Information.

Electron Microscopy

Transmission electron microscopy was performed as previously described (Birket et al., 2011), and the detail was described in the Supplemental Information.

Statistical Analysis

Statistical analyses were performed using two-tailed paired or unpaired when analyzing isogenic lines. For patient-derived cell lines, the statistical significance was calculated from a one way ANOVA using Dunnett's correction. *$p < 0.05$, **$p < 0.01$.

ACCESSION NUMBERS

The microarray data utilized in this study were deposited under the GEO accession number GSE66241.

SUPPLEMENTAL INFORMATION

Supplemental Information includes Supplemental Experimental Procedures, two figures, and six tables and can be found with this article online at http://dx.doi.org/10.1016/j.stemcr.2015.02.019.

AUTHOR CONTRIBUTIONS

A.S. performed differentiation and mitochondrial function experiments. R.S. generated some patient iPSC lines and performed microarray analysis. Y.P. generated some isogenic Park2 iPSC lines.

ACKNOWLEDGMENTS

This work was supported in part by California Institute for Regenerative Medicine Grants TR-01856 (Zeng) and TG2-01155 (Zeng) to X.Z. We thank former and current Zeng lab members for technical assistant and helpful discussions.

Received: September 26, 2014
Revised: February 23, 2015
Accepted: February 25, 2015
Published: April 2, 2015

REFERENCES

Abbas, N., Lücking, C.B., Ricard, S., Dürr, A., Bonifati, V., De Michele, G., Bouley, S., Vaughan, J.R., Gasser, T., Marconi, R., et al.; French Parkinson's Disease Genetics Study Group and the European Consortium on Genetic Susceptibility in Parkinson's Disease (1999). A wide variety of mutations in the parkin gene are responsible for autosomal recessive parkinsonism in Europe. Hum. Mol. Genet. *8*, 567–574.

Akundi, R.S., Zhi, L., Sullivan, P.G., and Büeler, H. (2013). Shared and cell type-specific mitochondrial defects and metabolic adaptations in primary cells from PINK1-deficient mice. Neurodegener. Dis. *12*, 136–149.

Anderson, S., Bankier, A.T., Barrell, B.G., de Bruijn, M.H., Coulson, A.R., Drouin, J., Eperon, I.C., Nierlich, D.P., Roe, B.A., Sanger, F., et al. (1981). Sequence and organization of the human mitochondrial genome. Nature *290*, 457–465.

Birket, M.J., Orr, A.L., Gerencser, A.A., Madden, D.T., Vitelli, C., Swistowski, A., Brand, M.D., and Zeng, X. (2011). A reduction in ATP demand and mitochondrial activity with neural differentiation of human embryonic stem cells. J. Cell Sci. *124*, 348–358.

Burman, J.L., Yu, S., Poole, A.C., Decal, R.B., and Pallanck, L. (2012). Analysis of neural subtypes reveals selective mitochondrial dysfunction in dopaminergic neurons from parkin mutants. Proc. Natl. Acad. Sci. USA *109*, 10438–10443.

Cathomen, T., and Joung, J.K. (2008). Zinc-finger nucleases: the next generation emerges. Mol. Ther. *16*, 1200–1207.

Chauvin, C., De Oliveira, F., Ronot, X., Mousseau, M., Leverve, X., and Fontaine, E. (2001). Rotenone inhibits the mitochondrial permeability transition-induced cell death in U937 and KB cells. J. Biol. Chem. *276*, 41394–41398.

Chen, H., and Chan, D.C. (2009). Mitochondrial dynamics—fusion, fission, movement, and mitophagy—in neurodegenerative diseases. Hum. Mol. Genet. *18* (R2), R169–R176.

Chu, Y., and Kordower, J.H. (2007). Age-associated increases of alpha-synuclein in monkeys and humans are associated with nigrostriatal dopamine depletion: Is this the target for Parkinson's disease? Neurobiol. Dis. *25*, 134–149.

Chung, K.K., Zhang, Y., Lim, K.L., Tanaka, Y., Huang, H., Gao, J., Ross, C.A., Dawson, V.L., and Dawson, T.M. (2001). Parkin ubiquitinates the alpha-synuclein-interacting protein, synphilin-1: implications for Lewy-body formation in Parkinson disease. Nat. Med. *7*, 1144–1150.

Clark, I.E., Dodson, M.W., Jiang, C., Cao, J.H., Huh, J.R., Seol, J.H., Yoo, S.J., Hay, B.A., and Guo, M. (2006). Drosophila pink1 is required for mitochondrial function and interacts genetically with parkin. Nature *441*, 1162–1166.

da Costa, C.A., Sunyach, C., Giaime, E., West, A., Corti, O., Brice, A., Safe, S., Abou-Sleiman, P.M., Wood, N.W., Takahashi, H., et al. (2009). Transcriptional repression of p53 by parkin and impairment by mutations associated with autosomal recessive juvenile Parkinson's disease. Nat. Cell Biol. *11*, 1370–1375.

Dächsel, J.C., Lincoln, S.J., Gonzalez, J., Ross, O.A., Dickson, D.W., and Farrer, M.J. (2007). The ups and downs of alpha-synuclein mRNA expression. Mov. Disord. *22*, 293–295.

Dauer, W., and Przedborski, S. (2003). Parkinson's disease: mechanisms and models. Neuron *39*, 889–909.

Dawson, T.M. (2006). Parkin and defective ubiquitination in Parkinson's disease. J. Neural Transm. Suppl. *7*, 209–213.

Dawson, T.M., Ko, H.S., and Dawson, V.L. (2010). Genetic animal models of Parkinson's disease. Neuron *66*, 646–661.

Ekstrand, M.I., Terzioglu, M., Galter, D., Zhu, S., Hofstetter, C., Lindqvist, E., Thams, S., Bergstrand, A., Hansson, F.S., Trifunovic, A., et al. (2007). Progressive parkinsonism in mice with respiratory-chain-deficient dopamine neurons. Proc. Natl. Acad. Sci. USA *104*, 1325–1330.

Exner, N., Lutz, A.K., Haass, C., and Winklhofer, K.F. (2012). Mitochondrial dysfunction in Parkinson's disease: molecular mechanisms and pathophysiological consequences. EMBO J. *31*, 3038–3062.

Geisler, S., Holmström, K.M., Skujat, D., Fiesel, F.C., Rothfuss, O.C., Kahle, P.J., and Springer, W. (2010). PINK1/Parkin-mediated mitophagy is dependent on VDAC1 and p62/SQSTM1. Nat. Cell Biol. *12*, 119–131.

Gerencser, A.A., Chinopoulos, C., Birket, M.J., Jastroch, M., Vitelli, C., Nicholls, D.G., and Brand, M.D. (2012). Quantitative measurement of mitochondrial membrane potential in cultured cells: calcium-induced de- and hyperpolarization of neuronal mitochondria. J. Physiol. *590*, 2845–2871.

Goldberg, M.S., Fleming, S.M., Palacino, J.J., Cepeda, C., Lam, H.A., Bhatnagar, A., Meloni, E.G., Wu, N., Ackerson, L.C., Klapstein, G.J., et al. (2003). Parkin-deficient mice exhibit nigrostriatal deficits but not loss of dopaminergic neurons. J. Biol. Chem. *278*, 43628–43635.

Greene, J.C., Whitworth, A.J., Kuo, I., Andrews, L.A., Feany, M.B., and Pallanck, L.J. (2003). Mitochondrial pathology and apoptotic muscle degeneration in Drosophila parkin mutants. Proc. Natl. Acad. Sci. USA *100*, 4078–4083.

Henchcliffe, C., and Beal, M.F. (2008). Mitochondrial biology and oxidative stress in Parkinson disease pathogenesis. Nat. Clin. Pract. Neurol. *4*, 600–609.

Imaizumi, Y., Okada, Y., Akamatsu, W., Koike, M., Kuzumaki, N., Hayakawa, H., Nihira, T., Kobayashi, T., Ohyama, M., Sato, S., et al. (2012). Mitochondrial dysfunction associated with increased oxidative stress and α-synuclein accumulation in PARK2 iPSC-derived neurons and postmortem brain tissue. Mol. Brain *5*, 35.

Jiang, H., Ren, Y., Yuen, E.Y., Zhong, P., Ghaedi, M., Hu, Z., Azabdaftari, G., Nakaso, K., Yan, Z., and Feng, J. (2012). Parkin controls dopamine utilization in human midbrain dopaminergic neurons derived from induced pluripotent stem cells. Nat Commun *3*, 668.

Johns, D.R. (1995). Seminars in medicine of the Beth Israel Hospital, Boston. Mitochondrial DNA and disease. N. Engl. J. Med. *333*, 638–644.

Kim, Y., Park, J., Kim, S., Song, S., Kwon, S.K., Lee, S.H., Kitada, T., Kim, J.M., and Chung, J. (2008). PINK1 controls mitochondrial localization of Parkin through direct phosphorylation. Biochem. Biophys. Res. Commun. *377*, 975–980.

Kitada, T., Asakawa, S., Hattori, N., Matsumine, H., Yamamura, Y., Minoshima, S., Yokochi, M., Mizuno, Y., and Shimizu, N. (1998). Mutations in the parkin gene cause autosomal recessive juvenile parkinsonism. Nature *392*, 605–608.

Krüger, R., Kuhn, W., Müller, T., Woitalla, D., Graeber, M., Kösel, S., Przuntek, H., Epplen, J.T., Schöls, L., and Riess, O. (1998). Ala30Pro mutation in the gene encoding alpha-synuclein in Parkinson's disease. Nat. Genet. *18*, 106–108.

Lee, Y.J., Jeong, S.Y., Karbowski, M., Smith, C.L., and Youle, R.J. (2004). Roles of the mammalian mitochondrial fission and fusion mediators Fis1, Drp1, and Opa1 in apoptosis. Mol. Biol. Cell *15*, 5001–5011.

Lim, K.L., Chew, K.C., Tan, J.M., Wang, C., Chung, K.K., Zhang, Y., Tanaka, Y., Smith, W., Engelender, S., Ross, C.A., et al. (2005). Parkin mediates nonclassical, proteasomal-independent ubiquitination of synphilin-1: implications for Lewy body formation. J. Neurosci. *25*, 2002–2009.

Lin, T.K., Cheng, C.H., Chen, S.D., Liou, C.W., Huang, C.R., and Chuang, Y.C. (2012). Mitochondrial Dysfunction and Oxidative Stress Promote Apoptotic Cell Death in the Striatum via Cytochrome c/Caspase-3 Signaling Cascade Following Chronic Rotenone Intoxication in Rats. Int. J. Mol. Sci. *13*, 8722–8739.

Liu, Q., Pedersen, O.Z., Peng, J., Couture, L.A., Rao, M.S., and Zeng, X. (2013). Optimizing dopaminergic differentiation of pluripotent stem cells for the manufacture of dopaminergic neurons for transplantation. Cytotherapy *15*, 999–1010.

Momčilović, O., Liu, Q., Swistowski, A., Russo-Tait, T., Zhao, Y., Rao, M.S., and Zeng, X. (2014). Genome wide profiling of dopaminergic neurons derived from human embryonic and induced pluripotent stem cells. Stem Cells Dev. *23*, 406–420.

Mortiboys, H., Thomas, K.J., Koopman, W.J., Klaffke, S., Abou-Sleiman, P., Olpin, S., Wood, N.W., Willems, P.H., Smeitink, J.A., Cookson, M.R., and Bandmann, O. (2008). Mitochondrial function and morphology are impaired in parkin-mutant fibroblasts. Ann. Neurol. *64*, 555–565.

Panov, A., Dikalov, S., Shalbuyeva, N., Taylor, G., Sherer, T., and Greenamyre, J.T. (2005). Rotenone model of Parkinson disease: multiple brain mitochondria dysfunctions after short term systemic rotenone intoxication. J. Biol. Chem. *280*, 42026–42035.

Pavletich, N.P., and Pabo, C.O. (1991). Zinc finger-DNA recognition: crystal structure of a Zif268-DNA complex at 2.1 A. Science *252*, 809–817.

Perier, C., Bender, A., García-Arumí, E., Melià, M.J., Bové, J., Laub, C., Klopstock, T., Elstner, M., Mounsey, R.B., Teismann, P., et al. (2013). Accumulation of mitochondrial DNA deletions within dopaminergic neurons triggers neuroprotective mechanisms. Brain *136*, 2369–2378.

Polymeropoulos, M.H., Lavedan, C., Leroy, E., Ide, S.E., Dehejia, A., Dutra, A., Pike, B., Root, H., Rubenstein, J., Boyer, R., et al. (1997). Mutation in the alpha-synuclein gene identified in families with Parkinson's disease. Science *276*, 2045–2047.

Reeve, A., Meagher, M., Lax, N., Simcox, E., Hepplewhite, P., Jaros, E., and Turnbull, D. (2013). The impact of pathogenic mitochondrial DNA mutations on substantia nigra neurons. J. Neurosci. *33*, 10790–10801.

Rothfuss, O., Fischer, H., Hasegawa, T., Maisel, M., Leitner, P., Miesel, F., Sharma, M., Bornemann, A., Berg, D., Gasser, T., and Patenge, N. (2009). Parkin protects mitochondrial genome integrity and supports mitochondrial DNA repair. Hum. Mol. Genet. *18*, 3832–3850.

Schapira, A.H., Cooper, J.M., Dexter, D., Jenner, P., Clark, J.B., and Marsden, C.D. (1989). Mitochondrial complex I deficiency in Parkinson's disease. Lancet *1*, 1269.

Sherer, T.B., Betarbet, R., Testa, C.M., Seo, B.B., Richardson, J.R., Kim, J.H., Miller, G.W., Yagi, T., Matsuno-Yagi, A., and Greenamyre, J.T. (2003a). Mechanism of toxicity in rotenone models of Parkinson's disease. J. Neurosci. *23*, 10756–10764.

Sherer, T.B., Kim, J.H., Betarbet, R., and Greenamyre, J.T. (2003b). Subcutaneous rotenone exposure causes highly selective dopaminergic degeneration and alpha-synuclein aggregation. Exp. Neurol. *179*, 9–16.

Shiba-Fukushima, K., Imai, Y., Yoshida, S., Ishihama, Y., Kanao, T., Sato, S., and Hattori, N. (2012). PINK1-mediated phosphorylation of the Parkin ubiquitin-like domain primes mitochondrial translocation of Parkin and regulates mitophagy. Sci Rep *2*, 1002.

Singleton, A.B., Farrer, M., Johnson, J., Singleton, A., Hague, S., Kachergus, J., Hulihan, M., Peuralinna, T., Dutra, A., Nussbaum, R., et al. (2003). alpha-Synuclein locus triplication causes Parkinson's disease. Science *302*, 841.

Sulzer, D. (2007). Multiple hit hypotheses for dopamine neuron loss in Parkinson's disease. Trends Neurosci. *30*, 244–250.

Swistowski, A., Peng, J., Han, Y., Swistowska, A.M., Rao, M.S., and Zeng, X. (2009). Xeno-free defined conditions for culture of human embryonic stem cells, neural stem cells and dopaminergic neurons derived from them. PLoS ONE *4*, e6233.

Tanaka, K., Suzuki, T., Hattori, N., and Mizuno, Y. (2004). Ubiquitin, proteasome and parkin. Biochim. Biophys. Acta *1695*, 235–247.

Testa, C.M., Sherer, T.B., and Greenamyre, J.T. (2005). Rotenone induces oxidative stress and dopaminergic neuron damage in organotypic substantia nigra cultures. Brain Res. Mol. Brain Res. *134*, 109–118.

Tsai, Y.C., Fishman, P.S., Thakor, N.V., and Oyler, G.A. (2003). Parkin facilitates the elimination of expanded polyglutamine proteins and leads to preservation of proteasome function. J. Biol. Chem. *278*, 22044–22055.

Urnov, F.D., Rebar, E.J., Holmes, M.C., Zhang, H.S., and Gregory, P.D. (2010). Genome editing with engineered zinc finger nucleases. Nat. Rev. Genet. *11*, 636–646.

van der Bliek, A.M., Shen, Q., and Kawajiri, S. (2013). Mechanisms of mitochondrial fission and fusion. Cold Spring Harb. Perspect. Biol. *5*.

Vila, M., Ramonet, D., and Perier, C. (2008). Mitochondrial alterations in Parkinson's disease: new clues. J. Neurochem. *107*, 317–328.

Wang, T., Choi, E., Monaco, M.C., Campanac, E., Medynets, M., Do, T., Rao, P., Johnson, K.R., Elkahloun, A.G., Von Geldern, G., et al. (2013). Derivation of neural stem cells from human adult peripheral CD34+ cells for an autologous model of neuroinflammation. PLoS ONE *8*, e81720.

Wei, Y.H. (1998). Mitochondrial DNA mutations and oxidative damage in aging and diseases: an emerging paradigm of gerontology and medicine. Proc. Natl. Sci. Counc. Repub. China B *22*, 55–67.

Wong, A., and Cortopassi, G. (2002). Reproducible quantitative PCR of mitochondrial and nuclear DNA copy number using the LightCycler. Methods Mol. Biol. *197*, 129–138.

Yadava, N., and Nicholls, D.G. (2007). Spare respiratory capacity rather than oxidative stress regulates glutamate excitotoxicity after partial respiratory inhibition of mitochondrial complex I with rotenone. J. Neurosci. *27*, 7310–7317.

Yang, W., Mills, J.A., Sullivan, S., Liu, Y., French, D.L., and Gadue, P. (2008). iPSC Reprogramming from Human Peripheral Blood Using Sendai Virus Mediated Gene Transfer. http://www.stembook.org/sites/default/files/protocols/iPSC_Reprogramming_from_Human_Peripheral_Blood_0.pdf.

Zarranz, J.J., Alegre, J., Gómez-Esteban, J.C., Lezcano, E., Ros, R., Ampuero, I., Vidal, L., Hoenicka, J., Rodriguez, O., Atarés, B., et al. (2004). The new mutation, E46K, of alpha-synuclein causes Parkinson and Lewy body dementia. Ann. Neurol. *55*, 164–173.

Zeng, X., Chen, J., Sanchez, J.F., Coggiano, M., Dillon-Carter, O., Petersen, J., and Freed, W.J. (2003). Stable expression of hrGFP by mouse embryonic stem cells: promoter activity in the undifferentiated state and during dopaminergic neural differentiation. Stem Cells *21*, 647–653.

Zeng, X., Hunsberger, J.G., Simeonov, A., Malik, N., Pei, Y., and Rao, M. (2014). Concise review: modeling central nervous system diseases using induced pluripotent stem cells. Stem Cells Transl Med *3*, 1418–1428.

Zhang, Y., Ma, H., Xie, B., Han, C., Wang, C., Qing, H., and Deng, Y. (2013). Alpha-synuclein overexpression induced mitochondrial damage by the generation of endogenous neurotoxins in PC12 cells. Neurosci. Lett. *547*, 65–69.

Stem Cell Reports, Volume *4*

Supplemental Information

Mitochondrial Alterations by PARKIN in Dopaminergic Neurons Using PARK2 Patient-Specific and *PARK2* Knockout Isogenic iPSC Lines

Atossa Shaltouki, Renuka Sivapatham, Ying Pei, Akos A. Gerencser, Olga Momčilović, Mahendra S. Rao, and Xianmin Zeng

Supplemental Figure 1.

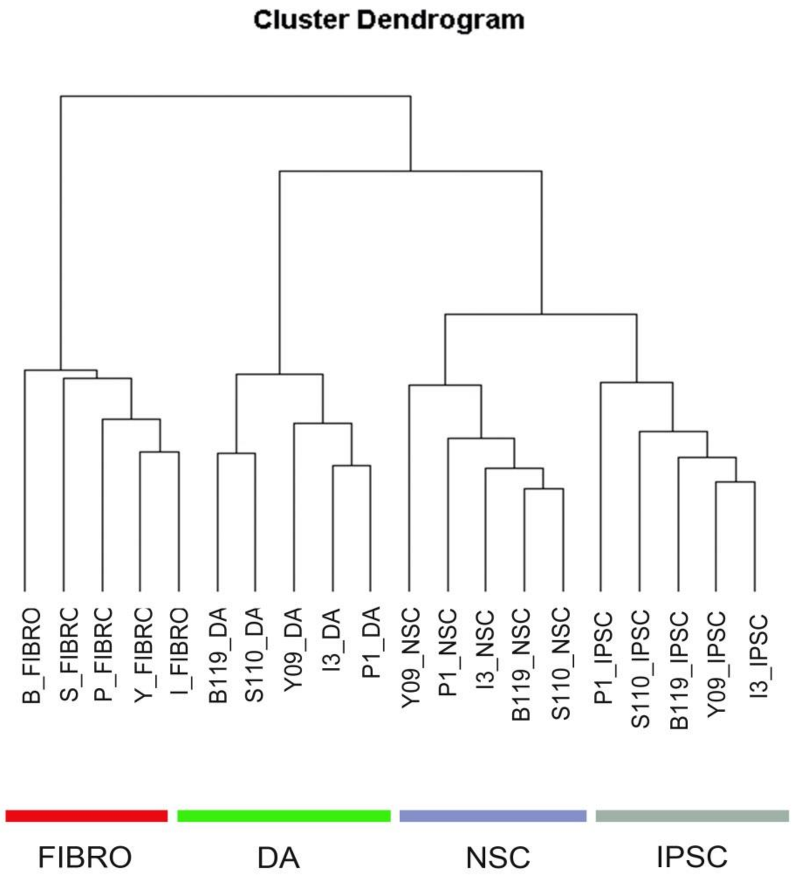

Sup Figure 1. Whole gene expression profiles of PARK2 and control lines at various stages of dopaminergic differentiation. The dendrogram confirms the clustering among iPSC lines, iPSC-derived NSC and iPSC-derived dopaminergic neurons. The horizontal axis of the dendrogram represents the dissimilarity between clusters in terms of gene expression pattern. The vertical axis represents clusters. Abbreviations: F: Fibroblast; iPSC: Induced pluripotent stem cells; NSC: neural stem cells; DA: dopaminergic neurons.

Supplemental Figure 2.

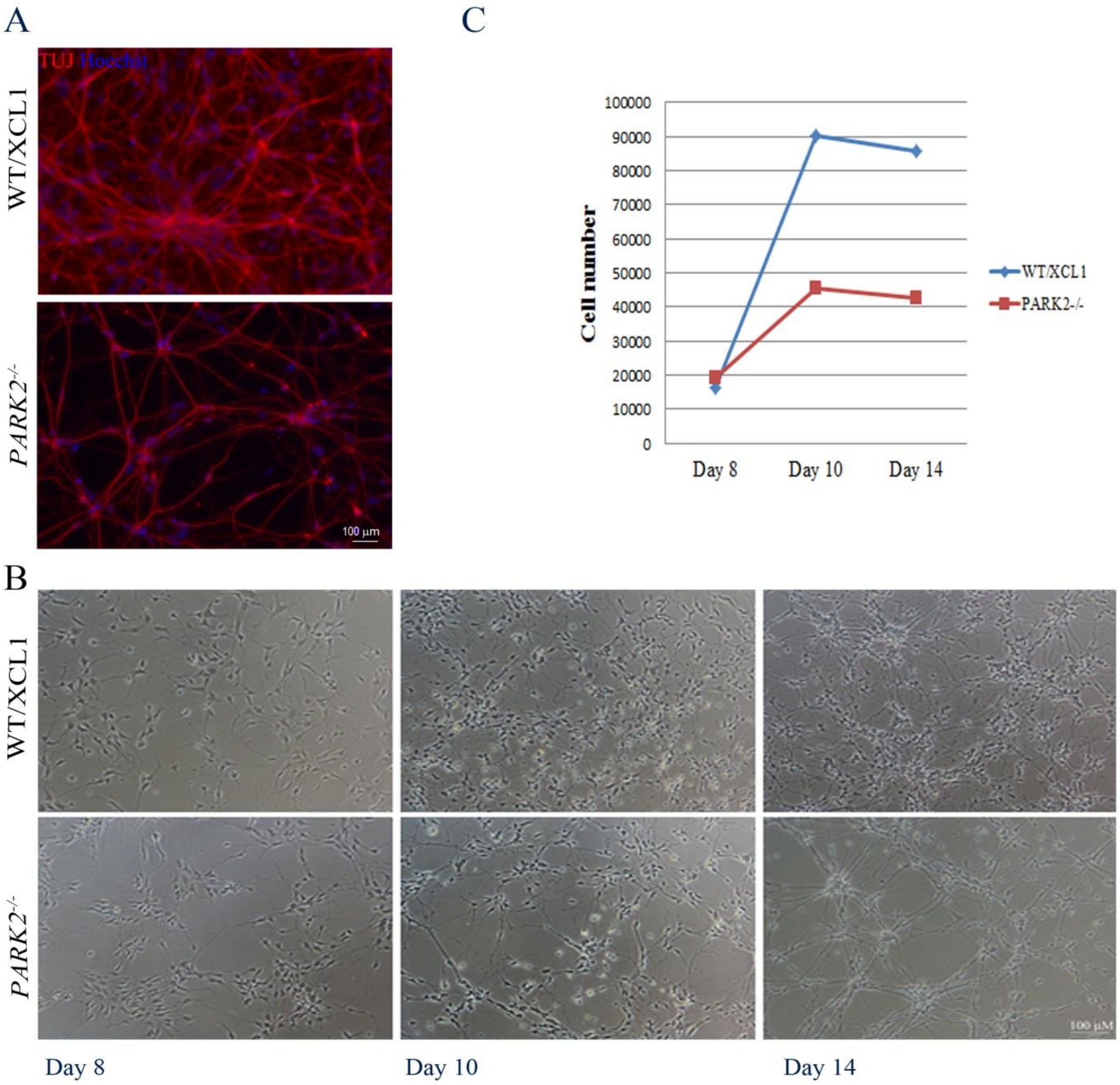

Sup Figure 2. *PARK2$^{-/-}$* neurons appeared to be more stressed compared to their isogenic control neurons. (A) Generation of a pure population of neurons from the *PARK2* isogenic lines. More than 95% of total cells expressed TUJ-1 after 14 days of differentiation (time of assay) in both WT and *PARK2$^{-/-}$* lines. (B) Morphology of neuronal cells at Day 8, 10 and 14 of Park2 KO and the control WT cells. (C) Neuronal cell count at various time points. WT cell showed a higher rate of cell proliferation when

compared to control *PARK2*$^{-/-}$ measured by the MTT assay. Results are representative of three independent experiments.

Supplemental Table 1. List of PARK2 patient and control cells used in the study.

Line	NINDS Catalog ID	Mutation	Gender	Race	Age of onset	Age of sample
PARK2	ND30171 (P)	Park2: R42P<br>Park2: EX3DEL	Male	Caucasian	42	54
	ND29543 (I)	Park2: EX3-4DEL<br>Park2: 1-BP DEL, 255A	Male	Hispanic	16	50
	ND29369 (B)	Park2: R275W	Female	Hispanic	43	61
	ND31618 (S)	Park2: R42P	Female	Caucasian	44	63
Control	ND34791 (Y)	Population control	Female	Caucasian	n/a	60

Supplemental Table 2. Expression of PD genes in Fibroblast and iPSC patient lines.

SYMBOL	P Fibro	I Fibro	B Fibro	S Fibro	Y Fibro	P1 iPSC	I3 iPSC	B119 iPSC	S110 iPSC	Y9 iPSC
ATP13A2	384	270	245	249	303	172	304	334	411	178
ATXN2	4175	3345	3041	3396	4208	2309	3897	3112	2584	3785
BST1	691	884	169	323	281	-2	25	30	5	-5
EIF4G1	2725	1331	1458	1188	2039	1534	1235	1389	1577	1212
FBXO7	479	602	709	734	516	305	459	465	490	458
FGF20	20	23	18	17	28	35	4	55	40	31
GAK	867	472	571	493	576	506	502	401	391	630
GBA	1738	1759	1465	2907	2013	361	533	271	540	380
GIGYF2	14	40	27	11	47	38	51	34	32	47
GPNMB	642	1030	790	1177	1312	80	6	71	20	33
HTRA2	666	620	786	780	581	309	296	430	300	348
MC1R	464	652	646	441	375	287	117	106	177	164
MCCC1	689	753	641	828	618	1163	967	989	998	1113
PARK7	15260	16223	16048	15681	15538	15594	16395	19195	16467	17639
PDXK	2332	1455	1645	1621	1709	2091	1386	1662	1323	1253
PINK1	1925	1789	1619	1967	1419	231	364	265	464	414
PM20D1	42	66	43	74	51	64	55	32	10	28
RAB25	-10	35	8	20	30	846	1508	1722	1825	2147
SETD1A	786	420	595	397	434	413	573	714	529	573
SNCA	74	96	37	115	76	676	617	1099	872	1129
STK39	1800	2220	2393	1542	1958	1455	1793	2208	2103	1750
TBP	758	794	948	762	795	1198	1174	1413	1190	1182
UCHL1	740	3420	2045	3740	3359	15075	19112	19483	17539	15232
VPS35	3434	3816	4161	4053	3465	2728	3083	2005	2271	2841

Supplemental Table 3. R^2 of all patient line.

R^2	Y FIBRO	Y9 IPSC	Y09 NSC	Y09 DA2	B FIBRO	B119 IPSC	B119 NSC	B119 DA2	I FIBRO	I3 IPSC	I3 NSC	I3 DA2	P FIBRO	P1 IPSC	P1 NSC	P1 DA2	S FIBRO	S110 IPSC	S110 NSC	S110 DA2
Y FIBRO	1	0.86	0.88	0.84	0.96	0.86	0.86	0.8	0.98	0.85	0.86	0.86	0.97	0.86	0.87	0.85	0.97	0.86	0.87	0.82
Y9 IPSC	0.86	1	0.95	0.91	0.88	0.98	0.95	0.86	0.86	0.99	0.95	0.92	0.83	0.97	0.95	0.92	0.86	0.98	0.95	0.89
Y09 NSC	0.88	0.95	1	0.94	0.9	0.95	0.97	0.89	0.89	0.94	0.97	0.94	0.86	0.93	0.96	0.94	0.88	0.94	0.97	0.92
Y09 DA2	0.84	0.91	0.94	1	0.85	0.91	0.93	0.96	0.85	0.9	0.94	0.98	0.8	0.89	0.93	0.97	0.84	0.9	0.93	0.97
B FIBRO	0.96	0.88	0.9	0.85	1	0.88	0.89	0.82	0.97	0.87	0.89	0.87	0.96	0.86	0.89	0.86	0.96	0.88	0.89	0.84
B119 IPSC	0.86	0.98	0.95	0.91	0.88	1	0.96	0.86	0.87	0.98	0.96	0.92	0.83	0.97	0.95	0.92	0.86	0.98	0.96	0.89
B119 NSC	0.86	0.95	0.97	0.93	0.89	0.96	1	0.9	0.87	0.94	0.99	0.95	0.83	0.93	0.98	0.95	0.86	0.95	0.99	0.93
B119 DA2	0.8	0.86	0.89	0.96	0.82	0.86	0.9	1	0.8	0.86	0.91	0.96	0.78	0.84	0.89	0.96	0.79	0.87	0.9	0.98
I FIBRO	0.98	0.86	0.89	0.85	0.97	0.87	0.87	0.8	1	0.86	0.87	0.86	0.98	0.85	0.87	0.85	0.98	0.86	0.87	0.82
I3 IPSC	0.85	0.99	0.94	0.9	0.87	0.98	0.94	0.86	0.86	1	0.95	0.92	0.83	0.98	0.94	0.91	0.85	0.98	0.95	0.89
I3 NSC	0.86	0.95	0.97	0.94	0.89	0.96	0.99	0.91	0.87	0.95	1	0.96	0.83	0.94	0.98	0.96	0.86	0.95	0.99	0.94
I3 DA2	0.86	0.92	0.94	0.98	0.87	0.92	0.95	0.96	0.86	0.92	0.96	1	0.83	0.91	0.95	0.99	0.85	0.92	0.95	0.98
P FIBRO	0.97	0.83	0.86	0.8	0.96	0.83	0.83	0.78	0.98	0.83	0.83	0.83	1	0.82	0.83	0.81	0.96	0.84	0.84	0.8
P1 IPSC	0.86	0.97	0.93	0.89	0.86	0.97	0.93	0.84	0.85	0.98	0.94	0.91	0.82	1	0.95	0.91	0.84	0.96	0.94	0.87
P1 NSC	0.87	0.95	0.96	0.93	0.89	0.95	0.98	0.89	0.87	0.94	0.98	0.95	0.83	0.95	1	0.95	0.86	0.94	0.98	0.92
P1 DA2	0.85	0.92	0.94	0.97	0.86	0.92	0.95	0.96	0.85	0.91	0.96	0.99	0.81	0.91	0.95	1	0.84	0.91	0.95	0.98
S FIBRO	0.97	0.86	0.88	0.84	0.96	0.86	0.86	0.79	0.98	0.85	0.86	0.85	0.96	0.84	0.86	0.84	1	0.85	0.86	0.82
S110 IPSC	0.86	0.98	0.94	0.9	0.88	0.98	0.95	0.87	0.86	0.98	0.95	0.92	0.84	0.96	0.94	0.91	0.85	1	0.96	0.9
S110 NSC	0.87	0.95	0.97	0.93	0.89	0.96	0.99	0.9	0.87	0.95	0.99	0.95	0.84	0.94	0.98	0.95	0.86	0.96	1	0.93
S110 DA2	0.82	0.89	0.92	0.97	0.84	0.89	0.93	0.98	0.82	0.89	0.94	0.98	0.8	0.87	0.92	0.98	0.82	0.9	0.93	1

Supplemental Table 4. Mitochondrial and cell death-related genes.

Mitochondria genes

ND1	NDUFB1	CO2	COX7B2	ATP1B4	ATP5J	ATP6V1E1	UQCC
ND2	NDUFB10	C03	COX7C	ATP2A1	ATP5J2	ATP6V1E2	UQCR
ND3	NDUFB11	COX10	COX8A	ATP2A2	ATP5L	ATP6V1F	UQCRB
ND4	NDUFB2	COX11	COX8C	ATP2A3	ATP5O	ATP6V1G1	UQCRC1
ND4L	NDUFB3	COX11P	MT-ATP6	ATP2B1	ATP5S	ATP6V1G2	UQCRC2
ND5	NDUFB4	COX15	MT-ATP8	ATP2B2	ATP5SL	ATP6V1G3	UQCRFS1
ND6	NDUFB5	COX16	ATP10A	ATP2B3	ATP6AP1	ATP6V1H	UQCRH
NDUFA1	NDUFB6	COX17	ATP10B	ATP2B4	ATP6AP1L	ATP7A	UQCRHL
NDUFA10	NDUFB7	COX18	ATP10D	ATP2C1	ATP6AP2	ATP7B	UQCRQ
NDUFA11	NDUFB8	COX19	ATP11A	ATP2C2	ATP6V0A1	ATP8A1	CYC1
NDUFA12	NDUFB9	COX4I1	ATP11B	ATP4A	ATP6V0A2	ATP8A2	SDHA
NDUFA13	NDUFC1	COX4I2	ATP11C	ATP4B	ATP6V0A4	ATP8B1	SDHAF1
NDUFA2	NDUFC2	COX4NB	ATP12A	ATP5A1	ATP6V0B	ATP8B2	SDHAF2
NDUFA3	NDUFS1	COX5A	ATP13A1	ATP5B	ATP6V0C	ATP8B3	SDHALP1
NDUFA4	NDUFS2	COX5B	ATP13A2	ATP5C1	ATP6V0D1	ATP8B4	SDHAP2
NDUFA4L2	NDUFS3	COX6A1	ATP13A3	ATP5D	ATP6V0D2	ATP9A	SDHAP3
NDUFA5	NDUFS4	COX6A2	ATP13A4	ATP5E	ATP6V0E1	ATP9B	SDHB
NDUFA6	NDUFS5	COX6B1	ATP13A5	ATP5EP2	ATP6V0E2	ATPAF1	SDHC
NDUFA7	NDUFS6	COX6B2	ATP1A1	ATP5F1	ATP6V1A	ATPAF2	SDHD
NDUFA8	NDUFS7	COX6BP1	ATP1A2	ATP5G1	ATP6V1B1	ATPBD1B	GSK3A
NDUFA9	NDUFS8	COX6C	ATP1A3	ATP5G2	ATP6V1B2	ATPBD3	GSK3B
NDUFAB1	NDUFV1	COX7A1	ATP1A4	ATP5G3	ATP6V1C1	ATPBD4	UCP1
NDUFAF1	NDUFV2	COX7A2	ATP1B1	ATP5H	ATP6V1C2	ATPGD1	UCP2
NDUFAF2	NDUFV3	COX7A2L	ATP1B2	ATP5I	ATP6V1D	ATPIF1	UCP3
NDUFAF3	CO1	COX7B	ATP1B3				

Death genes

SLC25A31	BCL7B	PARK7	ATP6V0A4	ATG4B	GCM1	H2AFX	SNORD25
SLC25A4	BCL7C	PDDC1	ATP6V0B	LOC644284	SNORD48	LOC729057	SDK2
SLC25A5	BCL8	PACRG	ATP6V0C	NAMPT	LOC650034	RPL12P6	STT3B
SLC25A6	BCL9	PACRGL	ATP6V0D1	LOC654201	RNU1F1	LOC728787	SRCAP
PPID	BCL9L	PINK1	ATP6V0D2	LOC649841	ISLR2	LOC645195	LOC100129685
VCY1B	BCLAF1	SNCA	ATP6V0E1	LOC653383	CDK5R2	TBX19	SUV420H1
VDAC1	HRK	SNCAIP	ATP6V0E2	OXT	RN7SK	LOC729926	NSUN5B
VDAC2	BID	SNCB	ATP6V1A	SNORD95	GNAQ	SNORA80	NGFR
VDAC3	BAD	SNCG	ATP6V1B1	RNU1G2	CACYBP	RAB11B	DPF1
AVEN	BAG1	LRRK2	ATP6V1B2	RNU1-3	RNU4-2	HS.545589	SCAND1
CARD10	BAG2	UCHL1	ATP6V1C1	RNU1-5	INPP5D	IL12A	RNU6-1
CARD11	BAG3	NR4A2	ATP6V1C2	LOC100130562	LOC642255	SNORD12C	LOC440258
CARD14	BAG4	ATP13A2	ATP6V1D	HS.537779	HS.582113	LOC642661	LOC100134364
CARD16	BAG5	AMBRA1	ATP6V1E1	DKFZP547K054	FOXS1	LOC100134468	TBX2
CARD17	TFAM	BECN1	ATP6V1E2	KSR1	RN5S9	OSAP	PRKAR1B
CARD18	TFAMP1	BECN1L1	ATP6V1F	LOC442041	ELAVL2	FSD1L	SESN3
CARD6	PPARG	BLOC1S1	ATP6V1G1	PSMC4	DRD3	GADD45A	POGZ
CARD8	PPARGC1A	BLOC1S2	ATP6V1G2	LOC139116	WASH3P	CDKN1A	RNU6-15
CARD9	PPARGC1B	BLOC1S3	ATP6V1G3	CNTD2	FADS2	SNORD13	NOVA2
CASKIN2	POLRMT	LAMP1	ATP6V1H	HS.564389	PPDPF	HS.562219	LOC389672
CASP1	MTERF	LAMP2	MMP1	LOC399942	LSM11	C7ORF20	LOC642962
CASP10	MTERFD1	LAMP3	MMP10	SNORA84	C17ORF89	HS.133410	BLOC1S2
CASP12	MTERFD2	LAPTM4A	MMP11	LOC641901	CDC2L1	SFXN1	RBM18
CASP14	MTERFD3	LAPTM4B	MMP12	SNORA28	LOC646330	LGALS3	CDC42EP1
CASP2	GABPAP	LAPTM5	MMP13	LOC730167	ALOX5AP	SLC25A36	GUCY1A2
CASP3	GABPB1	LYST	MMP14	RNU1A3	SNORD3D	FOSB	ZBTB11
CASP4	GABPB2	NPC1	MMP15	LOC100132564	LOC652255	LOC158301	HIP1R
CASP5	KEAP1	NPC1L1	MMP16	LOC100132394	HRK	PI4KA	RPPH1

CASP6	*NFE2*	*NPC2*	*MMP17*	*LOC652826*	*KCNJ4*	*HMOX1*	*LOC347544*
CASP7	*NFE2L1*	*EPM2A*	*MMP19*	*LOC389049*	*LOC440311*	*FLCN*	*TMEM8B*
CASP8	*NFE2L2*	*EPM2AIP1*	*MMP2*	*LOC441193*	*LOC653156*	*LOC730995*	*LOC100008588*
CASP8AP2	*NFE2L3*	*ATG10*	*MMP20*	*C1QTNF4*	*ISG20*	*CCRN4L*	*CDC34*
CASP9	*ATF1*	*ATG12*	*MMP21*	*RRAD*	*LOC643446*	*FAM46C*	*LOC100130276*
LOC650759	*ATF2*	*ATG16L1*	*MMP23A*	*LOC100131017*	*LOC728188*	*EPHA2*	*SNORD36A*
MCL1	*ATF3*	*ATG16L2*	*MMP23B*	*HIST1H2BJ*	*HERC5*	*CTSD*	*TCEB3*
BCL10	*ATF4*	*ATG2A*	*MMP24*	*LHX5*	*TNKS*	*LOC648931*	*C1ORF70*
BCL11A	*ATF5*	*ATG2B*	*MMP25*	*LOC100131323*	*PER1*	*LOC100130835*	*IER5L*
BCL11B	*ATF6*	*ATG3*	*MMP26*	*FAM108A3*	*UBTD1*	*PSMA7*	*WWP2*
BCL2	*ATF6B*	*ATG4A*	*MMP27*	*SPTBN1*	*PLK3*	*HIST3H2BB*	*RNU6ATAC*
BCL2A1	*ATF7*	*ATG4B*	*MMP28*	*TNFRSF12A*	*HSPBP1*	*RALGAPB*	*ITGA5*
BCL2L1	*ATF7IP*	*ATG4C*	*MMP3*	*GABPB1*	*RBM38*	*SH2B2*	*LOC727980*
BCL2L10	*ATF7IP2*	*ATG4D*	*MMP7*	*RNU11*	*SIRT7*	*HIST1H4E*	*NFKBIL1*
BCL2L11	*SIRT3*	*ATG5*	*MMP8*	*RELA*	*RAB30*	*LOC100134424*	*RARA*
BCL2L12	*PRKAA1*	*ATG7*	*MMP9*	*SNORD55*	*SDC4*	*UNCX*	*HSPA1B*
BCL2L13	*PRKAA2*	*ATG9A*	*MMPL1*	*LOC651149*	*RNU4ATAC*	*AKT1S1*	*SPIN1*
BCL2L14	*GCN1L1*	*ATG9B*	*LRRC4B*	*VTRNA1-2*	*PI4KAP1*	*TNRC4*	*C6ORF221*
BCL2L15	*MTFR1*	*NEU1*	*SDR39U1*	*DLG4*	*LOC345630*	*NSUN5C*	*ZNF787*
BCL2L2	*MFN1*	*SMPD1*	*BRAF*	*HSPA7*	*WASH5P*	*ZNF570*	*NOC2L*
BCL3	*MFN2*	*ATP6AP1*	*LOC100008589*	*SNORD3A*	*PMAIP1*	*NPAS4*	*NUCKS1*
BCL6	*OPA1*	*ATP6AP1L*	*LOC100133719*	*DCC*	*TRAF4*	*NACC2*	*BTG3*
BCL6B	*CRLS1*	*ATP6AP2*	*GIT2*	*KDM6B*	*CRYAB*	*CSNK2A1P*	*TCEA1*
BCL7A	*CRMP1*	*ATP6V0A1*	*SNORA63*	*RRN3P2*	*LOC100133950*	*RHOF*	*LOC346950*
	PARK2	*ATP6V0A2*					

Supplemental Table 5. Differential expression of mitochondria related genes in dopaminergic neurons derived from WT and *PARK2* $^{-/-}$ transgenic iPSC lines.

SYMBOL	*PARK2*$^{-/-}$ DA2	*PARK2-/+* DA2	WT DA2	*PARK2*$^{-/-}$ / WT DA2 Fold increase
NR4A2	984	205	172	5.71
NPAS4	202	217	39	5.24
COX11P	52	59	11	4.95
C1QTNF4	941	2267	218	4.31
NOVA2	550	858	135	4.09
ATF7IP	72	33	20	3.67
DPF1	2744	4382	874	3.14
LOC441193	42	13	14	2.95
TFAMP1	61	41	21	2.91
UCP3	94	67	36	2.62
LSM11	189	460	73	2.58
RARA	2218	1459	884	2.51
CDK5R2	455	1023	189	2.40
PDDC1	731	784	308	2.37
NDUFB1	224	331	97	2.31
SNCB	103	517	45	2.30
BLOC1S1	2388	1824	1043	2.29

SCAND1	6180	6422	2713	2.28
ISLR2	6080	1450	2742	2.22
WASH5P	305	363	139	2.20
ATP5D	9074	10479	4175	2.17
ATG16L2	151	121	70	2.17
BCL11B	2704	5161	1271	2.13
PRKAR1B	442	524	209	2.12
NDUFB7	12786	10745	6145	2.08

SYMBOL	***PARK2***$^{-/-}$ **DA2**	***PARK2***$^{-/+}$ **DA2**	**WT DA2**	***PARK2***$^{-/-}$ **/ WT DA2 Fold decrease**
CDKN1A	3956	4111	11598	0.34
ATF5	766	1037	3556	0.22
NEU1	779	959	2399	0.32
CRYAB	192	65	2005	0.10
ITGA5	185	247	1931	0.10
LAMP3	175	156	1559	0.11
HERC5	232	228	1262	0.18
TNFRSF12A	345	116	1201	0.29
CARD10	249	372	987	0.25
ATF3	91	190	893	0.10
EPHA2	146	116	818	0.18
ISG20	154	143	520	0.30
HMOX1	72	177	417	0.17
ATP10B	89	480	362	0.25
PMAIP1	44	8	350	0.13
VTRNA1-2	38	19	299	0.13
SNORA63	45	76	186	0.24
NAMPT	25	37	183	0.13
MMP10	18	29	151	0.12
IL12A	13	106	62	0.21

Supplemental Table 6. Differential expression of mitochondria related genes in *PARK2* KO neuron samples.

SYMBOL	***PARK2***$^{-/-}$	***PARK2***$^{-/+}$	**WT**	***PARK2***$^{-/-}$ **/ WT Fold increase**
NR4A2	441	117	30	14.61

GNAQ	352	124	26	13.44
LOC100131323	253	90	22	11.35
LOC399942	1513	465	145	10.40
CACYBP	494	197	52	9.49
LOC728188	567	229	66	8.63
ATP5E	8745	4462	1047	8.35
RPL12P6	1988	1096	259	7.66
LOC346950	809	507	111	7.28
UQCRH	13944	9048	2060	6.77
BLOC1S2	200	103	30	6.75
BLOC1S2	200	103	30	6.75
LOC100130562	2758	1548	438	6.29
LOC653156	10483	10347	1952	5.37
LOC100129685	8345	8142	1639	5.09
ATP7A	93	50	19	4.97
COX17	8464	4974	1772	4.78
COX6B1	17121	9309	3851	4.45
NDUFA12	13530	9681	3243	4.17
PSMC4	863	287	207	4.16
STT3B	103	77	25	4.09
NFE2L2	500	292	123	4.07
NDUFB6	5219	3280	1334	3.91
PPID	90	70	26	3.49
SNCA	776	249	225	3.45
LOC347544	16556	8657	4840	3.42
LOC730167	631	445	185	3.41
LOC652826	380	165	115	3.30
COX7B	6393	2713	1953	3.27
PPARGC1A	393	193	123	3.20
ELAVL2	404	198	130	3.10
LOC651149	4251	2128	1371	3.10
ATF2	318	268	104	3.06
ATP1B1	2984	2193	1027	2.90
SNORD36A	218	97	76	2.87
UQCRB	1408	945	493	2.86
BCL11B	726	508	256	2.84
ATP5EP2	40591	30696	14508	2.80
HRK	158	60	58	2.75
ATP5C1	6577	4742	2404	2.74
ATG4C	124	156	46	2.72
SNORD55	106	73	40	2.64
NAMPT	96	41	37	2.63
SLC25A36	369	205	146	2.53

COX5B	19434	12240	7708	2.52

SYMBOL	*PARK2*$^{-/-}$	*PARK2* $^{-/+}$	WT	*PARK2*$^{-/-}$ / WT Fold decrease
DPF1	426	403	1058	0.40
SNORD3A	262	336	659	0.40
ATP9B	45	115	113	0.40
LOC100131017	37	39	94	0.40
NSUN5B	58	46	149	0.39
NDUFV1	1992	1598	5080	0.39
CDC34	704	627	1801	0.39
ATG9A	211	265	542	0.39
UQCRC1	2805	3803	7240	0.39
SH2B2	170	123	438	0.39
LOC100130276	120	215	312	0.38
LOC642661	35	37	92	0.38
LOC100134424	97	75	254	0.38
LOC642255	43	33	118	0.37
GSK3B	737	1189	2026	0.36
LOC100134364	2330	2757	6409	0.36
UBTD1	120	155	337	0.36
SDHA	440	681	1245	0.35
ATP13A2	190	274	546	0.35
ATP13A2	190	274	546	0.35
PI4KAP1	84	67	246	0.34
DKFZp547K054	24	46	74	0.32
IER5L	151	145	467	0.32
COX19	302	444	947	0.32
LOC100132564	197	297	626	0.31
C1QTNF4	138	130	443	0.31
LRRC4B	71	52	227	0.31
WASH3P	32	33	103	0.31
NACC2	39	91	127	0.31
HIP1R	29	9	98	0.30
LOC389049	68	100	236	0.29
ATP13A1	304	498	1071	0.28
LOC729057	37	36	138	0.27
LOC100008588	2328	2449	8759	0.27
WASH5P	57	66	216	0.26
LOC100132394	3932	4986	15396	0.26
RNU4-2	250	312	1004	0.25
MMP15	178	247	736	0.24
NOVA2	63	54	264	0.24

LOC644284	33	54	142	0.23
UCP2	69	153	296	0.23
ATG4D	78	122	337	0.23
POLRMT	289	520	1362	0.21
MMP23B	18	18	84	0.21
ISLR2	371	133	1812	0.20
ITGA5	32	136	158	0.20
ATPBD3	17	60	97	0.17
RNU4ATAC	165	221	1018	0.16
LOC441193	13	53	81	0.16

Experimental procedures

Generation of iPSC lines from PD patients and controls

Fibroblasts were grown in Minimum Essential Medium Alpha, supplemented with 10-15% (line-specific) fetal bovine serum (FBS), and 1% antibiotic/antimycotic (all from Life Tech., NJ) under 3% O_2, 5% CO_2, 37°C in humidified chamber, and passaged every 5-6 days using TrypLE™ (Life Tech., NJ).

Reprogramming using Sendai virus (SeV, CytoTune™ SeV kit, Life Tech., NJ) was carried out following manufacturer's recommendations and previously described (Sivapatham and Zeng, 2014).

For spontaneous in vitro differentiation, iPSC were detached using collagenase. Cells were cultured in a suspension in ultra-low-attachment plates containing the EB differentiation medium (DMEM/F12 supplemented with StemPro supplement, BSA and FGF2). After 8 days in suspension culture, the EB were transferred to a gelatin-coated plate and cultured in the same medium for another 14 days prior to immunostaining.

Generation of *PARK2* KO isogenic lines by ZFN

ZFN expression plasmids targeting exon1 of *PARK2* gene were purchased from Sigma. Each ZFN polypeptide consists of two functional domains: the DNA binding domain (the recognition sequences of each ZFN are underlined) and the cleavage domain (FokI nuclease). After nucleofection of the *PARK2* ZFN pair in NCRM1, clones expanded from single cells in a 96-well plate were analyzed by DNA sequencing at the junction followed by sequence confirmation and verification of heterozygotes or homozygotes. Several clones with frame shift mutations close to exon 1 were expanded. A heterozygote of *PARK2* (*PARK2*$^{+/-}$) and a homozygote of *PARK2* (*PARK2*$^{-/-}$) was chosen for further analysis, and the mutations details along with the wild type (WT) sequences were shown in Fig. 5B.

MTT Assay

Cell viability was measured using an MTT assay, as previously described. (Peng et al., 2013). Briefly, cells grown in 48-well plates were maintained as required. Five mg/mL MTT tetrazolium salt was

added to each well, and incubated for 4 h at 37 °C. Crystals resulting from mitochondrial enzymatic activity on MTT substrate were solubilized with DMSO in 37 °C for 5 min. Absorbance was measured at 590 nm using a microplate reader (Molecular Devices, Sunnyvale, CA). Cell survival was measured in absorbance difference between treated and untreated cells.

Antibodies

The following primary antibodies were used: NESTIN (611658, BD Transduction laboratories, 1:500), TUJ-1 (clone SDL.3D10, T8660, Sigma, 1:1000), GFAP (Z0334, DakoCytomation, 1:2000), TH (P40101, Pel-Freeze, 1:500), TH (Mouse, 22941, ImmunoStar, 1:500), NANOG (14-5768-82 , eBioscience , 1:100), SOX2 (Ab1125, Abcam, 1:1000), FOXA2 (Ab40874, Abcam, 1:1000). SMA (A2547, Sigma, 1:500), AFP (A8452, Sigma, 1:500), TRA 1-60 (14-8863-82, eBioscience, 1:60), OCT4 (ab19857, Abcam, 1:1250), SOX2 (MAB4343, Millipore, 1:250), and Alkaline phosphatase staining kit II purchased from Stemgent.

Confocal microscopic stereology of mitochondria volume fraction

Control and patient-derived neuron precursors (at day 14 of differentiation) were cultured in 24 or 96 well cover glass-bottomed microplates. Cells were differentiated for another 14 days in PA6-CM in presence of BDNF and GDNF as described previously. On day 28, cultures were loaded with MitoTracker Red CMXRos (75 nM), calcein-AM (1µM) and Hoechst 33342 (5µg/ml) for 30 min and imaged on a Zeiss LSM 780 laser scanning confocal microscope in differentiation medium at 37°C and 5% CO_2. Using a Plan-Apochromat 63×/1.4 oil lens 1024×1024 pixel single planes were recorded at 44 nm pixel size at 1 Airy unit pinhole at recommended spectral settings. Using the Multi Time Series PLUS module of the ZEN 2011 software (Carl Zeiss, Jena, Germany) and Definite Focus autofocusing, first live cell images were acquired automatically along a 10×10 grid while systematically cycling *z*-focus. After fixation and labeling immunofluorescence of Alexa488-labeled TUJ-1 and Alexa647-labeled TH were recorded using stored coordinates and the Hoechst nuclear staining to register images with the live cell micrographs. Recordings were analyzed in Image Analyst MKII (Image Analyst Software, CA). The volume fraction was

calculated using image binarization and summing the number of pixels in all planes corresponding to mitochondrial and cellular profiles. Their ratio multiplied by the stereological correction factor of 2/3 considering projection of mitochondria into the optical thickness of the imaged plane provided the volume fraction (Gerencser et al., 2012). To gate the detection to dopaminergic neurons, regions of binarized images corresponding to the TH staining were manually outlined and the total numbers of pixels corresponding to mitochondrial and cellular profiles were obtained within these shapes. Importantly, a bias in V_F because of altered mitochondrial membrane potential is unlikely. Firstly, fluorescence of MitoTracker Red CMXRos is only partially potential-sensitive (because of lipid partitioning and self-quenching) and it has been shown to stain mitochondria with deficient respiration (Kukat et al., 2008Minamikawa et al., 1999). Secondly, the image processing pipeline performing binarization of MitoTracker images was designed to be little affected by variations in staining intensity (Gerencser et al., 2012).

Electron microscopy

Cells grown on a Thermanox (Nalgene Nunc International) coverslip were fixed for 30 min in 2% (w/v) paraformaldehyde and 2.5% (w/v) glutaraldehyde in 0.1 M sodium cacodylate. Cells were post-fixed in 1% (w/v) osmium tetroxide and 0.8% (w/v) potassium ferrocyanide in 0.1 M sodium cacodylate for 60 min, then stained with 2% (w/v) uranyl acetate for 30 min. Dehydrated and EMbed-812 infiltrated samples were embedded in EMbed-filled BEEM (Electron Microscopy Sciences,Hatfield, PA, USA) capsules at 60◦C for 72 h. Using an MT-7000 ultramicrotome, 70 nm-thick sections were generated and imaged on a Phillips Technai 12 transmission electron microscope at 80 kV at 68,000x.

Immunocytochemistry

The quantification of immunoreactive cells in culture was performed by analyzing fluorescent images using Adobe Photoshop. Cell counts were expressed as a percentage of total cells in a field. Total

number of cells was represented by the number of Hoechst-labeled nuclei on each image. Four different randomly chosen fields from four independent experiments were counted by three different individuals. Values were obtained by evaluating at least 600-750 TH-positive cells per experiment. Statistical analysis was performed using the Student's t-test with two-tailed distribution and assuming equal variance.

Whole genome expression analysis

The background method was used for normalization. The maximum expression value of gene for probe set was used as the expression value of the gene. For the processed data, the dendogram was represented by global array clustering of genes across all the experimental samples using the complete linkage method and measuring the Euclidian distance. Expression of sample correlations was a measure of Pearson's coefficient, implemented in R System.

qPCR analysis

Quantitative PCR reactions were carried out on the CFX96™ Touch Bio-Rad instrument (Bio-Rad, CA) using iTaq™ Universal SYBR® Green supermix (Bio-Rad, CA) according to the manufacturers' instructions. PCR reactions were conducted in duplicate or triplicate for each sample. Genomic DNA contamination and RNA quality were assayed using PrimePCR™ control assays (Bio-Rad, CA). For microarray validation experiments samples included Y9 (control), A6, A23 (*SNCA* triplication), I3, P1, B119, S110 (*PARK2* mutants), K20, K25 (*LRRK2* mutants), and T101 (*GBA* mutant) at dopaminergic stage (28 days of differentiation). Human *TBP, GAPDH,* and *ACTB* were amplified as internal standards. Reported values were calculated using ΔΔCt method and normalized against endogenous *ACTB* (pluripotency and SeV genes) or *TBP* and *GAPDH* (NSC and DA gene expression). Primer sequences were previously described(Sivapatham and Zeng, 2014).

Western blot analysis

Following SDS-polyacrylamide gel electrophoretic separation, proteins were transferred to 0.22 mm PVDF membrane (Bio-Rad, CA). Blocking was done in TBS with 5% milk and 0.1% Tween (all from Bio-

Rad, CA) for 1 hour at room temperature. Membranes were incubated with the following antibodies at $4^{o}C$ overnight: α-synuclein (BD Biosciences, 1:750), TH (Pel-Freeze, 1:1500), TH (Sigma, 1:1500), Horseradish peroxidase conjugated secondary antibodies (Life Tech., NJ) were diluted in blocking buffer and incubated for 1 hour at room temperature. Detection of bound antibodies was performed using ECL Advance Western Blotting Detection kit (Amersham Biosciences, NJ) and chemiluminescent signal was recorded on Hyperfilm (Amersham Biosciences, NJ).

References

Gerencser, A.A., Chinopoulos, C., Birket, M.J., Jastroch, M., Vitelli, C., Nicholls, D.G., and Brand, M.D. (2012). Quantitative measurement of mitochondrial membrane potential in cultured cells: calcium-induced de- and hyperpolarization of neuronal mitochondria. The Journal of physiology *590*, 2845-2871.

Peng, J., Liu, Q., Rao, M.S., and Zeng, X. (2013). Using human pluripotent stem cell-derived dopaminergic neurons to evaluate candidate Parkinson's disease therapeutic agents in MPP+ and rotenone models. Journal of biomolecular screening *18*, 522-533.

Sivapatham, R., and Zeng, X. (2014). Generation and Characterization of Patient-Specific Induced Pluripotent Stem Cell for Disease Modeling. Methods in molecular biology.
